# Selective targeting of genome amplifications and repeat elements by CRISPR-Cas9 nickases to promote cancer cell death

**DOI:** 10.1038/s41467-025-60160-2

**Published:** 2025-06-02

**Authors:** Matthew B. Hanlon, Jason M. Shohet, Scot A. Wolfe

**Affiliations:** 1 Department of Molecular, Cell and Cancer Biology, University of Massachusetts Chan Medical School, Worcester, MA 01605 USA; 2 Department of Pediatrics, University of Massachusetts Chan Medical School, Worcester, MA 01566 USA; 3 Li Weibo Institute for Rare Diseases Research, University of Massachusetts Chan Medical School, Worcester, MA USA

**Keywords:** Targeted therapies, CRISPR-Cas9 genome editing, DNA damage response

## Abstract

Focal gene amplification serves as an oncogenic driver during tumorigenesis and is a hallmark of many forms of cancer. Oncogene amplifications promote genomic instability, which is integral to cancer cell survival and evolution. However, focal gene amplification potentially affords an opportunity for therapeutic exploitation. As a proof-of-concept, we leverage CRISPR-Cas9 nickase to selectively promote cancer cell death in *MYCN*-amplified neuroblastoma in a gene amplification-dependent manner. Our analysis demonstrates that CRISPR-Cas9 nickase can generate a lethal number of highly toxic, replication-dependent double-strand breaks in cells harboring amplified loci. Furthermore, we demonstrate that Cas9 nickase—mediated toxicity can be modulated in combination with small molecule inhibitors targeting key regulators of the DNA-damage response or cell death pathways. Importantly, our findings in *MYCN*-amplified neuroblastoma translate to other cancer types with distinct oncogene amplifications.

## Abstract (web summary)

Focal gene amplification is a common feature of advanced and metastatic cancers. Here, the authors employ CRISPR-Cas9 nickase to target cancer-associated focal gene amplifications to selectively kill cancer cells through the production of DNA replication-associated double-strand breaks.

## Introduction

Neuroblastoma is an aggressive pediatric malignancy accounting for approximately 15% of childhood cancer mortality^1–3^. Due to a complex genetic landscape, clinical manifestations of neuroblastoma demonstrate a high degree of variability leading to a risk-based stratification scheme – low, intermediate, and high^4,5^. Currently, both low and intermediate-risk groups maintain excellent prognoses with an overall survival rate of >90%^6,7^. Despite recent advancements in cancer therapeutics, however, high-risk neuroblastomas remain a significant challenge to treat with an overall survival rate of ~50%^5^. The aggressive behavior demonstrated by high-risk neuroblastoma may be ascribed to segmental chromosomal aberrations, such as amplification of the *MYCN* oncogene^4^, which occurs in ~20% of neuroblastomas^8^. *MYCN* amplifications are highly heterogenous ranging anywhere from ~10 to >1000 copies, and may extend from 350 kb to 8 Mb depending on the site and stage of disease^9–12^.

High-risk neuroblastomas often respond to conventional therapeutic modalities such as dose-intensive chemotherapy, surgery, radiotherapy, myeloablative chemotherapy with subsequent hematopoietic stem-cell rescue, and anti-GD2 immunotherapy^2,4^. Regrettably, this intensive therapeutic regimen is frequently insufficient with relapse occurring in ≥50% of affected individuals with no curative salvage therapy currently available^4,5^. Of those that remain in remission, most experience significant treatment-associated morbidity: secondary malignancy, impaired growth and development, hearing loss, and infertility among others^3,13–16^. As such, the modest efficacy and detrimental side effects associated with conventional chemoradiotherapies in the context of high-risk neuroblastoma constitutes an urgent unmet medical need for novel therapeutic interventions.

Clustered regularly interspaced short palindromic repeats (CRISPR)-based genome editing systems have become an increasingly popular platform for the development of novel therapeutics^17,18^. CRISPR-based nuclease systems, such as Cas9 or Cas12a, are commonly utilized for the inactivation of genes or regulatory elements by means of the mutagenic, non-homologous end joining (NHEJ) DNA repair pathway^19^. While nuclease-based genome editing systems have shown promise for some therapeutic applications^20–22^, double-strand breaks (DSBs) can elicit unintended consequences such as large deletions, chromosomal truncations, translocations, or chromothripsis among other complex genomic rearrangements that may hinder its potential use for certain therapeutic applications^23–25^. Notably, Cas9 nuclease—induced cytotoxicity was observed in cancer cell lines when targeting amplified sequences in the context of large-scale gene inactivation screens^26–28^, which informed efforts to implement Cas9 nucleases for selective cell-killing by targeting cancer-specific sequence amplifications^29,30^.

Cas9 nickases, which are catalytically inactivated at either of the two cleavage domains (HNH or RuvC), have been implemented for the targeted generation of single-strand breaks (SSBs)^31^. Single-strand breaks are largely innocuous in post-mitotic cells, however persistent SSBs within proliferating cells can impose significant cellular toxicity^32,33^. In actively dividing cells, the conversion of SSBs into potentially toxic single-ended or double-ended DSBs (seDSB or deDSB, respectively) depending on encounter with leading or lagging strand synthesis during DNA replication can impose replication stress, replication fork collapse, and ultimately cell death if not adequately repaired^34–36^. For example, Cas9 nickases have been observed to promote cellular toxicity when targeted to endogenous, repetitive elements in the mammalian genome^33,37^.

**Fig. 1:**
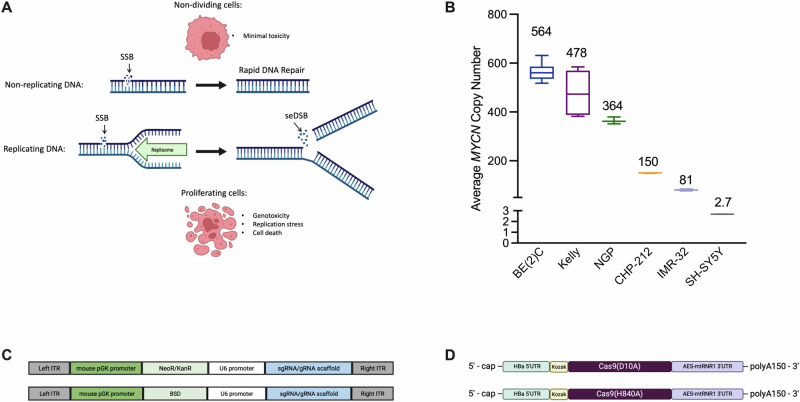
Generating lethal DNA damage in a genome amplification dependent manner. **A** Top pathway: Transient single-strand breaks (SSBs) are largely innocuous and quickly repaired in non-dividing cells. Bottom pathway: Persistent SSBs at high copy number loci within the genome in proliferating cells can induce cellular toxicity through their conversion into single-ended double-strand breaks (seDSBs) during DNA replication. Created in BioRender. Wolfe, S. (2025) https://BioRender.com/ttl9vxt. **B** Average *MYCN* genome copy number across various neuroblastoma cell lines obtained by qPCR (*n* = ≥ 3 biological replicates). For each box plot the horizontal line represents the median, the top and bottom of the box represent the upper and lower quartiles, respectively, and the top and bottom whiskers represent the maximum and minimum values, respectively. **C** A schematic of *piggyBac*-integrated sgRNA expression cassettes used to generate stable sgRNA-expressing cell lines. **D** A schematic of in vitro transcribed Cas9 nickase – mRNA construct variants. Created in BioRender. Wolfe, S. (2025) https://BioRender.com/tgt62po. Source data are provided as a Source Data file.

Here, we demonstrate that targeting highly amplified regions within the genome, such as *MYCN* in neuroblastoma cells, with the RuvC mutant SpyCas9^D10A^ nickase affords an opportunity to exploit genomic vulnerabilities to induce cell death in a gene amplification-dependent manner. We show that Cas9 nickase—mediated SSBs within amplified loci are subsequently converted to seDSB or deDSBs during DNA replication (Fig. 1A). Thus, only actively dividing, *MYCN*-amplified neuroblasts are exposed to a high level of genotoxicity, sparing copy-normal cells in the process. Additionally, we find that this nickase-based, genotype-specific toxicity is not limited to *MYCN*-amplified neuroblastoma; we also demonstrate its efficacy in additional malignancies that exhibit gene amplifications such as *ERBB2* (HER2)-amplified breast cancer, *MYC*-amplified non-small cell lung cancer, and *MYC*-amplified colorectal cancer.

## Results

### Cas9^D10A^ eliminates neuroblastoma cells in a gene amplification dependent manner

Given the high degree of heterogeneity found within neuroblastomas with respect to *MYCN* genome copy number^38,39^, a diverse cohort of neuroblastoma cell lines was selected for screening (Fig. 1B). The average *MYCN* genome copy number present in each cell line was validated by real-time quantitative PCR (qPCR; Fig. 1B) and was found to be in relative agreement with previously reported values^40^. Of the cell lines selected, CHP-212 is known to harbor *MYCN* amplification on extrachromosomal DNA (ecDNA)^41^ and SH-SY5Y serves as a *MYCN* non-amplified neuroblastoma control.

As previous work has shown, Cas9 nickases induce cellular toxicity when targeted to human long interspersed nuclear element (LINE1)^33^, an endogenous transposable element exceeding >5000 copies in human cell lines^42^. As such, Cas9 nickase targeting *LINE-1* served as a positive control for induced cellular toxicity. Cas9 nickase targeting *PPP1R12C* – AAVS1, a non-amplified safe harbor locus in the human genome^43^, served as a negative control. Empirically, we observed no appreciable difference in cell viability when targeting *AAVS1* compared to a panel of non-targeting or mock treatment controls (Supplementary Fig. 1). As a therapeutic target, we selected a non-coding region ~700 bp downstream of the annotated *MYCN* coding sequence that is still maintained within the *MYCN* amplicon to avoid any potential disruption of *MYCN* expression by means of coding-sequence alteration, as Cas9 nickases can introduce mutations at a low frequency^44,45^. To optimize screening, each cell line was modified to individually express one of the three guide RNAs (sgLINE-1, sgMYCN-1, or sgAAVS1) from a U6 promoter integrated as a stable transgene (Fig. 1C).

**Fig. 2:**
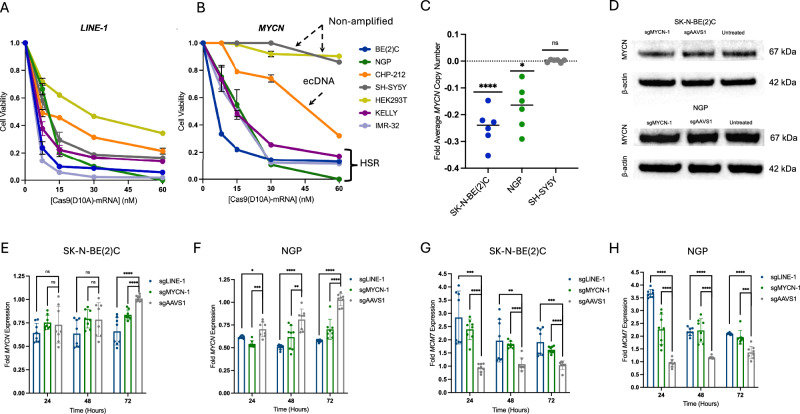
Cas9^D10A^ eliminates neuroblastoma cells in a dose dependent manner. **A** Positive control for Cas9^D10A^ nickase – mediated cell-killing. Long interspersed nuclear element 1 (LINE-1) is a widespread endogenous transposable element where there are >5000 canonical LINE-1 repeats in the reference human genome. Dose dependent nickase toxicity is observed in all cell lines expressing *LINE-1* targeting sgRNA (sgLINE-1) at 3 days post-treatment (*n* = 3 biological replicates). **B***MYCN*-amplified neuroblastoma cells expressing *MYCN* targeting sgRNA (sgMYCN-1) are rapidly depleted in a dose-dependent manner whereas *MYCN* non-amplified (SH-SY5Y) and non-neuroblastoma (HEK293T) cells expressing sgMYCN-1 are not appreciably affected at 3 days post-treatment (*n* = 3 biological replicates). Focal *MYCN* amplifications present as a homogeneously staining region (HSR) or ecDNA produce toxicity when targeted by Cas9 nickase. Data in panels (A and B are presented as mean ± s.d. and normalized relative to viability of cells expressing *AAVS1* targeting sgRNA treated with Cas9^D10A^. **C** *MYCN* copy number variance determined by real-time qPCR demonstrates a reduction in *MYCN* copy number 3 days following treatment with Cas9^D10A^-mRNA (30 nM) targeting *MYCN* in *MYCN*-amplified cell lines but no change in MYCN non-amplified SH-SY5Y cells (*n* = 3 biological replicates with technical duplicates). **D** Western blot of N-MYC protein expression levels from SK-N-BE(2)C or NGP cells expressing *MYCN* or *AAVS1* sgRNA at 24 h post-treatment with Cas9^D10A^. **E**, **F** *MYCN* expression analysis by qRT-PCR demonstrates a modest reduction in *MYCN* expression in SK-N-BE(2)C or NGP *MYCN*-amplified neuroblastoma cells up to 72-h post-treatment with Cas9^D10A^-mRNA (30 nM) when targeting *LINE-1* or *MYCN* (*n* = 4 biological replicates with technical duplicates). **G**, **H** *MCM7* expression analysis by qRT-PCR demonstrates an increase in *MCM7* expression in SK-N-BE(2)C or NGP *MYCN*-amplified neuroblastoma cells up to 72 h post-treatment with Cas9^D10A^-mRNA (30 nM) when targeting *LINE-1* or *MYCN* (*n* = 4 biological replicates with technical duplicates). Data are presented as individual data points around the mean ± s.d. and normalized to untreated cells as a baseline control. Data analyzed by multiple unpaired *t*-tests (two-tailed); ns, *P* > 0.05; *, *P* ≤ 0.05; ** *P *≤ 0.01; ***, *P *≤ 0.001; ****, *P *≤ 0.0001 relative to untreated cells. Source data are provided as a Source Data file.

For the initial assessment of Cas9 nickase mediated toxicity, each cell line was electroporated with a dose of in vitro transcribed Cas9^D10A^ – mRNA (Fig. 1D) ranging from 7.5 to 60 nM. *LINE-1* and *MYCN* targeted cells were subsequently assessed for changes in cell viability relative to *AAVS1* targeted cells after 3 days of incubation. Consistent with previous reports^33^, targeting of *LINE-1* with Cas9^D10A^ was toxic to all cell lines in a dose-dependent manner (Fig. 2A). Likewise, targeting a non-coding region adjacent to *MYCN* with Cas9^D10A^ led to the rapid depletion of *MYCN*-amplified neuroblastoma cell lines (SK-N-BE(2 C), KELLY, NGP, CHP-212, and IMR-32) in a dose-dependent manner (Fig. 2B). Conversely, targeting *MYCN* with Cas9^D10A^ was well-tolerated in *MYCN* non-amplified (SH-SY5Y) and *MYCN* non-amplified, non-neuroblastoma (HEK293T) cell lines (Fig. 2B).

Given these observations, all subsequent applications of Cas9^D10A^ – mRNA were carried out at a 30 nM dose, where a high therapeutic index for each of the target cell populations was observed. Next, to provide an orthogonal measure of Cas9^D10A^ toxicity when targeting amplified sequences, we assessed cell population growth by quantitative image-based cytometry (QIBC). Population dynamics were monitored over a 3 day period post-Cas9^D10A^ treatment (Supplementary Fig. 2). When targeting *LINE-1* with Cas9^D10A^, all cell lines demonstrated population collapse, whereas only *MYCN*-amplified neuroblastoma cell lines demonstrated population collapse when targeting *MYCN*. We did not observe any appreciable changes in population dynamics when targeting *AAVS1* relative to an untreated control.

Next, we sought to verify that the decrease in cell viability was not due to alterations in N-Myc levels. Despite targeting Cas9^D10A^ to a non-coding sequence downstream of the *MYCN* 3’UTR, we questioned whether Cas9^D10A^ – mediated DNA damage within *MYCN* amplifications could result in genomic or transcriptomic alterations within the target locus of the surviving cell population. Interestingly, *MYCN* copy number was observed to have decreased modestly in surviving *MYCN* targeted SK-N-BE(2)C and NGP cells ( ~ 25% and ~17%, respectively; Fig. 2C). Likewise, *MYCN* mRNA levels decreased when targeting *MYCN*, although similar reductions were observed in all Cas9^D10A^ treatment groups at early time points (Fig. 2E, F).

Despite the observed decrease in *MYCN* gene and transcript copy number when targeting *MYCN* with Cas9^D10A^ in SK-N-BE(2)C and NGP cells, no substantial differences in N-Myc levels were observed when targeting *MYCN* 48-h post-treatment with Cas9^D10A^ relative to *AAVS1* targeted or untreated cells (Fig. 2D). To assess N-Myc activity post-treatment with Cas9^D10A^, we assessed the expression of *MCM7*, a direct transcriptional target of N-Myc^46^ that functions to maintain genomic stability during S-phase^47^, at 1-, 2-, and 3 days post-treatment with Cas9^D10A^ (Fig. 2G, H). Consistent with the notion that Cas9^D10A^ – induced toxicity is associated with replication of DNA during S-phase, a substantial enrichment in *MCM7* expression was observed at 24-h post-treatment with Cas9^D10A^ when targeting *LINE-1* or *MYCN*, but not *AAVS1* (Fig. 2G, H). Expression of *MCM7* remained elevated, though decreased progressively at 48-, and 72-h. These observations suggest that targeting the amplified *MYCN* region does not appreciably affect N-Myc expression or activity.

Next, we asked whether the efficacy of Cas9^D10A^ could be target site-dependent with respect to the *MYCN* locus. As such, *MYCN*-amplified SK-N-BE(2)C and NGP cell lines were modified for the stable expression of sgRNAs targeting different regions within the *MYCN* locus: exon 2/intron (sgMYCN-2; ENST00000281043.4, ENST00000638417.1), exon 2 (sgMYCN-3) or the 3’-UTR (sgMYCN-4) (Supplementary Fig. 3A). No substantial position-specific variation in the efficacy of Cas9^D10A^—mediated cell-killing was observed in any *MYCN* sgRNA expressing cell line, supporting the notion that the observed cytotoxic effects conferred by Cas9^D10A^ are largely dependent on the number of available target sites and not the position of the target sequence within an amplified genomic region (Supplementary Fig. 3B).

We also examined whether the cellular toxicity induced by Cas9^D10A^ nickase when targeting an amplified gene could be replicated by the HNH mutant Cas9^H840A^ nickase^31^. As such, SK-N-BE(2)C, NGP, and SH-SY5Y cells were treated with Cas9^D10A^ or Cas9^H840A^—mRNA (30 nM) targeting *LINE-1*, *MYCN*, or *AAVS1* and assessed for changes in cell viability 3 days post-treatment. As anticipated, we observed that both Cas9^D10A^ and Cas9^H840A^ reduced cell viability when targeting *LINE-1* or *MYCN* in *MYCN*-amplified neuroblastoma cell lines (Supplementary Fig. 4). However, the reduction of cell viability was significantly greater in cells treated with Cas9^D10A^ nickase. The disproportionate efficacy between nickase variants may largely be due to superior cleavage activity of Cas9^D10A^ compared to Cas9^H840A^^48^ or a longer residence time of Cas9^D10A^ at the cleavage site, which may occlude the nick and prevent repair, due to preferential recognition and removal of Cas9^H840A^ intermediate by HLTF^35,49^.

### Cas9^D10A^ generates DSBs when targeted to amplified regions

**Fig. 3:**
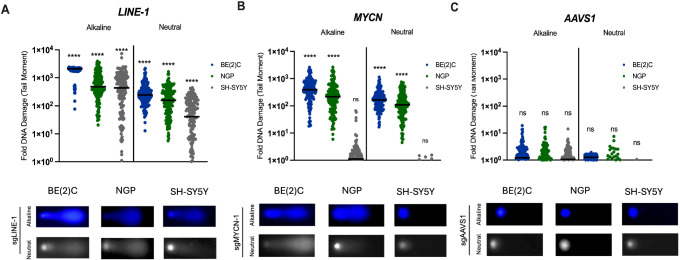
Cas9^D10A^ generates double-strand breaks at highly amplified loci in *MYCN*-amplified neuroblastoma cell lines. Cumulative DNA damage (alkaline) and DSBs (neutral) quantified by single cell gel electrophoresis (comet assay). Alkaline conditions (pH > 13) relax the supercoiled structure DNA allowing both SSBs and DSBs to be detected. Neutral conditions restrict detection to fragmented DNA from DSBs. **A**–**C** SK-N-BE(2)C, NGP, or SH-SY5Y cells expressing *LINE-1*, *MYCN* or *AAVS1* targeting sgRNA were electroporated with Cas9^D10A^-mRNA (30 nM). Individual cells were assessed for DNA damage at 3 days post-treatment by alkaline and neutral comet assay (*n* = 150). Representative images of Cas9^D10A^-treated cell comets are shown below each graph, respectively. Data are presented as individual data points around the median (black line) and were analyzed using a one-way ANOVA; ns, *P* > 0.05; *, *P *≤ 0.05; ** *P *≤ 0.01; ***, *P *≤ 0.001; ****, *P* ≤ 0.0001. Significance was determined using untreated cells as a baseline control. Source data are provided as a Source Data file.

We posited that the accumulation of SSBs generated by Cas9^D10A^, when intersecting with an active replication fork during S-phase, may lead to the formation of seDSBs or deDSBs as a function of the number of available Cas9^D10A^ target sites within the genome^35,36^. As such, the presence of DNA damage induced by Cas9^D10A^ within treated cells was assessed with single-cell resolution using the comet assay (Fig. 3; Supplementary Fig. 5)^50^.

Cumulative DNA damage (SSBs & DSBs) was assessed by comet assay under alkaline conditions at 3 days post-treatment with Cas9^D10A^ – mRNA (30 nM) in various neuroblastoma cell lines, as well as a non-neuroblastoma control cell line (HEK293T) that had been previously modified to express either *LINE-1*, *MYCN*, or *AAVS1* targeting sgRNAs. SK-N-BE(2)C, KELLY, NGP, CHP-212, SH-SY5Y and HEK293T cells demonstrated a dramatic increase in DNA damage (719–2677-fold) when targeting *LINE-1* (Fig. 3A; Supplementary Fig. 5A). Cell lines harboring *MYCN* amplification (SK-N-BE(2)C, KELLY, NGP and CHP-212) demonstrated a substantial increase in DNA damage (122 to 746-fold) when targeting *MYCN*, whereas cell lines without *MYCN* amplification (SH-SY5Y and HEK293T) demonstrated only a modest increase in DNA damage (2.1 to 2.7-fold) when targeting *MYCN* (Fig. 3B; Supplementary Fig. 5B), which was similar to the increase in DNA damage (1.1 to 2.4-fold) when targeting *AAVS1* (Fig. 3C; Supplementary Fig. 5C).

Double-strand breaks were specifically assessed by comet assay under neutral conditions for the same treatment groups (Fig. 3). Under these conditions, cells targeted at *LINE-1* demonstrated a substantial increase in DSBs (83 – 646-fold; Fig. 3A; Supplementary Fig. 5A). Likewise, *MYCN*-amplified cell lines demonstrated a similar increase in DSBs (102 to 381-fold) when targeting *MYCN*, whereas cell lines without *MYCN* amplification (SH-SY5Y and HEK293T) demonstrated a modest increase in DSBs (0.3 – 0.6-fold) when targeting *MYCN* (Fig. 3B; Supplementary Fig. 5B) similar to the increase in DSBs (0.2 to 1.3-fold) when targeting *AAVS1* relative to a mock/untreated control (Fig. 3C; Supplementary Fig. 5C).

### Cas9^D10A^ – mediated DSBs induce replication stress and G_2_/M cell-cycle arrest

**Fig. 4:**
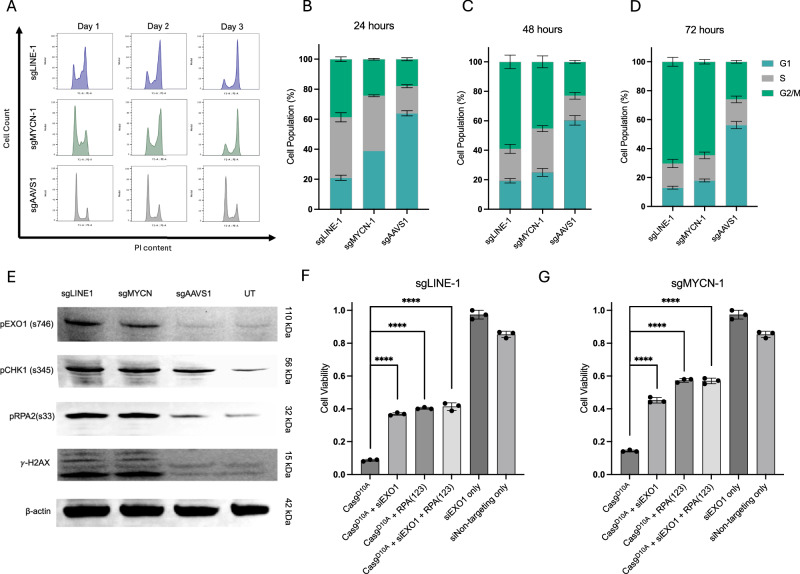
Cas9^D10A^-mediated DNA damage promotes replication stress, hyperactivation of the ATR- mediated DNA damage repair pathway and G_2_/M cell cycle arrest. **A**–**D** Flow cytometric cell cycle analysis of SK-N-BE(2)C cells expressing *LINE-1*, *MYCN* or *AAVS1* targeting sgRNA at 1-, 2-, and 3 days post-treatment with Cas9^D10A^ mRNA (30 nM). *LINE-1* and *MYCN* targeted cells display an extension of S-phase at 1 day post-treatment and eventual arrest in G_2_/M at days 2 and 3 (*n* = 3 biological replicates). *AAVS1* targeted cells demonstrate no apparent alteration in cell cycle progression. Data in panels (**B**–**D**) are presented as mean ± s.d. **E** Western blot of DNA damage markers from SK-N-BE(2)C cells expressing *LINE-1*, *MYCN* or *AAVS1* sgRNA at 3 days post-treatment with Cas9^D10A^. *LINE-1* and *MYCN* targeted cells demonstrate a substantial elevation in DNA damage markers. **F**, **G** Cell viability assessment for SK-N-BE(2)C, or SK-N-BE(2)C-RPA(123) cells expressing (**F**) *LINE-1* or (**G**) *MYCN* targeting sgRNA treated with Cas9^D10A^, Cas9^D10A^ + siRNA, or siRNA only. Cells treated with both Cas9^D10A^ and siRNA were pre-incubated with siRNA for 24 h prior to the delivery of Cas9^D10A^ – mRNA (30 nM). For each condition, cell viability was assessed at 3 days post-treatment (*n* = 3 biological replicates). Data are presented as mean ± s.d. normalized relative to viability of cells expressing *AAVS1* targeting sgRNA treated with Cas9^D10A^. Data were analyzed multiple unpaired *t*-tests (two-tailed); ns, *P* > 0.05; *, *P *≤ 0.05; ** *P* ≤ 0.01; ***, *P* ≤ 0.001; ****, *P* ≤ 0.0001. Source data are provided as a Source Data file.

Given that the conversion of Cas9^D10A^-generated SSBs to DSBs is contingent on active replication of the genome, we anticipated cell cycle aberrations to occur in S-phase. Consistent with our expectations, cell cycle analysis of *MYCN*-amplified SK-N-BE(2)C cells revealed a substantial accumulation of cells in S-phase at 24-h post-treatment with Cas9^D10A^ when targeting *LINE-1* or *MYCN* (2.2 & 2-fold, respectively; Fig. 4A, B).

Replication stress was demonstratively enriched in all cell lines targeted at *LINE-*1 and in *MYCN*-amplified cell lines targeted at *MYCN* 24 h post-treatment with Cas9^D10A^, as assessed by elevated of cytosolic calcium (Ca^2+^) levels (Supplementary Fig. 6A–C)^51^. Additional indicators of replication stress that are congruent with fork-stalling as a means to delay cell cycle progression and mitigate fork breakage within S-phase observed in these Cas9^D10A^-treated cell populations, include both the elevation of reactive oxygen species (ROS; Supplementary Fig. 6 D-F)^52–54^, and the increase in ATR-mediated phosphorylation of RPA2 (pS33) and CHK1 (pS345; Fig. 4E)^55–57^. Moreover, the production of DSBs during replication when Cas9^D10A^ targets *LINE-*1 or *MYCN* in SK-N-BE(2) cells is evident by elevated levels of the canonical biomarker γ-H2AX (Fig. 4E)^58–60^.

Excessive DNA resection by EXO1 is known to propagate genomic instability and toxicity by hindering DSB repair^61,62^. Phosphorylation of EXO1 (pS746) by the ATR-CHK1 or CMKK2-AMPK pathways in response to replication stress protects DNA ends exposed by DSBs by preventing DNA resection^51^. Following Cas9^D10A^ treatment, we observed an increase in phosphorylated EXO1 (pS746) in SK-N-BE(2)C cells when targeting *LINE-1* or *MYCN* but not *AAVS1* (Fig. 4E). DNA2 is partially redundant with EXO1 for DNA resection during S-phase DNA damage repair^63^. We examined whether EXO1 or DNA2 activity may be contributing to the observed toxicity conferred by Cas9^D10A^ when targeting amplified loci. To investigate this, SK-N-BE(2)C cells expressing *LINE-1*, *MYCN*, or *AAVS1* targeting sgRNAs were pre-treated with EXO1 (siEXO1), DNA2 (siDNA2), or a non-targeting (siNT) siRNA and recovered for 24-h before delivering Cas9^D10A^. Notably, the downregulation of EXO1, but not DNA2 was observed to increase cell viability by 3 to 4-fold for Cas9^D10A^ targeting *LINE-1* or *MYCN* (Fig. 4F, G; Supplementary Fig. 7). Likewise, the downregulation of EXO1 was shown to have a modest positive effect on cell proliferation for Cas9^D10A^ targeting *LINE-1* or *MYCN* in SK-N-BE(2)C cells (Supplementary Fig. 8A–C).

In the absence of Cas9^D10A^, cells treated with siEXO1 displayed appreciable stalling in S-phase of the cell cycle after 48-h (Supplementary Fig. 8D–F). Conceivably, the increase observed in cell viability in SK-N-BE(2)C cells treated with Cas9^D10A^ targeting *LINE-1* or *MYCN* may be due to slower rates of cell proliferation or a decrease in extensive 5’ DNA resection due to the reduction of EXO1 activity. As such, we reasoned these siEXO1-treated cells may display an alteration in target site sequence modifications due to changes in their ability to resolve DNA damage induced by Cas9^D10A^ during replication. Evaluation of the *MYCN* target site in Cas9^D10A^-treated, siEXO1+ SK-N-BE(2)C cells by amplicon-sequencing revealed ~15% editing rate, whereas Cas9^D10A^, siDNA2+ SK-N-BE(2)C cells revealed a modest ~1% editing rate, relative to an untreated control (Supplementary Fig. 9). Our observations suggest that EXO1 activity may play a contributing role in Cas9^D10A^-mediated cell-killing.

Overexpression of RPA has been shown to fortify cells from the catastrophic impacts of replication stress by modulating exonuclease activity^57,64,65^. Previous work indicated that a free RPA reservoir is critical for the protection of replication forks against breakage during replication stress^57^, exhaustion of which promotes and accelerates fork collapse. As such, we suspected that the extensive number of Cas9^D10A^-mediated SSBs may deplete the free RPA reservoir, potentially exacerbating Cas9^D10A^-induced cellular toxicity. To investigate this, SK-N-BE(2)C cells expressing *LINE-1* or *MYCN* targeting sgRNA were modified to overexpress human RPA1, RPA2, and RPA3 from a synthetic CAG promoter as a stable transgene (Supplementary Fig. 10) and subsequently treated with Cas9^D10A^. Interestingly, the overexpression of RPA increased survival in Cas9^D10A^ – treated, SK-N-BE(2)C cells when targeting either *LINE-1* or *MYCN* (4 to 5-fold) but remained insufficient to completely abolish cellular toxicity (Fig. 4F, G).

Given the partial increase in cell viability observed by the downregulation of EXO1 or the overexpression of RPA independently, we reasoned combining these approaches may increase resistance against Cas9^D10A^-mediated DNA damage in an additive manner. To test this, RPA-overexpressing SK-N-BE(2)C cells expressing *LINE-1* or *MYCN* targeting sgRNA were pre-treated with siEXO1 24 h prior to Cas9^D10A^ delivery. Surprisingly, no additional improvement in cell viability was observed (Fig. 4F, G), suggesting that these two perturbations (EXO1 knockdown and RPA overexpression) are impacting the same cytotoxic DNA repair intermediate associated with excessive DNA resection.

Furthermore, we observed stalling in S-phase to be only a transient state in response to Cas9^D10A^-mediated nicking at *LINE-1* or *MYCN*, as evident by the progression into, and subsequent arrest of SK-N-BE(2)C cells in G_2_/M-phase of the cell cycle at days 2 and 3 post-treatment, with a failure to repopulate G1 (Fig. 4A, C and D). As such, we speculated that cell death is likely occurring shortly after cell division. Flow cytometric analysis of surviving SK-N-BE(2)C cells 3 days post-treatment with Cas9^D10A^ targeting *LINE-1* or *MYCN* revealed a substantial increase in cells harboring micronuclei (4.9 and 4.6-fold, respectively), whereas *AAVS1* targeted cells showed only a modest increase (1.3-fold) relative to a mock/untreated control (Supplementary Fig. 11A, B).

Provided that micronuclei formation is contingent on the mitotic entry of cells harboring unrepaired DNA damage^66^, we asked whether Cas9^D10A^-mediated cell-killing is dependent on cell division. As such, SK-N-BE(2)C, NGP, and SH-SY5Y cells treated with Cas9^D10A^ targeting *LINE-1*, *MYCN*, and *AAVS1* were supplemented with the AURKA inhibitor (AURKAi), alisertib (0.5 µM), known to promote mitotic arrest^67^. Remarkably, an AURKAi significantly attenuated the cytotoxic effects conferred by Cas9^D10A^-mediated DNA damage (Supplementary Fig. 11C, D). These findings suggest that cell death in Cas9^D10A^-treated cells may occur subsequent to a mitotic catastrophe, which is consistent with the observed arrest of Cas9^D10A^-treated cells in the G2/M phase of the cell cycle, and a failure to repopulate G1.

### Cas9^D10A^-induced DNA damage promotes PARP1 hyperactivation and necrotic cell death in MYCN-amplified neuroblastoma cells

The vast majority of primary neuroblastomas express wild type (WT) p53, with less than 2% harboring a p53 inactivating mutation^68–70^. In neuroblastoma, *MYCN*-driven tumorigenesis is contingent upon inhibition of p53-mediated apoptosis through the upregulation of MDM2^70^. To assess the potential role of core apoptosis factors in cell death resulting from Cas9^D10A^-induced DNA damage, we screened two p53-WT neuroblastoma cell lines (*MYCN*-amplified NGP and *MYCN* non-amplified SH-SY5Y)^71^ for caspase 3 activation, a hallmark of intrinsic apoptosis^72,73^. Caspase 3 cleavage was evaluated by Western blot in both NGP and SH-SY5Y cells 3 days post-treatment with Cas9^D10A^ and targeting *LINE-1, MYCN*, or *AAVS1* (Supplementary Fig. 12A). As a positive control, SH-SY5Y cells were co-incubated with staurosporine (STS; 1 µM), a known inducer of caspase activation^74,75^. Not surprisingly, caspase 3 activation was readily apparent in STS-treated SH-SY5Y cells, whereas no obvious caspase 3 activation was detected in either cell line when targeting *LINE-1*, *MYCN*, or *AAVS1* with Cas9^D10A^. To further validate our observations, we co-treated *MYCN*-amplified SK-N-BE(2)C (p53-mutant) and NGP (p53-WT) cells with Cas9^D10A^ targeting either *LINE-1*, *MYCN*, or *AAVS1* and Z-DEVD-FMK (18 µM), a known caspase 3 inhibitor, or pifithrin-α (PFT-α; 20 µM), a presumed inhibitor of p53 activity and apoptosis (Supplementary Fig. 12B, C)^76,77^. No appreciable difference in the efficacy of Cas9^D10A^ – mediated cell-killing was observed.

Continued investigation of cell death pathways activated by Cas9^D10A^ – mediated DNA damage was carried out in the p53-deficient, highly *MYCN*-amplified neuroblastoma cell line, SK-N-BE(2)C. Poly [ADP-ribose] polymerase 1 (PARP1) cleavage is a common occurrence observed across various cell death pathways, displaying distinct cleavage patterns by which proteases involved in each pathway can be identified^78^. As such, PARP1 cleavage was used as a benchmark to provide mechanistic insight into the cell death pathway(s) stimulated by Cas9^D10A^. As expected, when targeting *LINE-1* and *MYCN* in SK-N-BE(2)C cells with Cas9^D10A^ we observed substantially more PARP1 cleavage compared to targeting *AAVS1* (Supplementary Fig. 12D). Multiple cleavage fragments ranging from 40 – 70 kDa were present, a pattern indicative of calpain-mediated PARP1 cleavage with continued proteolysis by cathepsin proteases^79,80^ and consistent with necrotic cell death.

Calpains are a family of Ca^2+^-activated cysteine proteases^81^. Their activation would be consistent with the elevation in cytosolic Ca^2+^ observed upon treatment of cells with Cas9^D10A^ when targeting amplified loci (See, Supplementary Fig. 6A–C). Calpain activation, as marked by autoproteolytic cleavage^82,83^, was readily apparent in both *LINE-1* and *MYCN* targeted SK-N-BE(2)C cell lysates (Supplementary Fig. 13A). Expanding on this observation, we assessed the level of calpain activity in both *MYCN*-amplified, SK-N-BE(2)C and NGP *LINE-1*, *MYCN*, or *AAVS1* sgRNA expressing cells at 3 days post-treatment with Cas9^D10A^-mRNA (30 nM) relative to a mock/untreated control. As a positive control, calpain activity was induced in both SK-N-BE(2)C and NGP cells by preincubation in growth media supplemented with CaCl_2_ (2 mM). Consistent with the autoproteolytic cleavage observed previously, cells targeted by Cas9^D10A^ at either *LINE-1* or *MYCN* demonstrated an increase in calpain activity in both SK-N-BE(2)C (2.7 & 2.4—fold) and NGP (2.5 & 2.2—fold) cells (Supplementary Fig. 13B). Calpain activity was not observed to be appreciably altered in either SK-N-BE(2)C or NGP cells when targeting *AAVS1* (1.0 & 1.1—fold).

Given the observed activation of calpains post-treatment with Cas9^D10A^, we reasoned that calpain inhibition may promote cell survival. As such, SK-N-BE(2)C, NGP, and SH-SY5Y cells treated with Cas9^D10A^ targeting *LINE-1*, *MYCN* or *AAVS1* were supplemented with calpastatin (CAST; 20 nM) an endogenous calpain inhibitor^84^. CAST supplementation in the presence of Cas9^D10A^ significantly promoted cell survival (Supplementary Figs. 13C, D; 14A, B), while CAST supplementation in the absence of Cas9^D10A^ had no effect on cell proliferation (See, Supplementary Fig. 15). These observations implicate calpains as a key protease involved in the execution of cell death post-treatment with Cas9^D10A^ targeting amplified loci.

Ca^2+^ and ROS are key factors governing both the induction, and execution of necrotic cell death^85–87^. Furthermore, the influx of Ca^2+^ and ROS are known to elicit PARP1 hyperactivation subsequent to DNA damage^88–90^. PARP1 hyperactivation is marked by the enrichment of poly-ADP ribosylation (PARylation) of endogenous proteins and segments of damaged DNA, resulting in the depletion of intracellular ATP and NAD^+^ pools and the promotion of necrotic cell death^78,91–97^. As such, we observed a stark elevation in PARylation in SK-N-BE(2)C cells when targeting *LINE-1* or *MYCN* with Cas9^D10A^ (Supplementary Fig. 12D). Moreover, the depletion of ATP and NAD^+^ levels were observed across all tested neuroblastoma cell lines (SK-N-BE(2)C, NGP, and SH-SY5Y) when targeting *LINE-1* or when targeting *MYCN* in *MYCN*-amplified neuroblastoma cell lines (SK-N-BE(2)C, and NGP; Supplementary Fig. 16).

Given these observations, we investigated the effect of PARP inhibition on Cas9^D10A^-mediated cell-killing in *MYCN*-amplified (SK-N-BE(2)C and NGP) and *MYCN* non-amplified (SH-SY5Y) neuroblastoma cells. Treatment with the PARP1 inhibitor (PARPi) rucaparib (10 µM) significantly attenuated ATP and NAD^+^ depletion (Supplementary Fig. 16) and promoted cell survival in the presence of Cas9^D10A^ when targeting *LINE-1* or *MYCN* (Supplementary Figs. 17A, B; 14C, D). In the absence of Cas9^D10A^, cell proliferation was only modestly affected by rucaparib treatment (See, Supplementary Fig. 12). Similar inhibition of Cas9^D10A^ cell killing was observed with a second PARPi, olaparib (10 µM), which has a different allosteric effect on PARP1 recognition of DNA lesions^98^ (Supplementary Fig. 17C, D). Consistent with the modest change in cell cycle progression in PARPi-treated cells, no substantial reduction in the DNA damaging activity was observed for Cas9^D10A^ targeting amplified loci (Supplementary Fig. 17E). Additionally, we observed only a modest increase in the fraction of cells in the G2/M phase of the cell cycle after 24-h of incubation with either rucaparib or olaparib relative to an untreated control (Supplementary Fig. 18). As such, these data suggest that the increase in cell survival observed when PARPi are combined with Cas9^D10A^ targeting amplified loci is likely due to a reduction in PARP1 activity. These observations are consistent with the extensive Cas9^D10A^-mediated DNA damage occurring during DNA replication leading to PARP1 hyperactivation followed by necrotic cell death.

Release of cellular contents, such as the cytosolic enzyme lactate dehydrogenase (LDH), through permeabilization of the plasma membrane is a hallmark of necrotic cell death^99^. As such, we assessed the amount of LDH present in the cell culture media in both SK-N-BE(2)C and NGP *LINE-1*, *MYCN*, or *AAVS1* sgRNA expressing cells at 3 days post-treatment with Cas9^D10A^ – mRNA (30 nM; Supplementary Fig. 19). Consistent with plasma membrane disruption, LDH activity was increased when targeting *LINE-1* or *MYCN* in SK-N-BE(2)C and NGP cells by ~3-fold, whereas LDH activity was not appreciable altered when targeting *AAVS1* relative to an untreated control. Collectively, these observations suggest necrosis as the principal mode of cell death following Cas9^D10A^—mediated DNA damage at amplified loci within the genome.

### Surviving Cas9^D10A^-treated neuroblastoma cells display markers of neuronal differentiation

Though Cas9^D10A^ demonstrates robust cell-killing efficiency across a variety of neuroblastoma cell lines, a small percentage of cells manage to survive. As such, we were interested in expanding the surviving cell population as a means of testing their susceptibility to retreatment with Cas9^D10A^. However, the outgrowth of the surviving cell population failed on numerous occasions. Moreover, the surviving cells displayed morphological changes, such as de novo neurite formation and extension, consistent with neuronal differentiation^100,101^ (Supplementary Fig. 20A). DNA damage—induced cell differentiation has previously be reported to occur in stem cells^102–104^. Given the stem cell-like properties associated with neuroblastomas^105,106^, we explored whether Cas9^D10A^—induced DNA damage prompted the surviving cell population to undergo neuronal differentiation.

Here, we treated the *MYCN-*amplified, SK-N-BE(2)C cell line with Cas9^D10A^ – mRNA (30 nM) targeting *MYCN* or *AAVS1*. Cells were assessed by immunocytochemistry for canonical biomarkers of neuronal differentiation, such as the elevation of class III β-tubulin (TUBB3) and a reduction in the proliferation marker, Ki-67^107^ at 5 days post-treatment (Supplementary Fig. 20B, C). SK-N-BE(2)C cells undergo neuronal differentiation when treated with retinoic acid^107^. Thus, untreated SK-N-BE(2)C cells were co-incubated with all-trans-retinoic acid (ATRA; 10 µM) as a positive control. Consistent with neuronal differentiation, SK-N-BE(2)C cells treated with *MYCN* targeting Cas9^D10A^ displayed an enrichment in TUBB3 expression ( ~ 6.2-fold) and decrease in Ki-67 ( ~ 2.9-fold), whereas no appreciable differences were observed when targeting *AAVS1* relative to an untreated control (Supplementary Fig. 20B, C). These results indicate that a fraction of the cell population may exit the cell cycle following treatment with Cas9^D10A^ targeting the amplified *MYCN* locus to promote cell survival during a period of high cellular stress^108^. Whether this is a transient or terminal induction of neuronal differentiation is unclear.

### Cas9^D10A^-mediated cell-killing can be augmented in combination with DDR pathway inhibitors or multiplex targeting

ATR and CHK1 inhibitors are commonly employed to potentiate the effectiveness of genotoxic chemotherapeutics by promoting continued origin firing and G_2_/M checkpoint escape^109–112^. The ATR-CHK1 DNA-damage response axis plays a critical role in restraining DNA replication in the context of replication stress by halting new origin firing and restricting EXO1-mediated DNA resection activity^45,51^. Therefore, we reasoned that the inhibition of ATR or CHK1 may yield a cellular environment that is more permissive to the accumulation of Cas9^D10A^-mediated DNA damage during S-phase, bypassing the G_2_/M-checkpoint, and aberrantly allowing for mitotic entry with unrepaired DNA damage.

**Fig. 5:**
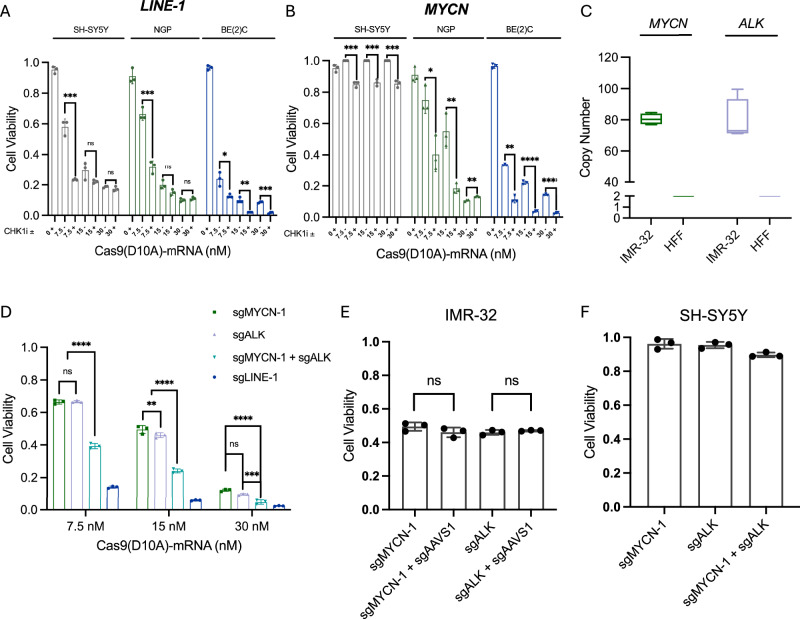
Cellular toxicity of Cas9^D10A^ in *MYCN*-amplified neuroblastoma cells is augmented by CHK1 inhibitors or multiplex targeting. **A**, **B** SK-N-BE(2)C, NGP, and SH-SY5Y cells expressing (**A**) *LINE-1* or (**B**) *MYCN* targeting sgRNA were treated with Cas9^D10A^-mRNA at increasing doses in the absence or presence of a CHK1 inhibitor, MK8776 (500 nM). Co-treatment with CHK1i potentiated Cas9^D10A^-mediated cell-killing across cell lines at 3 days post-treatment (*n* = 3 biological replicates). Data were analyzed by multiple unpaired *t*-tests (two-tailed); ns, *P* > 0.05; *P ≤ 0.05; **P ≤ 0.01; ***P ≤ 0.001; ****P ≤ 0.0001. **C** Average *MYCN* and *ALK* genome copy number in IMR-32 cells determined by qPCR (*n* = 4 biological replicates). Data are presented normalized to HFF cells as a baseline control where for each box plot the horizontal line represents the median, the top and bottom of the box represent the upper and lower quartiles, respectively, and the top and bottom whiskers represent the maximum and minimum values, respectively. **D** Targeting of *LINE-1*, *MYCN*, *ALK*, or *MYCN* and *ALK* in IMR-32 cells (2 × 105) with Cas9^D10A^ demonstrates a dose-dependent cytotoxic effect. Multiplex targeting of *MYCN* and *ALK* increases Cas9^D10A^-mediated cell-killing relative to targeting *MYCN* or *ALK* individually (*n* = 3 biological replicates). Data were analyzed using a two-way ANOVA with a Tukey’s multiple comparison test; ns, *P* > 0.05; *P ≤ 0.05; **P ≤ 0.01; ***P ≤ 0.001; ****P ≤ 0.0001. **E** No additional cellular toxicity is observed for multiplex targeting of an amplified (*MYCN* or *ALK*) locus with a non-amplified (*AAVS1*) locus in IMR-32 cells with Cas9^D10A^-mRNA (15 nM; *n* = 3 biological replicates). Data were analyzed by multiple unpaired *t*-tests (two-tailed); ns, *P* > 0.05; *P ≤ 0.05; **P ≤ 0.01; ***P ≤ 0.001; ****P ≤ 0.0001. **F** Multiplex targeting of non-amplified loci in SH-SY5Y cells with Cas9^D10A^-mRNA (30 nM) resulted in similar rates of cell viability to targeting individual loci. Cells were assessed for changes in cell viability at 3 days post-treatment with Cas9^D10A^-mRNA (*n* = 3). Data in panels (**A**, **B**, and **E**, **F**) is presented as mean ± s.d. and normalized relative to viability of cells expressing *AAVS1* targeting sgRNA treated with Cas9^D10A^. Source data are provided as a Source Data file.

To test this combination approach, we supplemented Cas9^D10A^ treatment with a sub-lethal concentration (≤IC_50_) of the CHK1 inhibitor (CHK1i), MK8776 (500 nM) or the ATR inhibitor (ATRi), berzosertib (M6620, VX-970, VE-822; 20 nM)^112,113^ and assessed changes in cell viability 3 days post-treatment. Combining Cas9^D10A^ and CHK1i or ATRi was shown to potentiate cell killing across all cell lines when targeting *LINE-1*, and significantly improved efficacy when targeting *MYCN* within *MYCN*-amplified cell lines at lower concentrations of Cas9^D10A^—mRNA (Fig. 5A, B; Supplementary Figs. 21, 22). Notably, only a modest reduction in cell viability was observed when targeting *MYCN* within the *MYCN* non-amplified SH-SY5Y cell line in the presence of CHK1i or ATRi which may be ascribed to the toxicity of either small molecule inhibitor in the context of dysregulated cellular proliferation in neuroblastoma cell lines (See, Supplementary Fig. 15).

Co-amplification of oncogenes with proximal non-coding regulatory regions, such as enhancers, or even additional genes or gene segments is a phenomenon readily observed across tumor types^114^. In addition, amplification of distant genes can also occur. For example, focal amplifications of the receptor tyrosine kinase gene, *ALK*, located ~14 Mb from *MYCN* (Fig. 5C), have been observed in a subset of *MYCN*-amplified neuroblastomas^115^. As such, we reasoned gene amplifications from distinct genomic regions could be targeted simultaneously to further enhance Cas9^D10A^-mediated cell-killing by increasing the amount of associated DNA damage. To test this hypothesis, we utilized the IMR-32 neuroblastoma cell line harboring both *MYCN* and *ALK* gene amplification, which contains ~80 genome copies of each gene^40^ (Fig. 5C). We reasoned that targeting both *MYCN* and *ALK* amplifications simultaneously, which would double the number of Cas9^D10A^ target sites, should have an additive effect. IMR-32 cells were modified in the same manner as previously described to individually express one of four guide RNAs (sgLINE-1, sgMYCN-1, sgALK or sgAAVS1), or co-express both sgMYCN-1 and sgALK simultaneously. Like sgMYCN-1, sgALK was designed to target a non-coding region neighboring the 3’ UTR of *ALK*.

Similar to other Cas9^D10A^-treated neuroblastoma cell lines, the efficacy of Cas9^D10A^-mediated cell-killing in IMR-32 cells increased in a dose-dependent manner (Fig. 5D). Given the similarity in *MYCN* and *ALK* gene copy number in IMR-32 cells, we observed a comparable decrease in cell viability when targeting *MYCN* or *ALK* at each of the tested concentrations of Cas9^D10A^-mRNA (Fig. 5D). However, when targeting *MYCN* and *ALK* simultaneously we observed an appreciable increase in the efficacy of Cas9^D10A^-mediated cell-killing at each of the tested concentrations of Cas9^D10A^-mRNA with respect to targeting *MYCN* (1.7 to 2.6-fold) or *ALK* (1.7 to 2.1-fold) individually (Fig. 5D). Notably, the observed fold increase in Cas9^D10A^-mediated cell-killing efficacy when targeting both gene amplifications in IMR-32 cells is consistent with the approximate doubling of sites targetable by Cas9^D10A^. We did not observe any substantial increase in toxicity when targeting *MYCN* or *ALK* in combination with *AAVS1* in IMR-32 cells (Fig. 5E), or when targeting *MYCN* and *ALK* simultaneously in SH-SY5Y cells (Fig. 5F). These observations provide further evidence that the toxicity conferred by Cas9^D10A^ is dependent on the number of targetable sites in the genome.

### Cas9^D10A^ demonstrates comparable cell killing efficacy to Cas9 nuclease with better selectivity

As noted previously, observations of Cas9 nuclease (Cas9^WT^)-induced cellular toxicity during large-scale loss of function screens^26–28^ proved foundational for the development of some Cas9^WT^-based cancer cell-killing technologies^29,30^. Unlike Cas9^D10A^, however, DSBs generated by Cas9^WT^are not replication dependent, and the repair of Cas9^WT^ DSBs is considerably more mutagenic^19,23,29,30^. Consequently, we speculate that a cancer therapeutic based on Cas9^WT^ could encounter potential problems including: 1) target site depletion by means of sequence alteration due to imprecise DSB repair^116–118^; and 2) DSB-related toxicity in post-mitotic^119,120^ or labile cell types, such as hematopoietic stem and progenitor cells (HSPCs)^121^.

In theory, employing Cas9^D10A^ instead of Cas9^WT^ would circumvent some of these issues. As such, we sought to directly compare Cas9^D10A^ and Cas9^WT^ with regards to selective cell killing. First, we validated Cas9^WT^-mediated cell-killing in *MYCN*-amplified, SK-N-BE(2)C cells and assessed its efficacy when delivered as mRNA at the same concentration (30 nM) as was previously employed for Cas9^D10A^ (Supplementary Fig. 23). As anticipated, Cas9^WT^ displayed efficacious cell-killing when targeting either *LINE-1* or *MYCN*. Surprisingly, however, Cas9^WT^ induced an appreciable degree of toxicity (0.5-fold) when targeting *AAVS1*, a safe-harbor locus, an outcome not observed when employing Cas9^D10A^ (Supplementary Fig. 23A). Upon further investigation, SK-N-BE(2)C cells targeted at *AAVS1* with Cas9^WT^ demonstrated an ~2-fold median enrichment in DNA damage, as would be expected of a non-amplified locus, but an ~105-fold mean enrichment in DNA damage potentially as a result of off-target activity in a subset of cells within the population that received higher levels of Cas9^WT^ mRNA (Supplementary Fig. 23B, C).

Next, we assessed SK-N-BE(2)C cells for sequence alterations at the *MYCN* target site post-treatment with either Cas9^WT^ or Cas9^D10A^. Target site variations were assessed by amplicon-sequencing using genomic DNA isolated from surviving SK-N-BE(2)C cells at 3 days post-treatment with Cas9^WT^ or Cas9^D10A^. Amplicon-sequencing analyses revealed ~84% of target sequences were modified in SK-N-BE(2)C cells treated with Cas9^WT^ (Supplementary Fig. 18A), whereas only ~3.7% were modified when treated with Cas9^D10A^ (Supplementary Fig. 24B). Moreover, sequences modified by Cas9^WT^-mediated DSBs demonstrate mixed imprecise repair outcomes with the majority being a single-nucleotide insertion, consistent with NHEJ (Supplementary Fig. 24C). In contrast, sequences modified by Cas9^D10A^, though present in low abundance, predominantly exhibit deletions, consistent with exonuclease-mediated end-resection and repair during S-phase of the cell cycle (Supplementary Fig. 24D).

Given the ostensible restriction of Cas9^D10A^-induced toxicity to proliferating cells, we sought to validate this notion by assessing cytotoxic effects in post-mitotic cells. We chose to target *LINE-1* as it provides a challenging test given the large number of sites present within the genome. For a post-mitotic cell system, we induced neuronal differentiation in SK-N-BE(2)C cells expressing *LINE-1* or *AAVS1* targeting sgRNA. Neuronal SK-N-BE(2)C cells were transfected with either Cas9^WT^ or Cas9^D10A^-mRNA and assessed for changes in confluency with crystal violet stain at 3 days post-treatment relative to an untreated control as a measure of cellular toxicity (Supplementary Fig. 25A, B). Cas9^WT^ proved highly toxic when targeting *LINE-1*, and to our surprise, it was also toxic at *AAVS1* in neuronal cells, suggesting a high level of mRNA delivery with substantial off-target activity. In contrast, no significant toxicity (*P* » 0.05) was observed when either site was targeted with Cas9^D10A^ at the same mRNA dose.

Lastly, we sought to assess unwanted activity in HSPCs, as they are labile cells where levels of N-MYC and c-MYC are critical for stem cell maintenance and proper hematopoietic differentiation^122^. Moreover, the damage and depletion of HSPCs is an unfortunate consequence of more conventional chemotherapies^4^. Here, primary human cord blood CD34+ cells were electroporated with Cas9^WT^-mRNA (30 nM) and sgMYCN-1 (50 µM) or sgAAVS1 (50 µM), Cas9^D10A^-mRNA (30 nM) and sgMYCN-1 (50 µM), GFP-mRNA only (30 nM) or sgRNA only (50 µM) and assessed for changes in cell viability at 3 days post-treatment relative to a mock control (Supplementary Fig. 25C). Modest toxicity was observed in Cas9^WT^-treated HSPCs when targeting *MYCN* (0.7-fold) or *AAVS1* (0.8-fold), whereas Cas9^D10A^-treated HSPCs maintained a similar level of viability comparable to other controls.

Next, we assessed target site mutagenesis in surviving HSPCs. Genomic DNA was isolated at 3 days post-treatment with Cas9^WT^ or Cas9^D10A^ and assessed for editing at the sgMYCN-1 target site. Despite the appreciable level of activity at the sgAAVS1 target site, Cas9^WT^ demonstrated modest editing at the sgMYCN-1 target site as assessed by Sanger sequencing (Supplementary Fig. 25D). An assessment of Cas9^WT^-mediated editing at the *MYCN* and *AAVS1* loci in *MYCN* non-amplified SH-SY5Y resulted in similar editing activity and viability as observed in HSPCs (Supplementary Fig. 25E). Importantly, Cas9^D10A^ editing levels at the *MYCN* locus in HSPCs were below the level of detection by Sanger sequencing. These observations suggest that Cas9^D10A^ editing activity at the *MYCN* locus in HSPCs is minimal.

### Cas9^D10A^ eliminates other cancer cell types in a gene amplification dependent manner

**Fig. 6:**
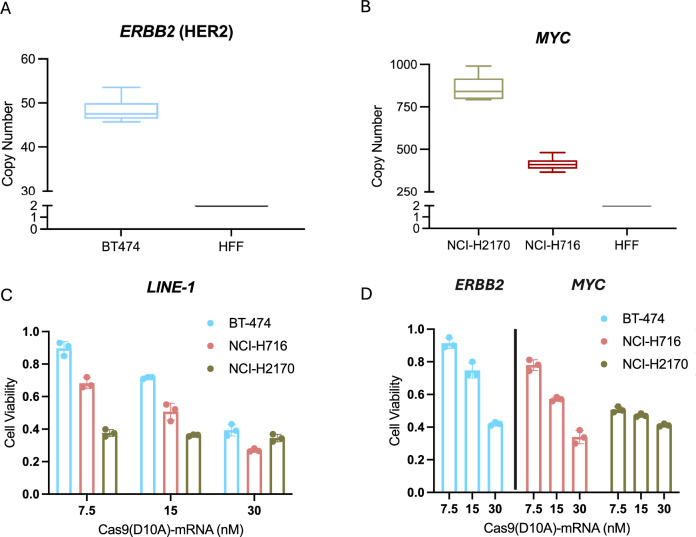
Cas9^D10A^ demonstrates efficacy in *ERBB2* (HER2)-amplified breast cancer, *MYC*-amplified non-small cell lung cancer, and *MYC*-amplified colorectal cancer cells. **A**, **B** qPCR analysis of gene copy number variance in (**A**) *ERBB2* (HER2)-amplified breast cancer cell line, BT-474 and (**B**) *MYC*-amplified non-small-cell lung cancer and *MYC*-amplified colorectal cancer cell lines, NCI-H2170 and NCI-H716, respectively (*n* = 3 biological replicates with technical duplicates). Data are presented normalized to non-amplified human foreskin fibroblast (HFF) where for each box plot the horizontal line represents the median, the top and bottom of the box represent the upper and lower quartiles, respectively, and the top and bottom whiskers represent the maximum and minimum values, respectively. **C**, **D** Targeting of (**C**) *LINE-1* or (**D**) *ERBB2* (HER2) locus in BT-474 or *MYC* locus in NCI-H716 or NCI-H2170 cells demonstrates a cytotoxic effect similar to that observed when targeting the *MYCN* locus in *MYCN*-amplified neuroblastoma cells. All cells were assessed for changes in cell viability at 3 days post-treatment with Cas9^D10A^-mRNA (*n* = 3 biological replicates). Data are presented as mean ± s.d. and normalized relative to viability of cells expressing *AAVS1* targeting sgRNA treated with Cas9^D10A^. Source data are provided as a Source Data file.

Given the success of Cas9^D10A^ in the selective depletion of *MYCN*-amplified neuroblastoma cells, we asked whether our observations would translate to other cancer types exhibiting gene amplification. Consequently, we targeted *ERBB2* (HER2), which is amplified ( ~ 50 copies) in the breast cancer cell line BT-474, and *MYC*, which is amplified ( > 400 copies as ecDNA)^123,124^ in non-small cell lung cancer cell line NCI-H2170 and the colorectal cancer cell line NCI-H716 (Fig. 6A, B). Each cell line was modified to express an sgRNA targeting a non-coding sequence downstream of the annotated coding sequence of the amplified gene, *LINE-1* or *AAVS1*. Cellular toxicity was observed when targeting *LINE-1* with Cas9^D10A^ in each cell line, whereas targeting *AAVS1* with Cas9^D10A^ had minimal effect on cell viability or proliferation (Fig. 6C and Supplementary Fig. 15). Moreover, cellular toxicity was observed in BT-474, NCI-H716, and NCI-H2170 cells when targeting gene amplified loci (*ERBB2* (HER2) or *MYC*) with Cas9^D10A^ consistent with observations in the *MYCN*-amplified cell lines (Fig. 6D). Cas9^D10A^-mediated cell killing was dose responsive in BT-474 and NCI-H716 cell lines when targeting amplified loci. However, NCI-H2170 cells demonstrated high sensitivity to Cas9^D10A^ at all concentrations when targeting amplified loci (Fig. 6C, D).

Next, we evaluated Cas9^D10A^-mediated cell killing in the presence of a CHK1 or ATR inhibitor (Supplementary Fig. 26). Similar to our observations in the *MYCN*-amplified neuroblastoma cell lines, delivery of Cas9^D10A^ targeting an amplified loci in combination with CHK1i appreciably improved the cell-killing efficacy of Cas9^D10A^ at lower concentrations for BT474 and NCI-H716 cells (Supplementary Fig. 26A–C) whereas delivery of Cas9^D10A^ in combination with ATRi was more variable in its effect on cell killing (Supplementary Fig. 26D–F).

Finally, we assessed whether the cellular toxicity of Cas9^D10A^-induced DNA damage could be attenuated in the same manner as the *MYCN*-amplified neuroblastoma cell lines by treating BT-474, NCI-H716, or NCI-H2170 cells with either rucaparib (PARPi) or calpastatin (CAST) in the presence of Cas9^D10A^ (Supplementary Fig. 27). Consistent with our observations in *MYCN*-amplified neuroblastoma cells, the cytotoxic effects of Cas9^D10A^ targeting an amplified locus were significantly reduced at many doses in the presence of PARP inhibitors (Supplementary Fig. 27A–C) or calpastatin (Supplementary Fig. 27D–F)*.* Overall, our observations suggest a conserved mechanism of amplification-dependent Cas9^D10A^-mediated cell-killing across four distinct cancer types harboring four distinct oncogene amplifications.

## Discussion

Here we have demonstrated that CRISPR-Cas9 nickases can be employed for the selective depletion of cancer cells by targeting disease-specific gene amplifications. Gene amplification is associated with >50% of common cancer types and is more prevalent in recurrent or metastatic cancers^125^ suggesting that Cas9 nickase could have broad utility. A previous report has leveraged Cas9 nucleases to promote selective cancer cell death by generating DSBs in sequence-divergent, repetitive elements within the non-coding genome as a potential therapeutic approach for cancer^30^. However, targeting extensively amplified loci using Cas9^D10A^ affords an opportunity to exploit this genomic vulnerability using reagents that do not require optimization to patient-specific sequence variations. In our survey of the *MYCN* locus, the position of the Cas9^D10A^ target sequence did not appreciably influence the degree of toxicity conferred by the nickase suggesting a vast array of target sites can be utilized provided that they are amplified. This attribute of Cas9 nickase-induced toxicity in the context of high-copy number genomic sequences has important implications for the development of reagents tailored to specific cancer types that avoids potential damage to functional sequence elements within the genome of normal cells, thereby minimizing collateral damage and associated systemic toxicity.

One critical advantage of nickase-mediated cell killing over nuclease-based systems is the low indel rates induced at the target site by the nickase^45^. The low indel frequencies associated with a nickase minimize the chance of unintended sequence alterations at the target locus in normal cells (e.g. *MYCN* in HSPCs). Avoiding the use of a nuclease also minimizes the occurrence of other unwanted DNA repair outcomes, such as large deletions at the target site, that are associated with DSB repair in normal cells^23,126^. Low indel frequencies at the Cas9^D10A^ target site within a cancer cell harboring a gene amplification minimizes the opportunity for resistance by means of target sequence-alteration, a pitfall that has been observed with nuclease-based systems for some therapeutic applications^116–118^. In addition, Cas9^D10A^ can be used for multiplex targeting in instances of dual gene amplification (*MYCN* and *ALK* in IMR-32 cells), demonstrating the ability to harness multiple guides to increase the potency of the Cas9^D10A^ system.

Our working model for cellular toxicity mediated by Cas9^D10A^ nickase treatment is the replication-dependent formation of DSBs^34^ followed by the aberrant entry of cells into mitosis with extensive, unrepaired DNA damage. As it stands, there are many other genetic alterations (*TP53* status, DNA repair factor inactivation, etc.) that may influence the efficiency of Cas9^D10A^-mediated cell-killing. Our observations suggest that the toxicity conferred by Cas9^D10A^ is unlikely to be caused by changes in target locus copy number or gene expression level, as the observed changes for *MYCN* were relatively modest in Cas9^D10A^-treated *MYCN*-amplified neuroblastoma cell lines relative to the distribution of *MYCN*-amplification levels across different neuroblastoma cell lines tested in our experiments. These results suggest that the mechanism underlying Cas9^D10A^-mediated cell-killing is independent of gene function. However, the dependence of Cas9 nickase toxicity on DNA replication is a potential limitation for this approach, as quiescent cancer cells will not be subjected to the generation of DSBs. However, this challenge is also faced by other effective chemotherapies, such as topotecan and etoposide, where genotoxicity is associated with DNA replication^127^.

Furthermore, we have demonstrated that Cas9^D10A^ can be used in combination with existing cytotoxic agents, such as CHK1 inhibitors, to augment the potency of Cas9^D10A^-mediated cell-killing. Thus, Cas9^D10A^ treatment could potentially serve as a means of increasing the sensitivity of cancer cells harboring gene amplifications to a variety of conventional therapeutics^128–130^. Surprisingly, ATR inhibition was less effective than CHK1 inhibition when used in combination with Cas9^D10A^ to augment cell-killing. In some cell lines, ATR inhibition was modestly protective when paired with a high concentration of Cas9^D10A^. These observations suggest that stimulation of the ATR-mediated DDR pathway could play a positive or negative role in Cas9^D10A^-mediated cell-killing depending on the cellular environment. For example, NCI-H2170 cells harbor a missense mutation (p.R2190C) within ATR, located between the FAT (FRAP-ATM-TRRAP) and kinase domains, which may be responsible for the attenuated response to ATR and CHK1 inhibitors^131,132^. As such, more comprehensive analyses of the DDR and cell death pathway(s) elicited by targeting Cas9^D10A^ to high copy number sites may provide insights into additional cell-signaling pathway perturbations for augmenting nickase-induced cellular toxicity.

Finally, despite the favorable activity observed when applying Cas9D10A—mediated cell-killing in vitro, its utility for therapeutic application is contingent on the feasibility of delivery to the tumor in vivo. As such, directing future efforts into the development and optimization of a suitable carrier system is paramount for the therapeutic functionalization of this promising Cas9 nickase–based approach.

## Methods

### Ethics statement

Human Umbilical Cord Blood (UCB) used to isolate CD34+ HSPCs was obtained in accordance with the Committee for the Protection of Human Subjects in Research guidelines of the University of Massachusetts Chan Medical School. UCB was provided by the medical staff of the University of Massachusetts Memorial Umbilical Cord Blood Donation Program. The UBC core does the verbal consenting. The UCB are provided to us a deidentified specimens from the UCB Core at UMass: https://www.umassmed.edu/research/cores/cord-blood/. Consequently, sex and/or gender were not considered in the study design.

### Mammalian cell culture

SK-N-BE(2)C [CRL-2268], CHP-212 [CRL-2273], IMR-32 [CCL-127], SH-SY5Y [CRL-2266], BT-474 [HBT-20], NCI-H2170 [CRL-5928], NCI-H716 [CCL-251], and HEK293T [CRL-3216] cells were sourced through the American Type Culture Collection (ATCC). KELLY [ACC 355] and NGP [ACC 676] cells were sourced through the Leibniz Institute (DSMZ). All mammalian cell cultures were maintained at 37 °C, 5% CO_2_. SK-N-BE(2)C, NGP, CHP-212, IMR-32 and SH-SY5Y neuroblastoma cell lines were grown and maintained in Dulbecco’s Modified Eagle Medium/Nutrient Mixture F-12, GlutaMAX™ supplement (DMEM/F12, GlutaMAX™ supplement; Gibco, #10565018), supplemented with 10% fetal bovine serum (FBS; R&D Systems, #S12450H) and 1% penicillin-streptomycin (PS; 10,000 U/mL; Gibco, #15140122). KELLY neuroblastoma cells were grown and maintained in RPMI 1640, 1X with L-glutamine (Corning, #10-040-CV), supplemented with 15% FBS (R&D Systems, #S12450H) and 1% PS (10,000 U/mL; Gibco, #15140122). BT-474 breast cancer cells were grown and maintained in RPMI 1640, 1X with L-glutamine (Corning, #10-040-CV), supplemented with 10% FBS (R&D Systems, #S12450H) and 1% PS (10,000 U/mL; Gibco, #15140122). HEK293T human embryonic kidney cells were grown and maintained in Dulbecco’s Modified Eagle Medium (DMEM; Gibco, #11965092) supplemented with 10% FBS (R&D Systems, #S12450H) and 1% PS (10,000 U/mL; Gibco, #15140122). NCI-H2170 non-small cell lung cancer cells and NCI-H716 colorectal cancer cells were grown and maintained in RPMI 1640, 1X with L-glutamine (Corning, #10-040-CV), supplemented with 10% FBS (R&D Systems, #S12450H) and 1% PS (10,000 U/mL; Gibco, #15140122). All sgRNA expression cell lines were grown and maintained in the same media as previously described, further supplemented with Geneticin™ (1 mg/mL) (G418 Sulfate; 50 mg/mL; Gibco, #10131027). HEK293T sgRNA expression cell lines and SK-N-BE(2)C RPA overexpressing cell lines were grown and maintained in the same media as previously described, further supplemented with blasticidin S HCl (10 µg/mL) (Gibco, #A1113903). Primary CD34+ hematopoietic stem and progenitor cells (HSPCs) isolated from human cord blood were obtained as gift from Dr. Michael Brehm (University of Massachusetts Chan Medical School). HSPCs were maintained in StemPro®—34 SFM Complete Medium (Gibco, #10639-011) supplemented with L-glutamine (2 mM; Gibco, #21051-024), and cytokines SCF (MedChemExpress, #HY-P70781), IL-3 (MedChemExpress, #HY-P70576), and GM-CSF (MedChemExpress, #HY-P7016A\CS-P7016A) according to the manufacturer’s instructions. All cell lines were grown and maintained in 25 cm^2^ (Celltreat, #229331) or 75 cm^2^ (Celltreat, #229341) tissue culture flasks. All cell lines were tested for mycoplasma contamination at regular intervals using the e-Myco^TM^ VALiD mycoplasma PCR detection kit (LiliF Diagnostics, #25239).

### Expression vectors

Cas9 nickase in vitro transcription vectors contain T7 RNA polymerase promoter for the production of synthetic Cas9^D10A^ or Cas9^H840A^ mRNA. Additional elements include a 5’UTR derived from human hemoglobin alpha (hHBα), Kozak sequence, and 3’UTR derived from amino-terminal enhancer of split (AES) and mitochondrially encoded 12S rRNA (mt-RNR1) motifs^133–135^, and poly-A_150_. Additionally, all coding sequences were codon optimized to deplete the corresponding mRNA transcript of any unnecessary uridine bases to reduce the stimulation of innate immune sensors, with all necessary uridine bases being replaced with the modified nucleobase N1-methylpseudouridine (m1Ψ) during mRNA production^136^.

Guide RNA expression donor vectors contained a G418 sulfate selection marker (NeoR/KanR), or a blasticidin selection marker under the transcriptional control of a murine phosphoglycerate kinase (mPGK) promoter, and the desired sgRNA sequence under the transcriptional control of a U6 promoter. The expression cassette was placed between piggyBac left and right inverted terminal repeats (ITRs) to facilitate integration by piggyBac transposition^137^. The RPA1, RPA2, and RPA3 expression donor vector was derived from p11d-tRPA(123) (Addgene, #102613; a gift from Marc Wold)^138^ and placed into a piggyBac donor backbone containing a blasticidin selection marker under the transcriptional control of an EF1α promoter. RPA was expressed under the transcriptional control of a CAG promoter and separated by 2 A self-cleavable peptide linkers^139^, RPA1-T2A-RPA2-P2A-RPA3. Expression of the RPA transgene was verified by Western blot.

### Cloning of sgRNAs

Oligonucleotides to construct sgRNA inserts were ordered from GENEWIZ (Azenta Life Sciences, Inc., USA). Vector backbone was digested overnight with BbsI-HF (New England Biolabs, #R539L) and supplemented with Quick CIP (New England Biolabs, #M0525L) to prevent recircularization. Forward and reverse oligonucleotides corresponding to each sgRNA were resuspended to a concentration of 20 µM then mixed; 5 µL forward oligo, 5 µL reverse oligo, 5 µL T4 polynucleotide kinase reaction buffer (10x; New England Biolabs, #B0201S), and 35 µL ddH_2_O. Mixture was incubated at 95 °C for 4 min and left to cool to room temperature. After cooling, ATP (10 mM) and T4 polynucleotide kinase (T4 PNK, New England Biolabs, #M0201L) were added directly to the mixture and incubated at 37 °C for 30 min. Annealed oligos (2 µL) and digested backbone (20 ng) were then ligated overnight at 16 °C with T4 DNA ligase, and used to transform NEB Stable (New England Biolabs, #C3040) cells under ampicillin selection (Thermo Scientific, #J63807-06). Successful insertion of sgRNA sequences verified by Sanger sequencing at GENEWIZ (Azenta Life Sciences, Inc., USA).

### Stable transfection for the production of sgRNA and RPA(123) expressing cell lines

Neuroblastoma cell lines, BT-474 breast cancer cells, NCI-H716 colorectal cancer cells or NCI-H2170 non-small cell lung cancer cells ( ~ 2 × 10^6^) were transfected with 0.5 µg of Super PiggyBac transposase expression vector (System Biosciences, #PB210PA-1) and 2 µg of sgRNA donor vector corresponding to either sgLINE-1 (5’ – ATTCTACCAGAGGTACAAGG – 3’); sgMYCN-1 (5’ – CAATGGAGACCCCATATGGG – 3’); sgMYCN-2 (5’ – CAGCTCCGAGCCCCCGAGCT – 3’); sgMYCN-3 (5’ – GGACCCAGGGCTGCGTTCTT – 3’); sgMYCN-4 (5’ – CACCAGTGTGGGGTTCTGCT – 3’); sgHER2-1 (5’ – GAGATCGAGTCGCGCCTCGG – 3’); sgMYCC (5’ – GATTCCTACCGTCGTCTGAG – 3’); sgALK (5’ – ACAGGCCGCTGGTGGTCCTA – 3’); or sgAAVS1 (5’ – GTCCCCTCCACCCCACAGTG – 3’) using Lipofectamine 3000 (Invitrogen, #L3000001) and incubated for 6 h before replacing the media. Cells were allowed to recover for 3 days prior to initiating antibiotic selection with either Geneticin™ (1 mg/mL) (G418 Sulfate; 50 mg/mL; Gibco, #10131027), blasticidin S HCl (10 µg/mL) (Gibco, #A1113903), or both when two differentially marked donor vectors were used to select for the integration of two different sgRNA expression cassettes. Cells were cultured under antibiotic selection for 3–5 days and then allowed to recover for 3 days without antibiotic selection to facilitate outgrowth and dilution of unincorporated donor vector prior to initiating an additional round antibiotic selection. The method used for the generation of SK-N-BE(2)C-RPA(123) is identical to that described for the sgRNA transgenes but employed an alternate selection marker to allow the selection of cells harboring both sgRNA and RPA(123) expression cassettes.

### in vitro transcription (IVT)

Cas9^D10A^, Cas9^H840A^, or Cas9^WT^ IVT vectors were constructed and subsequently digested overnight with Esp3I (New England Biolabs, #R0734L) to ensure complete linearization of the DNA template. Linearized template was subsequently purified using a DNA clean & concentrator – 5 spin-column (Zymo Research, #D4013) and eluted with nuclease-free H_2_O (NF- H_2_O). Individual IVT reactions (20 µL) were carried out with 1 µg of linearized template using the HiScribe T7 High Yield RNA Synthesis Kit (New England Biolabs, #E2040S), exchanging UTP with N1-methylpseudouridine-5’-triphosphate (100 mM; TriLink, #N-1081-5) and further supplementing the reaction with CleanCap Reagent AG (3’ OMe) (4 mM; TriLink, #N-7413), RNasin Plus ribonuclease inhibitor (40 U; Promega, #N2615), and yeast inorganic pyrophosphatase (New England Biolabs, #M2403S). Reaction mixture was incubated at 37 °C for 2 h before increasing the reaction volume to 50 µL with nuclease free—H_2_O and treating the mixture with DNase I (New England Biolabs, #M0303L) at 37 °C for 15 min to remove residual DNA template. The final IVT product was purified using the Monarch RNA Clean-up Kit (500 µg; New England Biolabs, #T2057L) according to the manufacturer’s instructions. Typical yields obtained from the method described were in excess of 100 µg per reaction, as quantified by a NanoDrop UV/Vis spectrophotometer (Thermo Scientific, #ND-ONE-W). IVT products were assessed by denaturing 1 µg of IVT-produced mRNA in RNA loading dye (2X; New England Biolabs, #B0363S) for 10 min at 70 °C and running the product on a non-denaturing agarose gel (1%). Verified mRNA product was subsequently aliquoted at a concentration of 1 µg/µl and stored at −80 °C for downstream applications.

### Electroporation of cells for the delivery of Cas9^D10A^, Cas9^H840A^, or Cas9^WT^ mRNAs in vitro

Cas9^D10A^, Cas9^H840A^, or Cas9^WT^ mRNAs were delivered by electroporation to each of the cell lines using the Neon™ Transfection System 10 µL kit (Invitrogen, #MPK1096) or Neon™ NxT Electroporation System 10 µL kit (Invitrogen, #N1096). Electroporation parameters were determined empirically for the following cell lines; SK-N-BE(2)C, IMR-32, and SH-SY5Y were electroporated with a 1300 V pulse voltage, 20 ms pulse width, 3 pulses; KELLY, NGP, and CHP-212 with a 1650 V pulse voltage, 10 ms pulse width, 3 pulses; HEK293T cells with a 1200 V pulse voltage, 20 ms pulse width, 2 pulses; and BT-474, NCI-H716, and NCI-H2170 cells with a 1450 V pulse voltage, 20 ms pulse width, 2 pulses. CD34+ HSPCs were electroporated a 1600 V pulse voltage, 10 ms pulse width, and 3 pulses according to the manufacturer’s instructions. All non-primary cells (2 × 10^5^ per condition) were early to mid-passage ranging from passage number 5–10. Primary HSPCs were cultured for 24 h after thawing prior to electroporation. All electroporated cells demonstrated >90% viability at the time of electroporation as determined by trypan blue exclusion (Gibco, #15250061) using a TC20 automated cell counter (BioRad, #1450102). Cas9 nickase or Cas9 nuclease mRNA was prepared in resuspension buffer R supplemented with RNasin Plus (40 U, Promega, #N2615) at the desired concentration in which to resuspend the cells for electroporation. Co-delivery of Cas9^D10A^ mRNA (30 nM) and synthetic sgRNA (300 pmol) (Integrated DNA Technologies, Inc., USA) was carried out in the same manner as previously described.

### Cell viability and proliferation assays

Changes in cell viability and cytotoxicity were assessed using the RealTime-Glo™ MT Cell Viability Assay (Promega, #G9713), a nonlytic ATP-independent bioluminescent assay that measures the reduction potential of cells. Given the semi-adherent nature of many of the neuroblastoma cell lines, minimal manipulation of cells post-electroporation was critical. Cell viability was determined in an end-point manner at 3 days post-treatment with either Cas9^D10A^, Cas9^H840A^, or Cas9^WT^ mRNAs by measuring the relative luminescence units (RLUs) using a GloMax® Explorer Multimode Microplate Reader (Promega, #GM3500). Changes in cell viability were normalized to *AAVS1* targeted cells as no significant difference in viability was observed between *AAVS1* and mock/untreated controls (Supplementary Fig. 15). Cell proliferation was monitored at 1-, 2-, and 3 days post-treatment with Cas9 nickase mRNA or by QIBC using the Celigo Image Cytometer (Nexcelcom Bioscience, USA) to measure the cell population following staining cell with Hoecsht 33342 (1 µg/mL; Invitrogen, #H1399) at 37 °C for 30 min. Where applicable, cells were supplemented with various reagents: in the presence of MK8776 (MedChemExpress, #HY-15532), rucaparib (MedChemExpress, #HY-10617A), PDD00017273 (MedChemExpress, # HY-108360), berzosertib (Selleck, #S7102), calpastatin (Millipore Sigma, #208902), alisertib (MedChemExpress, #HY-10971), etoposide (MedChemExpress, #HY-13629R), or topotecan (MedChemExpress, #HY-13768A) at concentrations ≤IC50 to prevent confounding toxicity.

### Comet assay

Single cell gel electrophoresis was carried out on SK-N-BE(2)C, KELLY, NGP, CHP-212, SH-SY5Y, and HEK293T cells expressing different sgRNAs 3 days post-electroporation with Cas9^D10A^ (30 nM) or Cas9^H840A^ (30 nM) using the Comet Assay Electrophoresis Kit (R&D Systems, #4250-050-ESK) according to the manufacturer’s instructions. Cells were stained with Hoecsht 33342 (1 µg/mL; Invitrogen, #H1399) for 1 h at room temperature. Cells were visualized and imaged using the EVOS™ FL Imaging System (Invitrogen, #AMF4300, LED, Ex: 357/44 Em: 447/60, 4/10 PH, Sony ICX285AQ color CCD, 2/3 in 1360 × 1024, 1.4 Megapixels). Cells used for analysis were imaged at 10X magnification, representative cell images taken at 20X magnification. Tail moments were quantified using an ImageJ (NIH) Comet Assay macro (developed by Herbert M. Geller, NIH, 1997, and further modified by Robert Bagnell, 2011, Department of Pathology and Laboratory Medicine, UNC-CH).

### Cell cycle analysis

SK-N-BE(2)C cells (2 × 10^5^ cells per condition) expressing the *LINE-1*, *MYCN* or *AAVS1* sgRNA were electroporated with Cas9^D10A^ mRNA (30 nM) and collected at 1-, 2-, and 3 days post-treatment to assess changes in cell cycle progression. Harvested cells were fixed in 95% ethanol overnight at 4 °C, washed twice with PBS (1X), and resuspended in propidium iodide (Thermo Scientific, #J66764-MC) and RNase A (Invitrogen, #AM2271) solution. Cells were stained at 37 °C for 30 min and subsequently analyzed using the MACSQuant® Analyzer 10 Flow Cytometer (Miltenyi Biotec), and the resulting cell populations were characterized using FlowJo analysis software.

### Metabolism assays

SK-N-BE(2)C, NGP, and SH-SY5Y cells (2 × 10^5^) expressing *LINE-1*, *MYCN* or *AAVS1* targeting sgRNA were electroporated with Cas9^D10A^ mRNA (30 nM) and monitored for changes in intracellular calcium flux at 1-, 2-, and 3 days post treatment using the calcium indicator dye, Fluo-4 AM (Invitrogen, #F14201). Fluo-4 AM was reconstituted in DMSO and added to cells at a final concentration of 3 µM in PBS (1X) and incubated at room temperature for 1 h prior to analysis. ROS accumulation was monitored at 1-, 2-, and 3 days post treatment using the Total Reactive Oxygen Species (ROS) Assay Kit, 520 nm (Invitrogen, #88-5930-74) according to the manufacturer’s instructions. Mean integrated fluorescence intensities were acquired by QIBC using the Celigo Image Cytometer (Nexcelcom Bioscience, USA). Changes in total cellular ATP were assessed at 3 days post-treatment with Cas9^D10A^ using CellTiter-Glo® Luminescent Cell Viability Assay (Promega, #G7572) according to the manufacturer’s instructions. Changes in NAD+ were assessed at 3 days post-treatment with Cas9^D10A^ using NAD/NADH-Glo™ (Promega, #G9072). Changes in ATP or NAD+ were determined by measuring the relative luminescence units (RLUs) using a GloMax® Explorer Multimode Microplate Reader (Promega, #GM3500).

### Western blotting

SK-N-BE(2)C, NGP, and SH-SY5Y cells ( ~ 2 × 10^6^) expressing the desired sgRNA were electroporated with Cas9^D10A^ mRNA (30 nM) for protein extraction 3 days post-treatment. Cells washed twice with ice-cold PBS (1X) and lysed with RIPA buffer (Thermo Scientific, #89900) containing Halt™ Protease and Phosphatase Inhibitor (Thermo Scientific, #78440) on ice for 30 min. Lysates were centrifuged at >15,000 g for 30 min at 4 °C to pellet cell debris and collect the supernatant. Protein concentration was measured by bicinchoninic acid (BCA) assay (Thermo Scientific, #23225). SDS-PAGE gel electrophoresis was carried out using 10 – 20% tricine gels (Invitrogen, #EC6625BOX) in tricine SDS running buffer (1X; Invitrogen, #LC1675) at 130 V constant voltage for 1.5 h at room temperature. Gels were transferred to PVDF membranes using the iBlot® Dry Blotting System, and subsequently blocked for 1 h at room temperature in SuperBlock™ Blocking Buffer (0.05% Tween-20; Thermo Scientific, #37535) before overnight primary antibody incubation at 4 °C.

Primary antibodies used include; N-Myc monoclonal antibody (NCM-II 100; 1:500, Invitrogen, #MA1-170); PARP1 polyclonal antibody (1:1000, Invitrogen, #PA5-34803); Poly ADP-ribose antibody, clone 10H (1:1000, Millipore Sigma, #MABC547); Caspase 3 polyclonal antibody (1:1000, Invitrogen, #PA5-77887); Mu-Calpain polyclonal antibody (1:1000, Invitrogen, #PA5-17547); Calpain 2 polyclonal antibody (1:1000, Invitrogen, #PA5-17494); phospho-EXO1(Ser746) (1:1000, Millipore Sigma, #ABE1066); anti-gamma-H2AX polyclonal antibody (1:10,000, Bethyl Laboratories, #A300-081A); phospho-CHK1 (Ser345) polyclonal antibody (1:1000, Invitrogen, #PA5-34625); phospho-RPA32 (Ser33) polyclonal antibody (1:1000, Invitrogen, #PA5-39809); RPA2 monoclonal antibody (9H8) (1:500, Invitrogen, #MA1-26418); β-actin (8H10D10) mouse monoclonal antibody (1:2000, Cell Signaling Technology, #3700). Blots were washed 3–5 times in TBST prior to secondary antibody incubation for 1.5 h at room temperature with either anti-rabbit IgG, HRP-conjugated antibody (1:3000, Cell Signaling Technology, #7074) or anti-mouse IgG, HRP-conjugated antibody (1:3000, Cell Signaling Technology, #7076). Blots were incubated in ECL substrate (Thermo Scientific, #32106) for 1–2 min before visualization using ChemiDoc™ Touch Imaging System (BioRad). Uncropped blots are present in the Source Data file.

### Assessing apoptotic and necrotic cell death activity in Cas9^D10A^—treated neuroblastoma cells

Cleavage of caspase 3 was assessed in NGP and SH-SY5Y *LINE-1*, *MYCN*, or *AAVS1* sgRNA expressing cells by Western blot at 3 days post-treatment with Cas9^D10A^ (*See Western blotting section*). Staurosporine (1 µM; MedChemExpress, #HY-15141) was used as a positive control for caspase 3 activation in SH-SY5Y cells and harvested for protein extraction following 3-h of incubation under normal culture conditions (*See mammalian cell culture section)*. SK-N-BE(2)C and NGP *LINE-1*, *MYCN*, or *AAVS1* sgRNA expressing cells ( ~ 2 × 10^5^) were electroporated with Cas9^D10A^ – mRNA (30 nM) and incubated with Z-DEVD-FMK (18 µM; MedChemExpress, # HY-12466) or PFT-α (20 µM; Millipore Sigma, #P4359). Changes in cell viability and cytotoxicity were assessed at 3 days post-treatment using the RealTime-Glo™ MT Cell Viability Assay (Promega, #G9713). Calpain autoproteolytic cleavage was assessed in SK-N-BE(2)C *LINE-1*, *MYCN*, or *AAVS1* sgRNA expressing cells at 3 days post-treatment with Cas9^D10A^ (*See Western blotting section*). Calpain activity was assessed in both SK-N-BE(2)C and NGP *LINE-1*, *MYCN*, or *AAVS1* sgRNA expressing cells ( ~ 2 × 10^5^) electroporated with Cas9^D10A^ – mRNA (30 nM). Calpain activity was measured at 3 days post-treatment with Cas9^D10A^ using the Calpain-Glo™ Protease Assay (Promega, #G8501) according to the manufacturer’s instructions. Induction of calpain activation was achieved following 30 min of incubation in growth media supplemented with CaCl_2_ (2 mM). Lactate dehydrogenase activity was assessed in both SK-N-BE(2)C and NGP *LINE-1*, *MYCN*, or *AAVS1* sgRNA expressing cells ( ~ 2 × 10^5^) electroporated with Cas9^D10A^ – mRNA (30 nM). Lactate dehydrogenase activity was assessed at 3-days post-treatment with Cas9^D10A^ using the CyQUANT™ LDH Cytotoxicity Assay Kit (Invitrogen, #C20301) according to the manufacturer’s instructions.

### Assessment of neuronal differentiation by immunocytochemistry post treatment with Cas9^D10A^

Surviving SK-N-BE(2)C cells expressing the designed sgRNAs electroporated with Cas9^D10A^ – mRNA (30 nM) or incubated with ATRA (10 µM; MedChemExpress, # HY-14649) were assessed at 5 days post-treatment. Cells were fixed with ice-cold methanol (100%) for 15 min at 4 °C, washed briefly with PBS (1X), and permeabilized with PBST (0.5%) for 5 min at room temperature. Following permeabilization, cells were washed briefly with PBS (1X) and blocked with SuperBlock™ Blocking Buffer (0.05% Tween-20; Thermo Scientific, #37535) for 30 min at room temperature. Primary antibody incubation performed overnight at 4 °C. Primary antibody was removed by washing 3x with PBS (1X). Secondary antibody incubation performed for 1 h at room temperature. TUBB3 was probed with beta-3 tubulin polyclonal antibody (1:50; Invitrogen, #PA5-25655) and goat anti-rabbit IgG (H + L) AF488 (1:250; Invitrogen, #A32731). Ki-67 was probed with Ki-67 (SP6) recombinant monoclonal antibody (1:200; Invitrogen, #MA5-14520) and goat anti-rabbit IgG (H + L) AF594 (1:250; Invitrogen, #A11012). Expression analysis of TUBB3 and Ki-67 was achieved by QIBC using the Celigo Image Cytometer (Nexcelcom Bioscience, USA). Representative images were taken using the EVOS™ FL Imaging System (Invitrogen, #AMF4300, LED, Ex: 357/44 Em: 447/60, Ex: 482/25 Em: 524/24, 4/10 PH, Sony ICX285AQ color CCD, 2/3 in 1360 × 1024, 1.4 Megapixels). Image processing conducted with Fiji (ImageJ)^140^.

### Transfection and assessment of cellular toxicity in post-mitotic neuronal cells

SK-N-BE(2)C cells expressing either *LINE-1* or *AAVS1* targeting sgRNA were cultured in DMEM/F12, GlutaMAX™ supplement (Gibco, #10565018) supplemented with ATRA (10 µM; MedChemExpress, # HY-14649) in 6-well plates (Celltreat, #229106). ATRA-containing growth media was exchanged every 3 days with gradual serum deprivation (10–2%). Cells were maintained in differentiation media until the halting of proliferation and demonstration of distinct morphological changes consistent with neuronal differentiation. Neuronal SK-N-BE(2)C cells expressing either *LINE-1* or *AAVS1* targeting sgRNA were subsequently transfected with Cas9^WT^ or Cas9^D10A^-mRNA (5 µg) using Lipofectamine MessengerMAX Reagent (Invitrogen, #LMRNA008) and assessed for cytotoxic effects at 3 days post-treatment. Cellular toxicity was determined by assessing changes in cell confluency using crystal violet staining (Millipore Sigma, #V5265). Representative images were taken using the EVOS™ FL Imaging System #AMF4300, LED, Sony ICX285AQ color CCD, 2/3 in 1360 × 1024, 1.4 Megapixels) at 4X magnification. Differences in cell confluency determined with ImageJ^140^.

### Micronuclei detection assay

Surviving SK-N-BE(2)C cells expressing the designed sgRNAs were harvested 3 days following electroporation with Cas9^D10A^ mRNA (30 nM) and washed twice in PBS (1X) to remove dead cells and debris before being stained with Hoecsht 33342 (1 µg/mL; Invitrogen, #H1399) for 30 min at room temperature. Cells were pelleted at 1,000 x g and resuspended in Nuclei Prep Buffer (New England Biolabs, #T3052) containing RNase A (Invitrogen, #AM2271) and incubated at room temperature for 5 min. Nuclei Prep Buffer is an incomplete lysis buffer disrupting the cell membrane while leaving nuclei intact. SK-N-BE(2)C nuclei from each condition were analyzed using the MACSQuant® Analyzer 10 Flow Cytometer (Miltenyi Biotec), and further characterized using FlowJo analysis software. Cells were visualized and imaged using the EVOS™ FL Imaging System (Invitrogen, #AMF4300, LED, Ex: 357/44 Em: 447/60, 4/10 PH, Sony ICX285AQ color CCD, 2/3 in 1360 ×1024, 1.4 Megapixels) at 20X magnification.

### Genome copy number variance assay

Genomic DNA was extracted from each of the cell lines using the DNeasy Blood & Tissue Kit according to the manufacturer’s instructions (Qiagen, #69504). DNA concentration was obtained using the Qubit™ dsDNA quantitation high-sensitivity kit (Invitrogen, #Q32851). Genome copy number variance was determined by performing a TaqMan copy number variance real-time qPCR assay using the QuantStudio3 (Applied Biosystems, USA) according to the manufacturer’s instructions using *RPPH1* (RNase P) as an internal control. *MYCN* was amplified using forward (5’ – ACTCTGTCCCTTAAGGAGCAACC – 3’) and reverse (5’ – CCAACCAGGATTGTACAGGTGC – 3’) primers. *MYC* was amplified using forward (5’ – CACAAAGACTCATCCACATGC – 3’) and reverse (5’ – CTCCTGTACTCACAAGCAGC – 3’) primers. *ERBB2* (HER2) was amplified using forward (5‘ – CCACATTCCCAGACTGGACG – 3’) and reverse (5’ – GGTAATACCTGTCTGATGCCAC – 3’) primers. *ALK* was amplified using forward (5’ – CGTTTCAGTCATGGAATGAACTACC – 3’) and reverse (5’ – CATGAGGAAGGGAGAGAAAACGC – 3’) primers. Average gene copy number for each cell line was determined by comparative C_t_. Genome copy numbers were normalized to normal human foreskin fibroblast cells (HFF) as a control.

### MYCN and MCM7 expression analysis by qRT-PCR

Total RNA was extracted from SK-N-BE(2)C or NGP cells using the Monarch® Total RNA Miniprep Kit according to the manufacturer’s instructions (New England Biolabs, #T2010S). Total RNA concentration was obtained using a NanoDrop UV/Vis spectrophotometer (Thermo Scientific, #ND-ONE-W). Reverse transcription was performed using the SuperScript™ III First-strand Synthesis kit according to the manufacturer’s instructions (Invitrogen™, #11752050). Quantitative PCR was performed using PowerTrack™ SYBR Green Master Mix (Applied Biosystems™, #A46012) and the QuantStudio3 (Applied Biosystems, USA) according to the manufacturer’s instructions.

Site-specific cDNA was amplified using the following forward (*MYCN*: 5’ – CACTGAGTATGTCCACTCCCTCC – 3’; *MCM7*: 5’ – CCAAGTCTCAGCTCCTGTCATAC – 3’) and reverse (*MYCN*: 5’ – GTGGCAGTGACTGTCCAGTTTTG – 3’; *MCM7*: 5’ – CCTCTAAGGTCAGTTCTCCACTC – 3’) primers. Expression values were normalized to GAPDH which was amplified using the following forward (5’ – GTCTCCTCTGACTTCAACAGCG – 3’) and reverse (5’ – ACCACCCTGTTGCTGTAGCCAA – 3’) primers. Changes in *MYCN* or *MCM7* expression were determined by comparative C_t_ relative to a mock/untreated control.

### EXO1/DNA2 knockdown

Pre-validated Silencer® Select siRNAs targeting EXO1 or DNA2 (Invitrogen, #AM16708), or Silencer® Select Negative Control siRNA (Invitrogen, #4390843) were delivered to SK-N-BE(2)C cells expressing *LINE-1*, *MYCN*, or *AAVS1* targeting sgRNA using the Lipofectamine RNAiMAX Transfection Reagent (Invitrogen, #13778075) in accordance with the manufacturer’s instructions. Transfected cells were recovered for 24 h prior to downstream application.

### Indel analysis

Genomic DNA was extracted from each of the cell lines using the DNeasy Blood & Tissue Kit (Qiagen, #69504) 3 days post-treatment with Cas9^D10A^ mRNA. DNA concentration was obtained using the Qubit™ dsDNA quantitation high-sensitivity kit (Invitrogen, #Q32851). The sgMYCN-1 target site was amplified using forward (5’ – GTAGAGCATAGTTTGTCTCC – 3’) and reverse (5’ – CTATCTCCTTAACACACAAG – 3’) primers. Amplicon-Seq was performed GENEWIZ (Azenta Life Science, Inc., USA). Raw data was analyzed using the CRISPResso2 deep sequencing analysis webtool^141^.

### Statistics and Reproducibility

No statistical method was used to predetermine sample size. The experiments were not randomized. The Investigators were not blinded to allocation during experiments and outcome assessment. No data were excluded from the analyses. All statistical analyses were carried out using GraphPad Prism (10.2.3) software. Quantitative data, apart from where individual values are demonstrated, represent the mean ± standard deviation. Comparative statistical analyses were conducted using multiple unpaired *t*-tests or two-way ANOVA with a Tukey’s multiple comparison test; ns, *P* > 0.05; *, *P *≤ 0.05; ** *P *≤ 0.01; ***, *P *≤ 0.001; ****, *P *≤ 0.0001.

### Reporting summary

Further information on research design is available in the Nature Portfolio Reporting Summary linked to this article.

## Supplementary information

Supplementary Information

Reporting Summary

Transparent Peer Review file

## Source data

Source Data

## Data Availability

A Reporting Summary for this article is available as a supplementary information file. Illumina sequencing data has been submitted to the Sequence Read Archive accession code PRJNA1251614. Source data are provided as a Source Data file. Source data are provided with this paper.
