# Supplementary material for: Selective targeting of genome amplifications and repeat elements by CRISPR-Cas9 nickases to promote cancer cell death
Source: Nat Commun. 2025 Jun 2;16:5126. doi: 10.1038/s41467-025-60160-2 (PMC12130199; doi:10.1038/s41467-025-60160-2)
Supplement: Supplementary file 1 — Supplementary Information [file 41467_2025_60160_MOESM1_ESM.pdf]

Supplementary Information

Selective targeting of genome amplifications and repeat elements by CRISPR-Cas9 nickases to promote cancer cell death

Matthew B. Hanlon[1], Jason M. Shohet[2], Scot A. Wolfe[1,3]

1 - Department of Molecular, Cell and Cancer Biology, University of Massachusetts Chan Medical School, Worcester, MA 01605, USA
2- Department of Pediatrics, University of Massachusetts Chan Medical School, Worcester, MA 01566, USA
3- Li Weibo Institute for Rare Diseases Research, University of Massachusetts Chan Medical School, Worcester, MA, USA

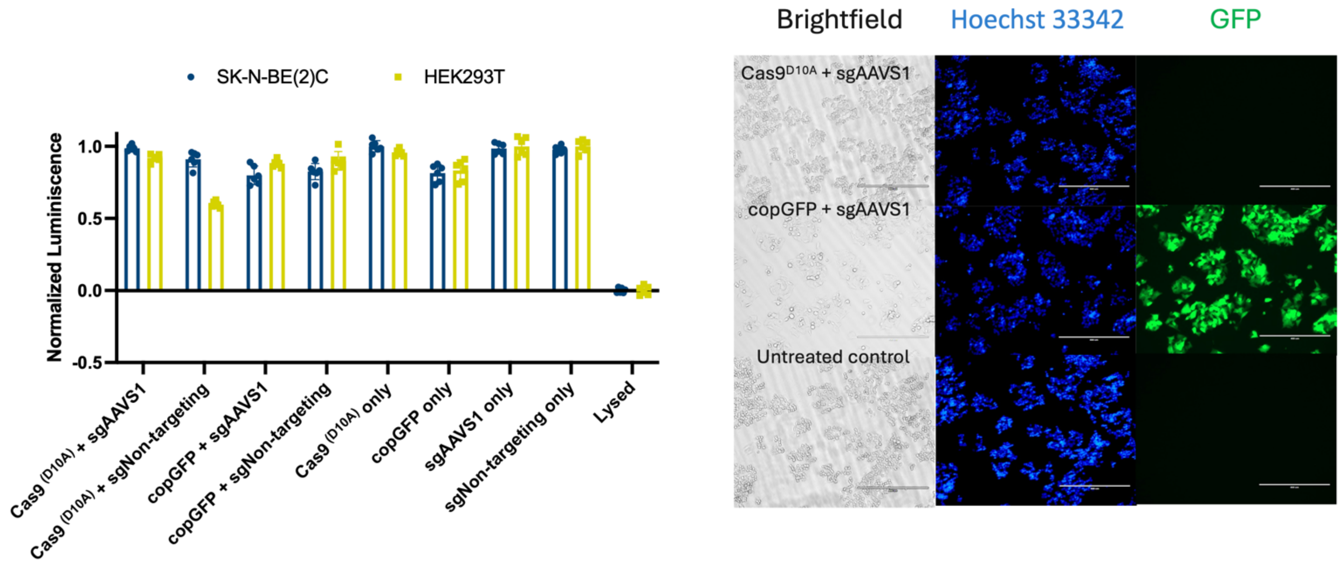

Supplementary Figure 1. Evaluating a set of baseline controls for Cas9^{D10A}-mediated cell-killing. Impacts of electroporation of various reagents on the cell viability of *MYCN*-amplified SK-N-BE(2)C cells and *MYCN* non-amplified, non-neuroblastoma HEK293T cells. (Left) Changes in cell viability were assessed at 3-days post-electroporation for an array of negative controls: Cas9^{D10A}-mRNA (30 nM) + sgAAVS1 (30 μM); Cas9^{D10A}-mRNA (30 nM) + sgNon-targeting (30 μM); copGFP-mRNA (30 nM) + sgAAVS1 (30 μM); copGFP-mRNA (30 nM) + sgNon-targeting (30 μM); Cas9^{D10A}-mRNA only (30 nM); copGFP-mRNA only (30 nM); sgAAVS1 only (30 μM); and sgNon-targeting only (30 μM; n = 6). Negligible differences were observed between control candidates. Cas9^{D10A} targeting *AAVS1*, a well characterized safe-harbor locus, offers a representative, negative control for the direct comparison of Cas9^{D10A}-mediated cell-killing efficacy. (Right) Fluorescent microscopy confirmation of the successful delivery of copGFP-mRNA to SK-N-BE(2)C cells at 3-days post-electroporation.

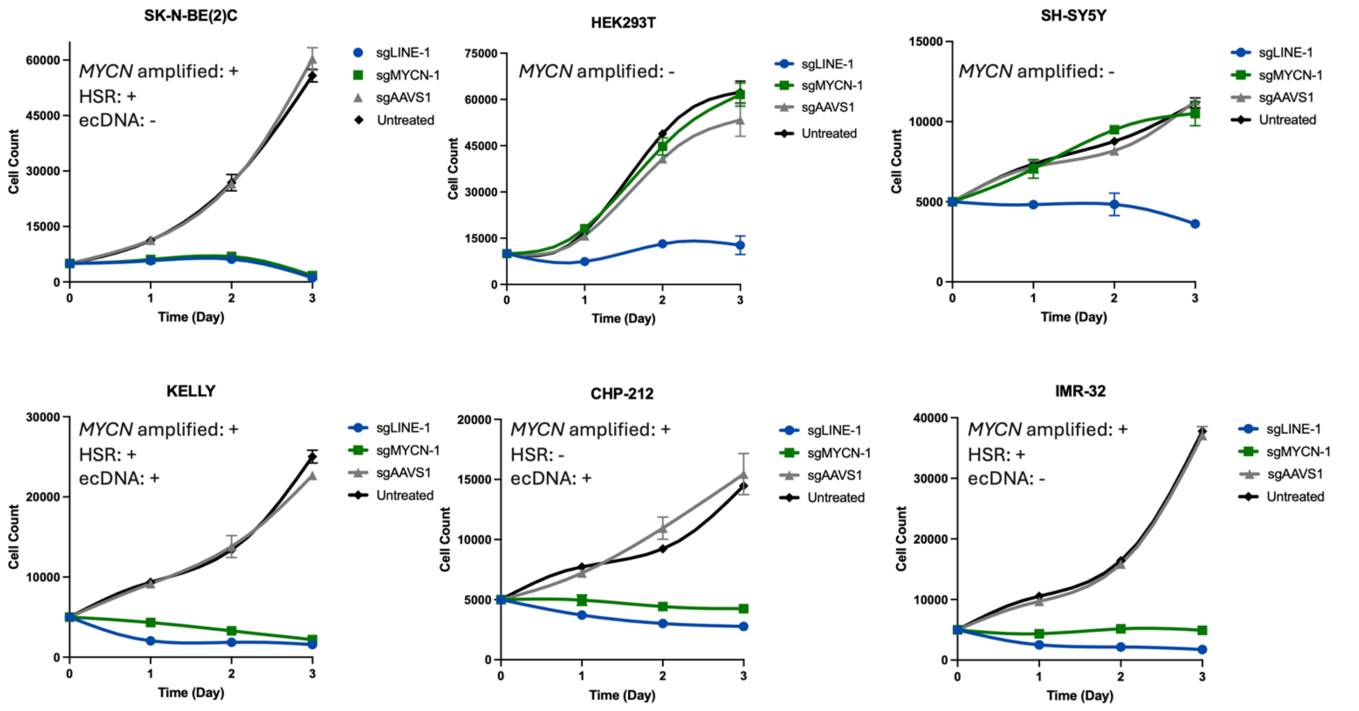

Supplementary Figure 2. Cas9^{D10A}-mediated DNA damage at the *MYCN* locus promotes population collapse in *MYCN*-amplified neuroblastoma cells. Proliferation of *MYCN*-amplified SK-N-BE(2)C, KELLY, NGP, CHP-212; *MYCN* non-amplified SH-SY5Y; *MYCN* non-amplified, non-neuroblastoma HEK293T cells assessed by quantitative image based cytometry (QIBC) assisted cell counting at 1-, 2-, and 3-days post-treatment with Cas9^{D10A}-mRNA (30 nM) targeting *LINE-1*, *MYCN*, or *AAVS1* (n = 3). All cell lines targeted at *LINE-1* display substantial growth inhibition and population collapse, whereas all cell lines targeted at *AAVS1* display no appreciable alterations in proliferation rate relative to an untreated control. Targeting *MYCN* was shown to inhibit growth and promote population collapse in *MYCN*-amplified neuroblastoma cells, wheres proliferation rates in *MYCN* non-amplified cells (SH-SY5Y & HEK293T) remained comparable to *AAVS1*-targeted and untreated controls.

A

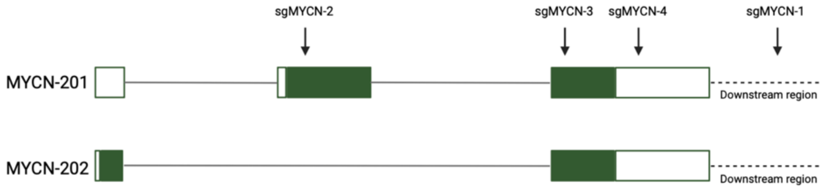

sgRNA	Sequence (5'– 3')	Strand	Feature
sgMYCN-1	CAATGGAGACCCCATATGGG	Forward	Non-coding
sgMYCN-2	CAGCTCCGAGCCCCCGAGCT	Forward	Exon/Intron
sgMYCN-3	GGACCCAGGGCTGCGTTCTT	Reverse	Exon
sgMYCN-4	CACCAGTGTGGGGTTCTGCT	Forward	3'UTR

B

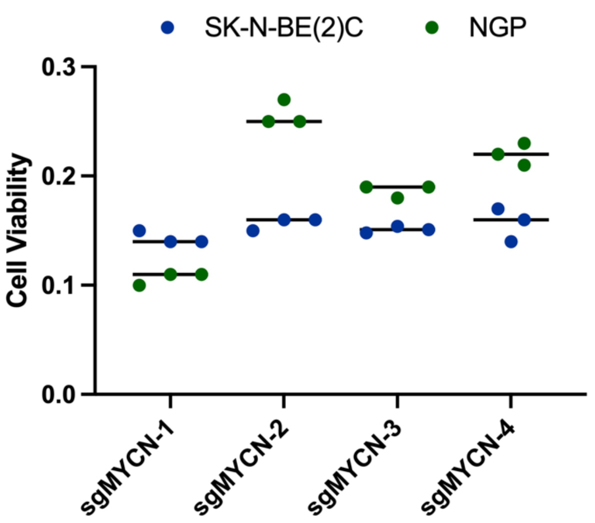

Supplementary Figure 3. Cas9^{D10A}-mediated cell-killing is not target site dependent within the *MYCN* locus. A) A schematic overview of the *MYCN* locus and table corresponding to each of the four tested *MYCN* Cas9^{D10A} target sites. Additional target sites include *MYCN* exon 2/intron depending on the transcript spice variant (sgMYCN-2), exon 2 (sgMYCN-3), and 3'UTR (sgMYCN-4). **B)** SK-N-BE(2)C or NGP *MYCN*-amplified neuroblastoma cells expressing an sgRNA corresponding to one of four *MYCN* target sites were treated with Cas9^{D10A} – mRNA (30 nM) and assessed for changes in cell viability at 3-days post-treatment relative to an *AAVS1* targeted control (n = 3). Reduction in cell viability at 3-days post-treatment was comparable between all target sites. Data are presented as individual values around the median (black line).

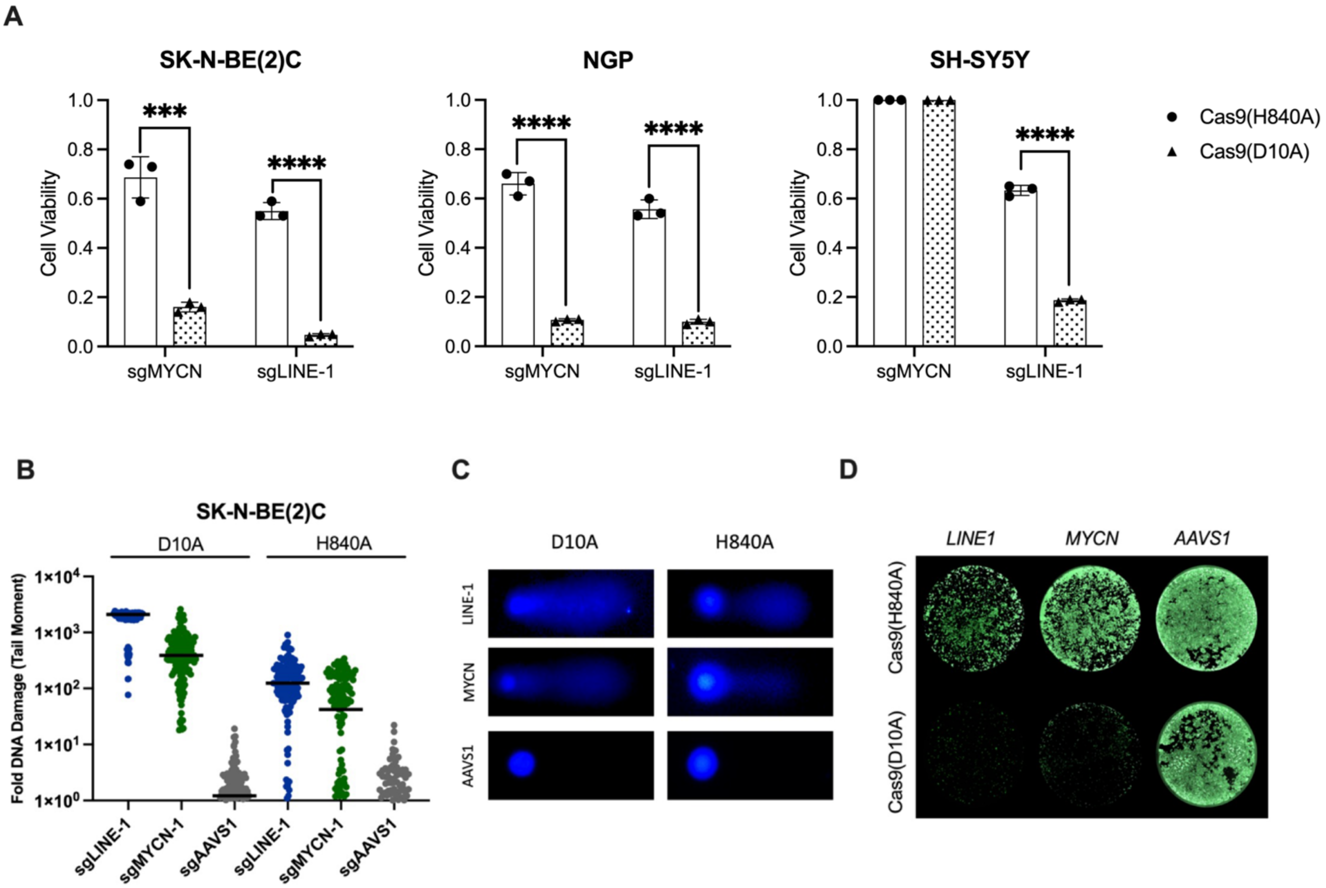

Supplementary Figure 4. Cell-killing potential of Cas9^{D10A} and Cas9^{H840A} nickases are not equivalent in *MYCN*-amplified neuroblastoma cells. A) Comparison of cell viability for *MYCN*-amplified SK-N-BE(2)C and NGP, and *MYCN* non-amplified SH-SY5Y neuroblastoma cell lines expressing *LINE-1* or *MYCN* targeting sgRNA at 3-days post-treatment with Cas9^{D10A} or Cas9^{H840A} mRNA (30 nM; n = 3). Outcomes demonstrate a disproportionate response to Cas9^{D10A} nickase-induced DNA damage. Data are presented as mean ± s.d. normalized relative to viability of cells expressing *AAVS1* targeting sgRNA treated with Cas9^{D10A}. Data were analyzed multiple unpaired t-tests; ns, P > 0.05; *, P ≤ 0.05; ** P ≤ 0.01; ***, P ≤ 0.001; ****, P ≤ 0.0001. **B)** Cumulative Cas9 nickase-mediated DNA damage in SK-N-BE(2)C cells expressing *LINE*-1, *MYCN*, or *AAVS1* targeting sgRNA was assessed by alkaline comet assay at 3-days post-treatment with either Cas9^{D10A} or Cas9^{H840A}-mRNA (30 nM; n = 150). DNA damaging activity of Cas9^{D10A} is superior to Cas9^{H840A} when targeting *LINE-1* or *MYCN*, with no appreciable difference when targeting *AAVS1*. Data are presented as individual values around the median (black line). **C)** Representative comets of SK-N-BE(2)C cells from panel B that were treated with Cas9^{D10A} or Cas9^{H840A} targeting *LINE-1*, *MYCN*, or *AAVS1*. **D)** Representative image comparing the cell-killing potential of Cas9^{D10A} or Cas9^{H840A} in SK-N-BE(2)C cells expressing either *LINE-1*, *MYCN*, or *AAVS1* targeting sgRNA and stained with calcein AM viability stain 3 days following Cas9 nickase mRNA delivery.

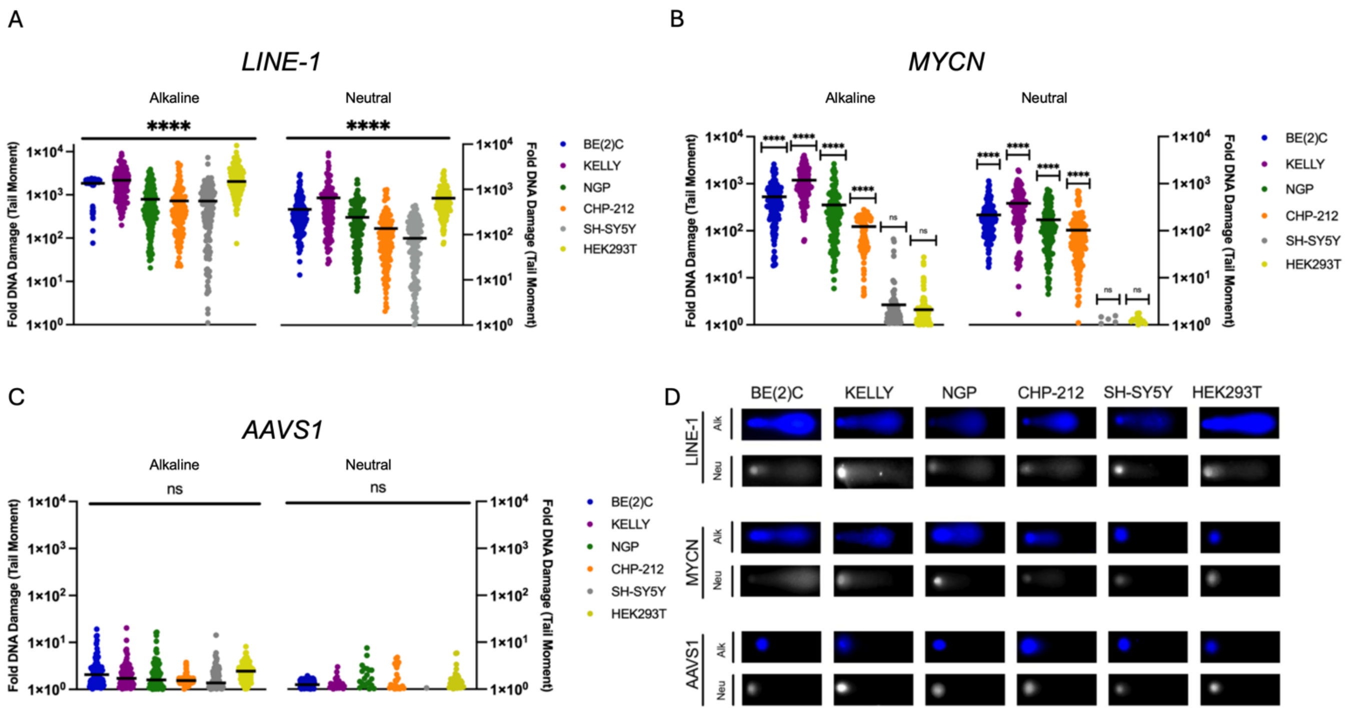

Supplementary Figure 5. Cas9^{D10A} induces substantial DNA damage across a variety of *MYCN*-amplified neuroblastoma cell lines. A – C) Cumulative (SSBs and DSBs; alkaline) and DSB-specific (neutral) DNA damage was assessed in individual *MYCN*-amplified SK-N-BE(2)C, KELLY, NGP, CHP-212 neuroblastoma cells; *MYCN* non-amplified SH-SY5Y neuroblastoma cells; and HEK293T non-neuroblastoma cells by comet assay at 3-days post-treatment with Cas9^{D10A}-mRNA (30 nM). Each cell line was modified to express *LINE-1*, *MYCN*, or *AAVS1* targeting sgRNA. **A)** All cells targeted at *LINE-1* with Cas9^{D10A} displayed a significant enrichment in DNA damage (n = 150). **B)** Only *MYCN*-amplified neuroblastoma cells targeted at the *MYCN* locus displayed a significant enrichment in DNA damage (n = 150). **C)** All cells targeted at *AAVS1* displayed no appreciable enrichment in DNA damage (n = 150). Quantification of DNA damage is relative to a cell line-specific mock/untreated control. Data are presented as individual values around the median (black line) and analyzed using multiple unpaired t-tests; ns, $P > 0.05$; *, $P \leq 0.05$; ** $P \leq 0.01$; ***, $P \leq 0.001$; ****, $P \leq 0.0001$ using untreated cells as a baseline control. **D)** Representative images of Cas9^{D10A}-treated cell comets from panels A, B & C.

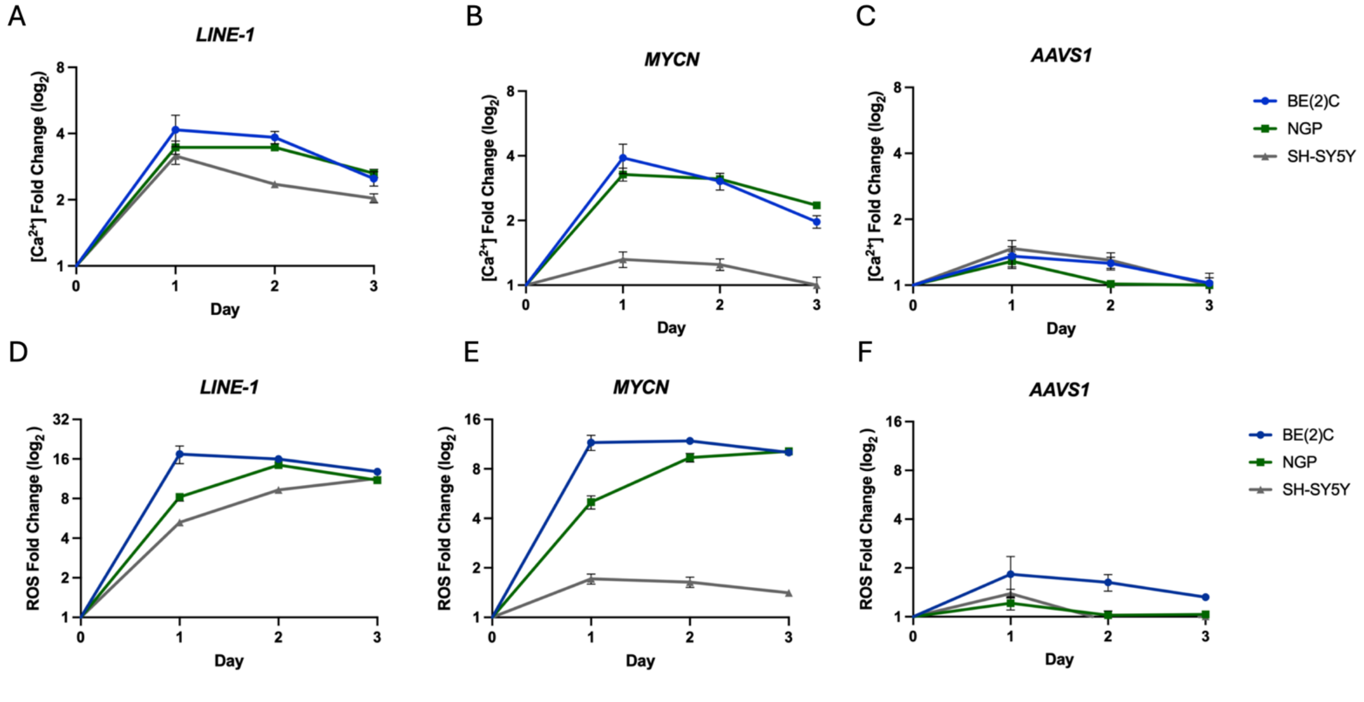

Supplementary Figure 6. Cas9^{D10A}-treated, MYCN-amplified neuroblastoma cells display metabolic markers of replication stress. A – C) QIBC analysis of intracellular Ca^{2+} influx in SK-N-BE(2)C, NGP and SH-SY5Y cells expressing *LINE-1*, *MYCN* or *AAVS1* targeting sgRNA at 1-, 2-, and 3-days post treatment with Cas9^{D10A} mRNA (30 nM). Ca^{2+} flux quantified with Fluo-4 AM indicator relative to a mock/untreated control (n = 3). **D – F)** QIBC analysis of intracellular ROS influx in SK-N-BE(2)C, NGP and SH-SY5Y cells expressing *LINE-1*, *MYCN* or *AAVS1* targeting sgRNA at 1-, 2-, and 3-days post treatment with Cas9^{D10A} mRNA (30 nM). ROS flux quantified with ROS indicator stain relative to a mock/untreated control (n = 3). Data in panels A-F are presented as mean ± s.d. after normalization to the control.

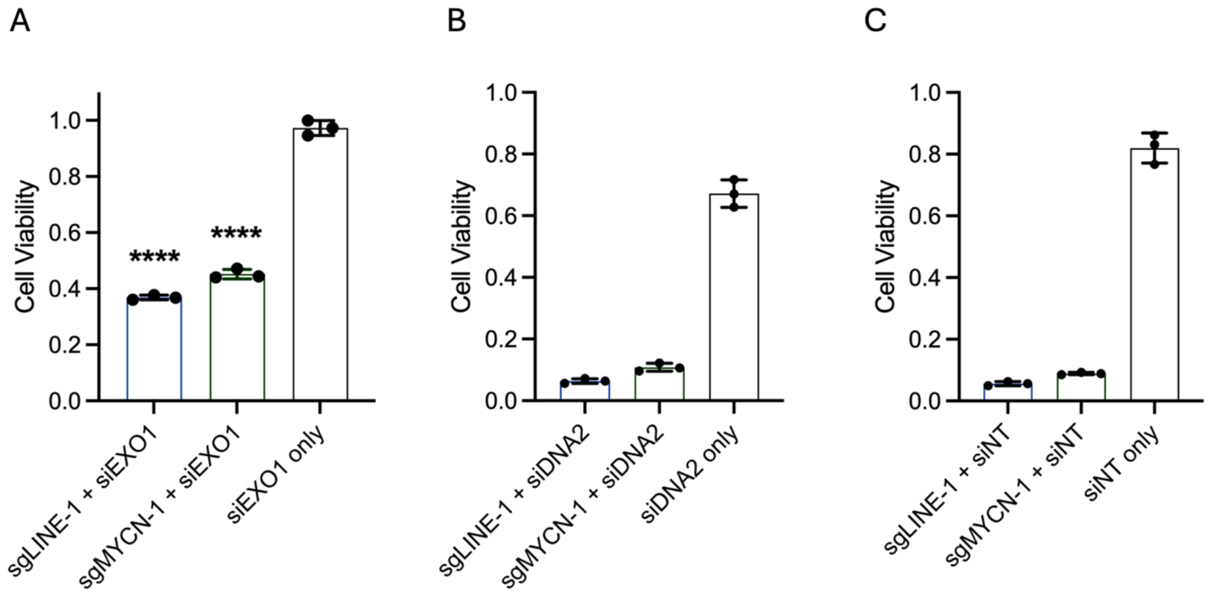

Supplementary Figure 7. Downregulation of EXO1, not DNA2, attenuates Cas9^{D10A} – mediated toxicity. A – C) SK-N-BE(2)C cells expressing *LINE-1* or *MYCN* targeting sgRNA were transfected with an siRNA targeting EXO1, DNA2, or a non-targeting control, recovered for 24 hours, and then treated with Cas9^{D10A}-mRNA (30 nM). Changes in cell viability were assessed at 3-days post-treatment with Cas9^{D10A} (n = 3). Data are presented as mean ± s.d. relative to an *AAVS1* targeted control.

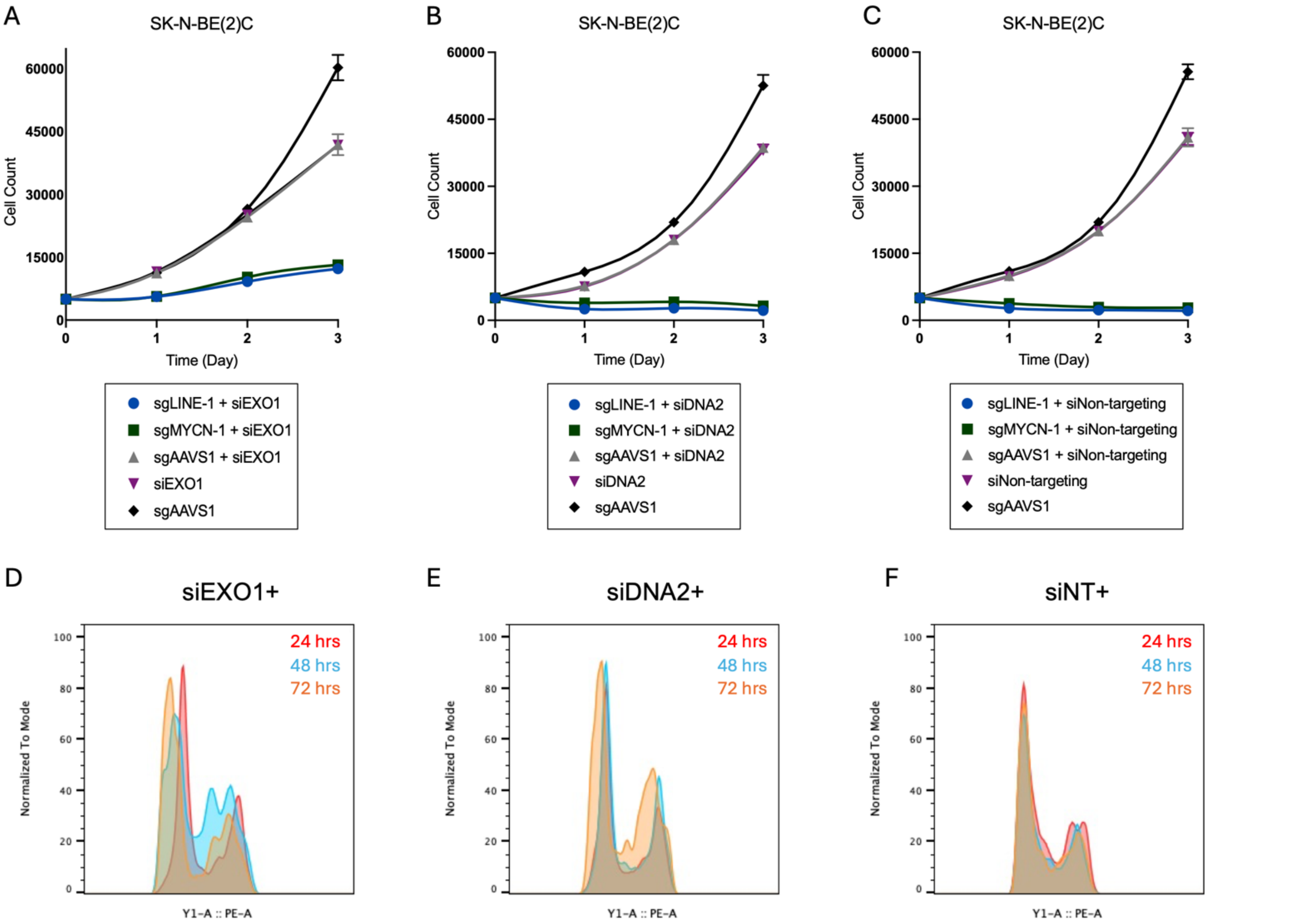

Supplementary Figure 8. Downregulation of EXO1 confers tolerance to Cas9^{D10A}-induced toxicity in *MYCN*-amplified neuroblastoma cells. A – C) Proliferation of *MYCN*-amplified SK-N-BE(2)C cells pre-treated with siRNA targeting EXO1, DNA2, or non-targeting control was assessed by quantitative image based cytometry (QIBC) assisted cell counting at 1-, 2-, and 3-days post-treatment with Cas9^{D10A}-mRNA (30 nM) targeting *LINE-1*, *MYCN*, or *AAVS1* (n = 3). Data are presented as mean ± s.d. **D – F)** Representative histograms of flow cytometric cell cycle analysis of SK-N-BE(2)C cells transfected with siRNA targeting EXO1, DNA2, or a non-targeting control without Cas9^{D10A} at 1-, 2-, and 3-days post-transfection (n = 3).

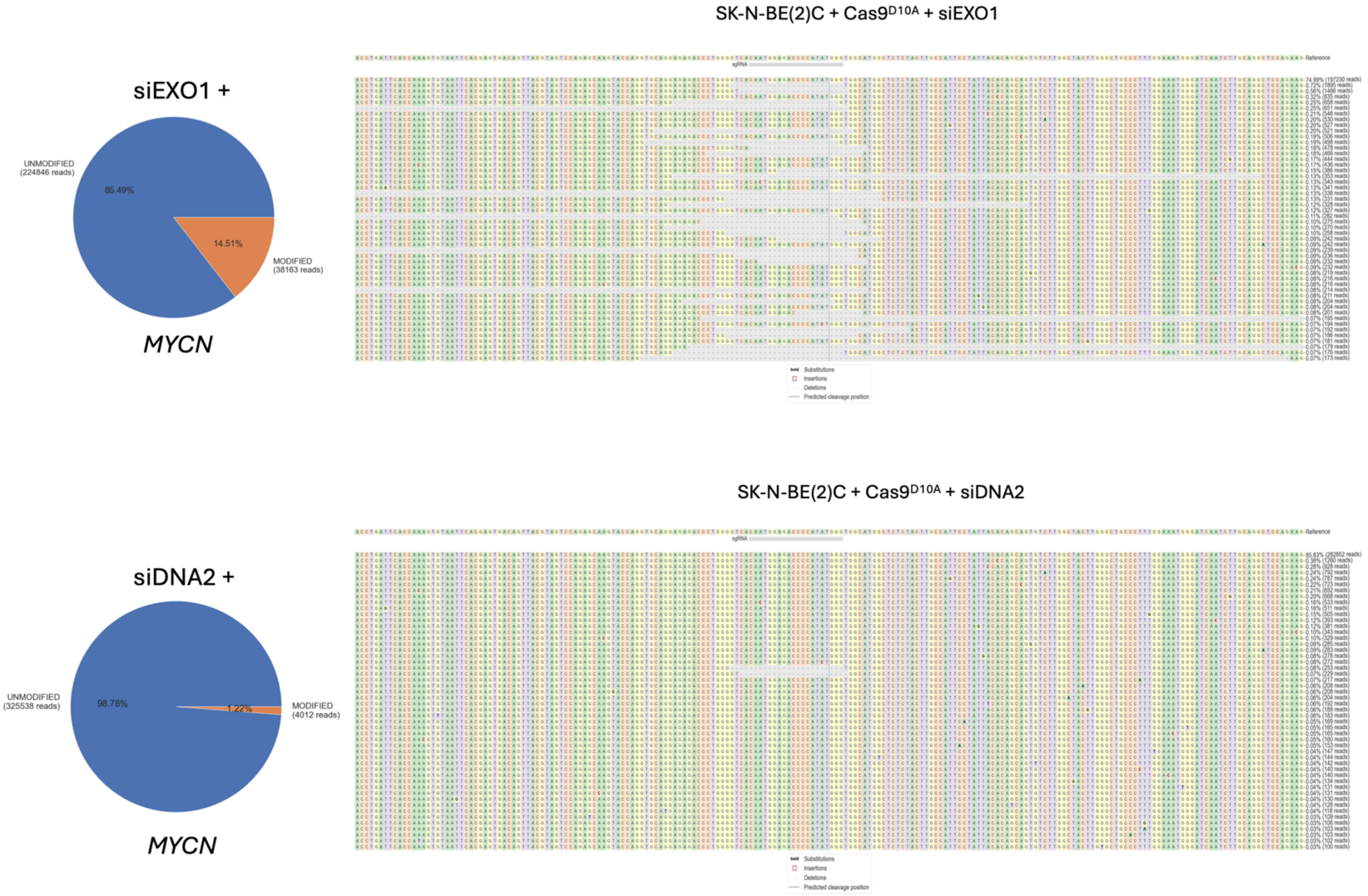

Supplementary Figure 9. Downregulation of EXO1, but not DNA2 alters DNA repair outcomes for Cas9^{D10A}-mediated editing in *MYCN*-amplified neuroblastoma cells. Top: Amplicon-sequencing of the sgMYCN-1 target site from genomic DNA isolated from surviving SK-N-BE(2)C cells pre-treated with a EXO1 siRNA at 3-days post-treatment with Cas9^{D10A}-mRNA (30 nM). **Bottom:** Amplicon-sequencing of the sgMYCN-1 target site from genomic DNA isolated from surviving SK-N-BE(2)C cells pre-treated with a DNA2 siRNA at 3-days post-treatment with Cas9^{D10A}-mRNA (30 nM). Editing outcomes suggest that the downregulation of EXO1 activity alters DNA repair outcomes of Cas9^{D10A}-mediated, replication-dependent DSBs as evident by the increased sequence modifications at the sgMYCN-1 target site.

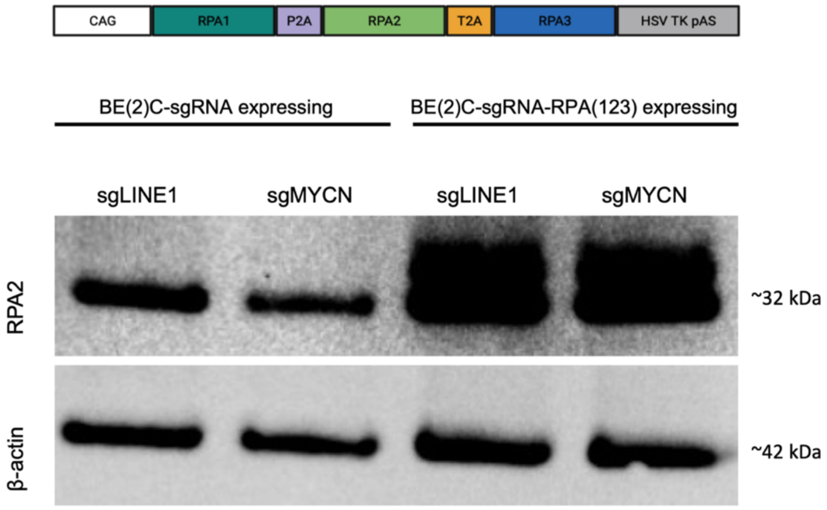

Supplementary Figure 10. Construction and verification of RPA overexpression. Schematic diagram of the RPA(123) expression cassette (top) and Western blot verification of RPA overexpression in SK-N-BE(2)C cells. RPA2 expressed from the transgene is larger due to the P2A-T2A ribosome skipping sequences appended to the termini.

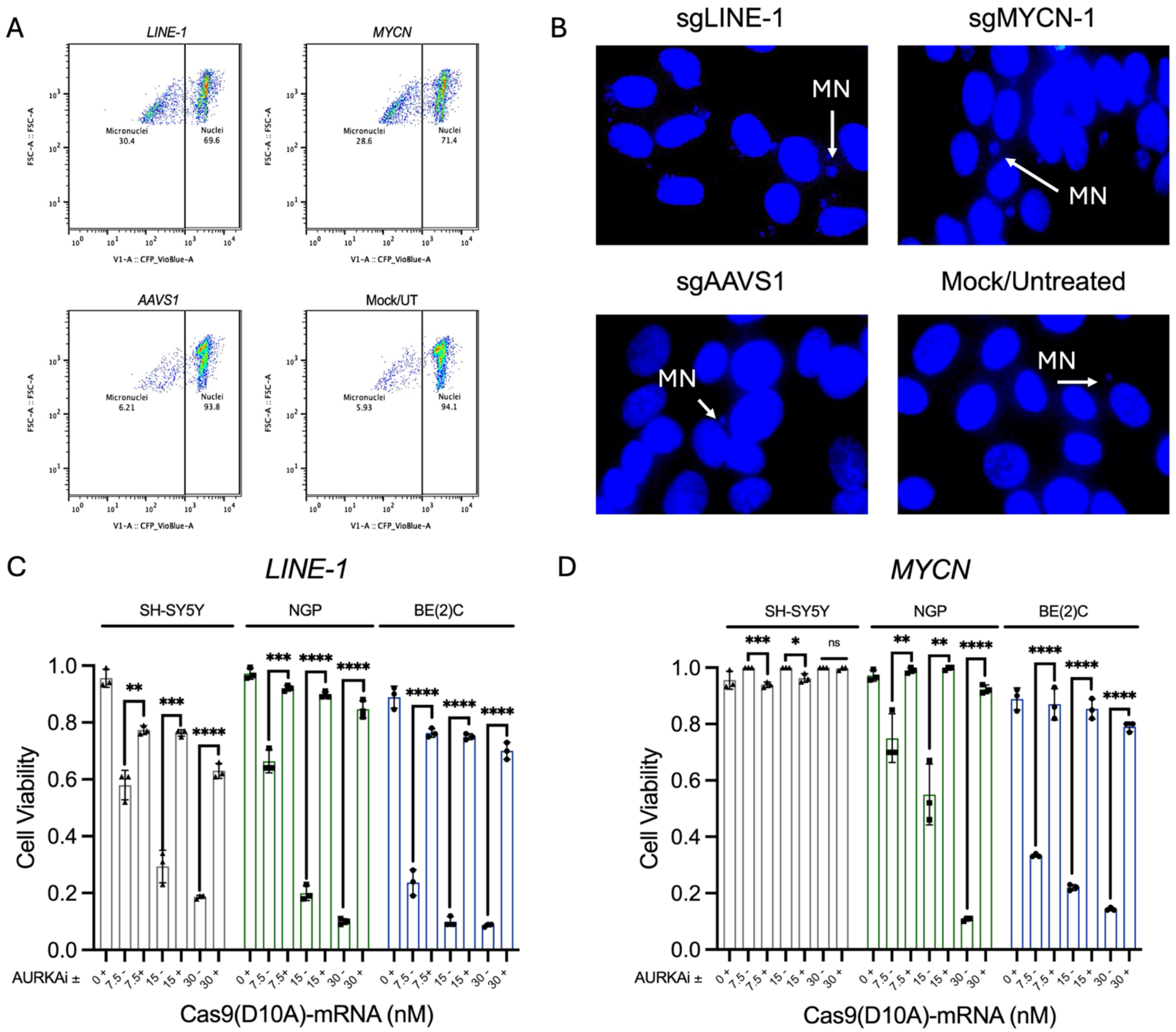

Supplementary Figure 11. Surviving *MYCN*-amplified neuroblastoma cells demonstrate an enrichment in micronuclei. A) Flow cytometric analysis of nuclei and micronuclei for SK-N-BE(2)C cells expressing *LINE-1*, *MYCN* or *AAVS1* targeting sgRNA at 3-days post-treatment with $Cas9^{D10A}$-mRNA (30 nM). Surviving *LINE-1* and *MYCN* targeted cells display a substantial enrichment in micronuclei. **B)** Representative images of micronuclei formation in SK-N-BE(2)C cells expressing *LINE-1*, *MYCN*, or *AAVS1* targeting sgRNA at 3-days post-treatment with $Cas9^{D10A}$-mRNA (30 nM). **C & D)** Treatment of *MYCN*-amplified SK-N-BE(2)C or NGP, or *MYCN* non-amplified SH-SY5Y neuroblastoma cells expressing C) *LINE-1* or D) *MYCN* targeting sgRNA with $Cas9^{D10A}$-mRNA at increasing doses in the absence or presence of the AURKA inhibitor, alisertib (0.5 μM). Inhibition of AURKA in the presence of $Cas9^{D10A}$ is protective, as indicated in the reduced cell-killing efficacy of $Cas9^{D10A}$ at 3 days post-treatment (n = 3). These observations support the involvement of cell division in $Cas9^{D10A}$-induced toxicity. Data are presented as mean ± s.d. normalized relative to viability of cells expressing *AAVS1* targeting sgRNA treated with $Cas9^{D10A}$. Data were analyzed multiple unpaired t-tests; ns, $P > 0.05$; *, $P \leq 0.05$; ** $P \leq 0.01$; ***, $P \leq 0.001$; ****, $P \leq 0.0001$.

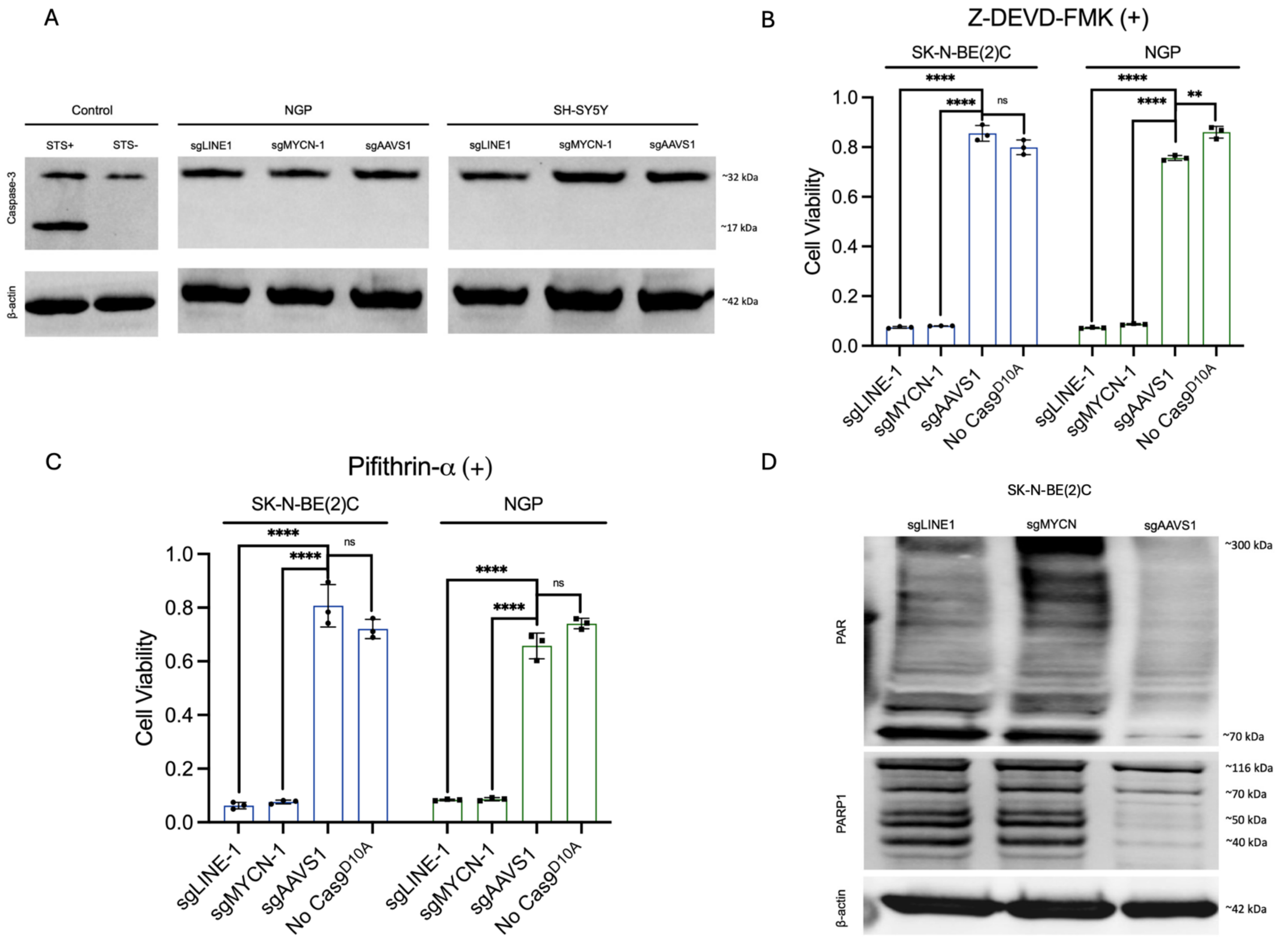

Supplementary Figure 12. Cas9^{D10A}–treated neuroblastoma cells do not display markers of apoptosis. **A)** Western blot of caspase-3 in p53-WT, NGP and SH-SY5Y neuroblastoma cells. Activation of caspase-3 was assessed at 3-days post-treatment with Cas9^{D10A}-mRNA (30 nM). SH-SY5Y cells co-incubated with staurosporine (STS; 1 μM) were included as a positive control for caspase-3 activation. No caspase-3 activation was detected in NGP or SH-SY5Y cells when targeting *LINE-1*, *MYCN*, or *AAVS1* with Cas9^{D10A}. **B & C)** *MYCN*-amplified neuroblastoma cell lines, SK-N-BE(2)C (p53-deficient) and NGP (p53-WT) expressing *LINE-1* or *MYCN* targeting sgRNA exhibit no appreciable change in Cas9^{D10A} – mediated cell-killing efficacy when supplemented with B) Z-DEVD-FMK (18 μM) or C) PFTα (20 μM) at 3-days post-treatment with Cas9^{D10A} – mRNA (30 nM; n = 3). Data are presented as mean ± s.d. normalized relative to viability of cells expressing *AAVS1* targeting sgRNA treated with Cas9^{D10A}. Data were analyzed using multiple unpaired t-tests; ns, $P > 0.05$; *, $P \leq 0.05$; ** $P \leq 0.01$; ***, $P \leq 0.001$; ****, $P \leq 0.0001$ using *AAVS1* sgRNA expressing cells as a baseline control. **D)** Western blot of SK-N-BE(2)C cells expressing *LINE-1*, *MYCN*, or *AAVS1* targeting sgRNA at 3 days post-treatment with Cas9^{D10A}-mRNA (30 nM) demonstrates enrichment of PAR, indicative of PARP1 hyperactivation, when targeting *LINE-1* or *MYCN*. The PARP1 cleavage pattern when targeting *LINE-1* or *MYCN* is consistent with necrotic cell death as opposed to apoptotic cell death.

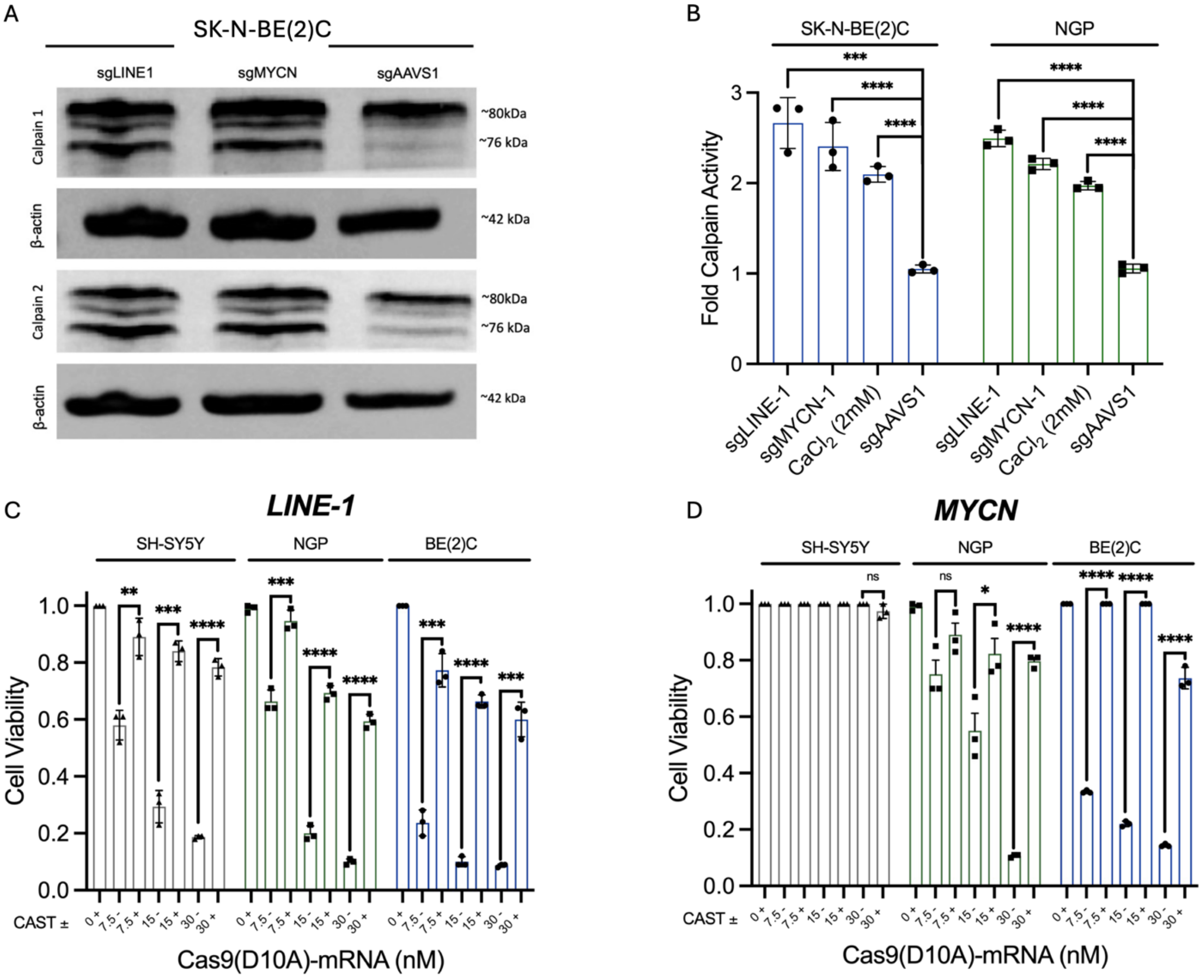

Supplementary Figure 13. *MYCN*-amplified neuroblastoma cells demonstrate calpain activation post-treatment with Cas9^{D10A}. A) Western blot of SK-N-BE(2)C cells expressing *LINE-1*, *MYCN*, or *AAVS1* targeting sgRNA treated with Cas9^{D10A}-mRNA (30 nM) at 3-days post-treatment suggests the activation of calpains as evident by their autoproteolytic cleavage. **B)** Calpain activity assessment in *MYCN*-amplified SK-N-BE(2)C and NGP cells expressing *LINE-1*, *MYCN*, or *AAVS1* sgRNA at 3 days post-treatment with Cas9^{D10A}-mRNA (30 nM; n = 3). Calpain activity was induced in both cell lines through the addition of $CaCl_2$ in the growth media. Data are presented as mean ± s.d. normalized relative to calpain activity of cells expressing *AAVS1* targeting sgRNA treated with Cas9^{D10A}. Data were analyzed multiple unpaired t-tests; ***, $P \leq 0.001$; ****, $P \leq 0.0001$. **C & D)** Impact on cell viability of treatment of SK-N-BE(2)C, NGP, and SH-SY5Y cells expressing *LINE-1* or *MYCN* targeting sgRNA with Cas9^{D10A}-mRNA at increasing doses in the absence or presence of a calpain inhibitor, calpastatin (CAST; 20 nM). Inhibition of calpains in the presence of Cas9^{D10A} is protective, as evident by the reduced cell-killing efficacy of Cas9^{D10A} at 3 days post-treatment (n = 3). Data are presented as mean ± s.d. normalized relative to viability of cells expressing *AAVS1* targeting sgRNA treated with Cas9^{D10A}. Data were analyzed multiple unpaired t-tests; ns, $P > 0.05$; *, $P \leq 0.05$; ** $P \leq 0.01$; ***, $P \leq 0.001$; ****, $P \leq 0.0001$.

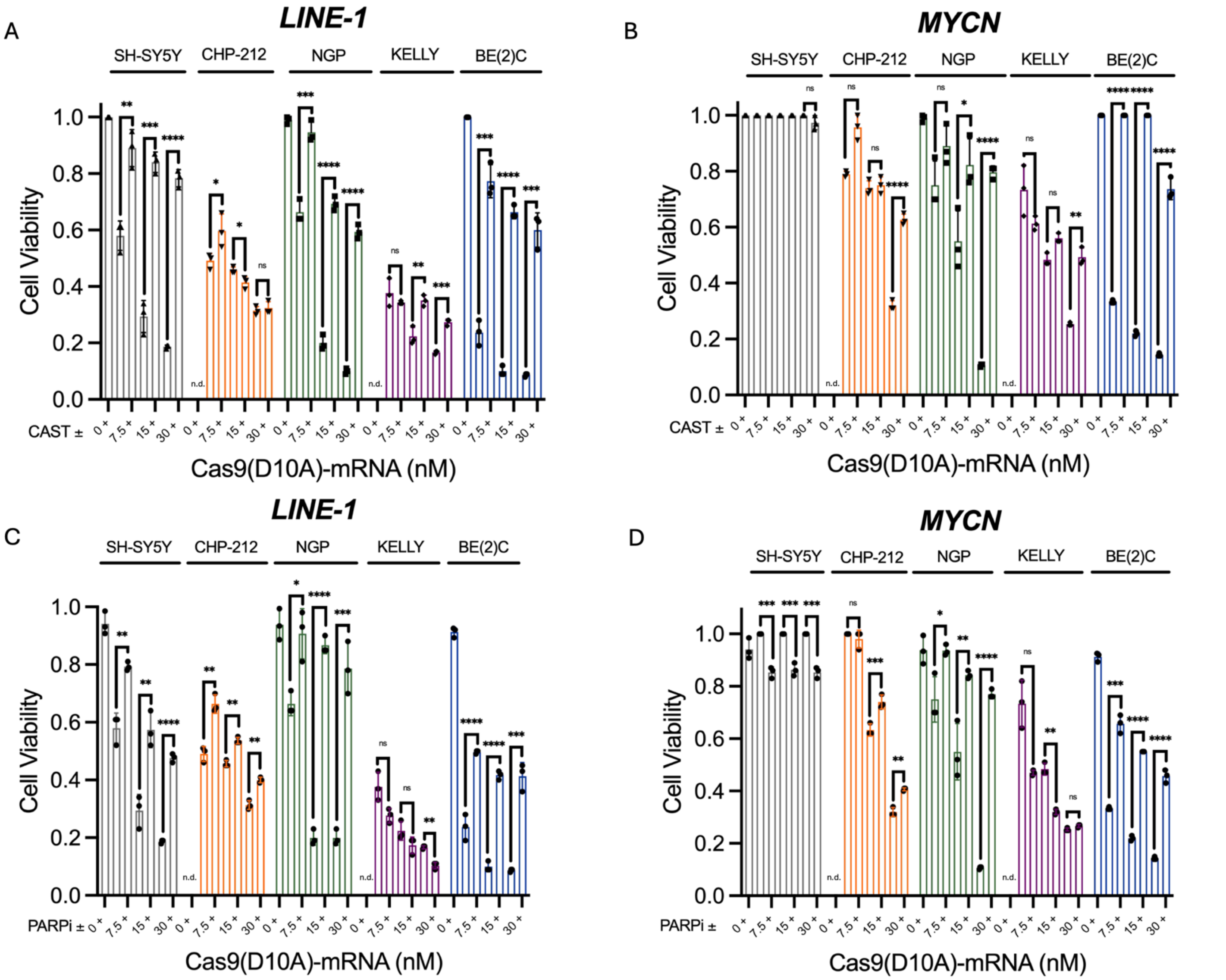

Supplementary Figure 14. Cas9^{D10A}-mediated toxicity attenuated by PARP or calpain inhibitors. A & B) Treatment of *MYCN*-amplified SK-N-BE(2)C, KELLY, NGP, and CHP-212 or *MYCN* non-amplified SH-SY5Y neuroblastoma cells expressing A) *LINE-1* or B) *MYCN* targeting sgRNA with Cas9^{D10A}-mRNA (7.5, 15, or 30 nM; increase in concentration marked by black triangle) without or with the calpain inhibitor, calpastatin (CAST; 20 nM). Inhibition of calpains in the presence of Cas9^{D10A} is protective, as indicated in the reduced cell-killing efficacy of Cas9^{D10A} at 3-days post-treatment (n = 3). **C & D)** Treatment of *MYCN*-amplified SK-N-BE(2)C, KELLY, NGP, and CHP-212 or *MYCN* non-amplified SH-SY5Y neuroblastoma cells expressing C) *LINE-1* or D) *MYCN* targeting sgRNA with Cas9^{D10A}-mRNA (7.5, 15, or 30 nM; increase in concentration marked by black triangle) without or with the PARP inhibitor, rucaparib (10 μM). Inhibition of PARP1 in the presence of Cas9^{D10A} is protective, as indicated in the reduced cell-killing efficacy of Cas9^{D10A} at 3-days post-treatment (n = 3). PARP1 inhibition only had a modest effect on cell killing in KELLY and CHP-212 cells. A modest reduction in cell viability in SH-SY5Y cells in the presence of the PARP inhibitor is likely due to non-specific toxicity. Data are presented as mean ± s.d. normalized relative to viability of cells expressing *AAVS1* targeting sgRNA treated with Cas9^{D10A}. Data were analyzed multiple unpaired t-tests; ns, $P > 0.05$; *, $P \leq 0.05$; ** $P \leq 0.01$; ***, $P \leq 0.001$; ****, $P \leq 0.0001$.

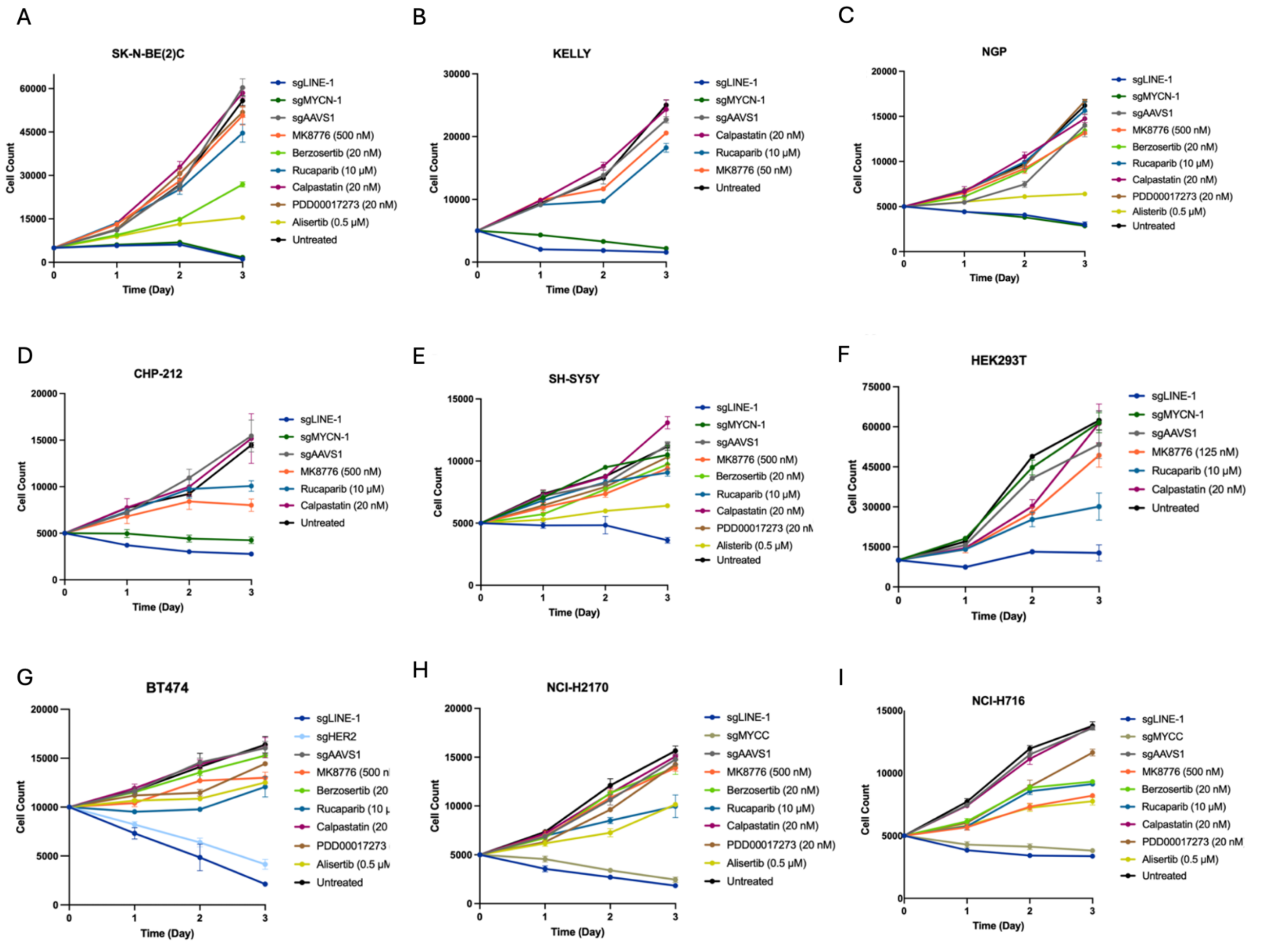

Supplementary Figure 15. Evaluation of cell proliferation in the presence Cas9^{D10A} targeting different loci or small molecule inhibitors (SMIs) to be used in combination with Cas9^{D10A}. A – I) Population dynamics of *MYCN*-amplified; A) SK-N-BE(2)C, B) KELLY, C) NGP, D) CHP-212; E) *MYCN* non-amplified SH-SY5Y; F) non-neuroblastoma HEK293T; G) *ERBB2* (HER2)-amplified BT-474; and *MYC*-amplified H) NCI-H2170 and I) NCI-H716 sgRNA expressing cells monitored at 1-, 2-, and 3-days post-treatment with Cas9^{D10A}-mRNA (30 nM; n = 3) or in the presence of each SMIs at concentrations ≤IC_{50} in the absence of Cas9^{D10A} co-delivery. Experiments designated by an sgRNA (e.g. sgLINE-1, sgAAVS1, sgMYCN-1, etc.) indicate cells that express that particular sgRNA and were treated with Cas9^{D10A}. Targeting of *AAVS1* with Cas9^{D10A} did not appreciably impact in the proliferation rate of any of the tested cell lines relative to a mock/untreated control. These observations are consistent with Cas9^{D10A}-mediated cell-killing when targeting gene amplifications as opposed to simply a substantial reduction in the rate of cell proliferation.

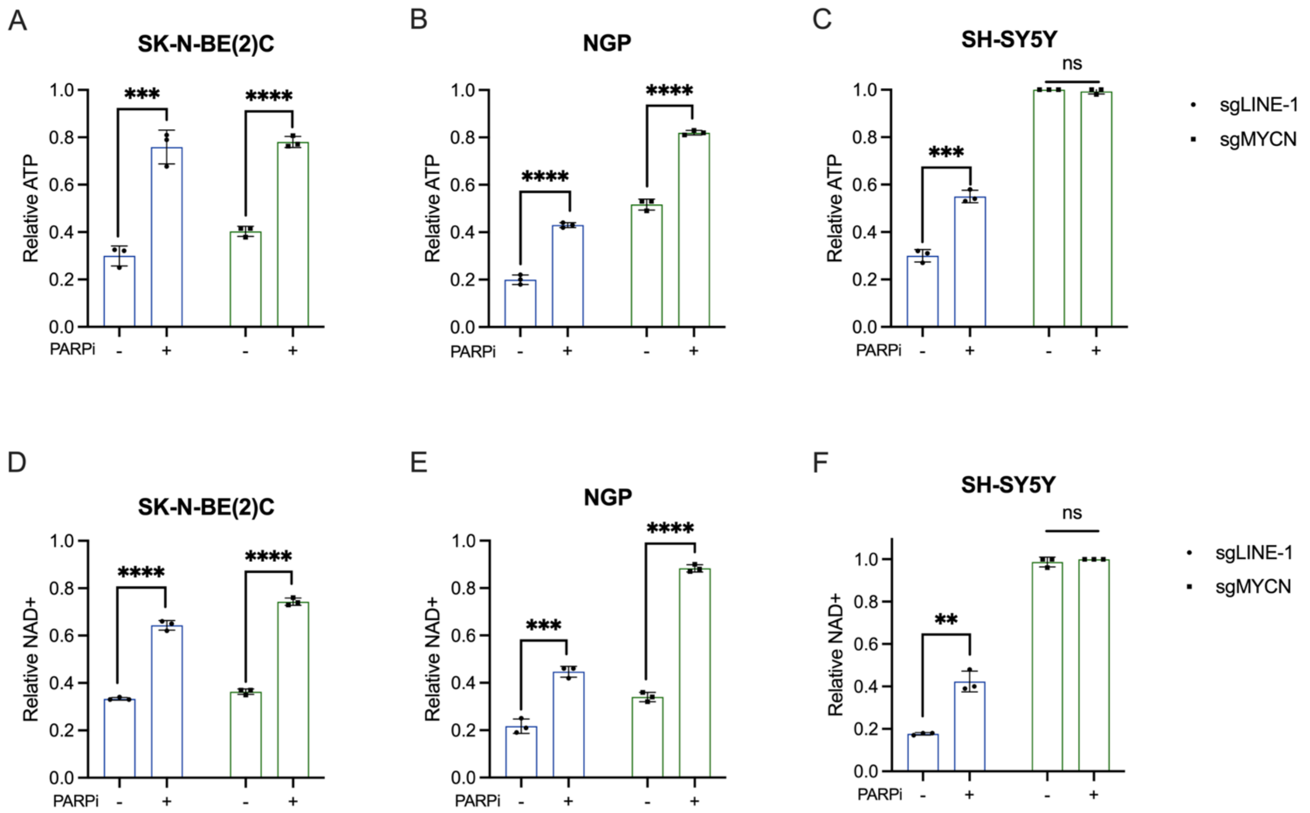

Supplementary Figure 16. Cas9^{D10A}-treated, neuroblastoma cells display markers of PARP1 hyperactivation and necrotic cell death when targeting amplified loci. A – F) Intracellular ATP and NAD+ levels in A & D) SK-N-BE(2)C, B & E) NGP, or C & F) SH-SY5Y cell lines expressing *LINE-1*, *MYCN*, or *AAVS1* targeting sgRNA at 3 days post-treatment with Cas9^{D10A}-mRNA (30 nM; n = 3). Depletion of ATP and NAD+ is consistent with PARP1 hyperactivation subsequent to DNA damage and is observed post-treatment when targeting *LINE-1* in all cell lines, and when targeting *MYCN* in *MYCN*-amplified neuroblastoma cell lines. Supplementation of Cas9^{D10A} treatment with the PARP inhibitor (PARPi), rucaparib (10 µM) significantly attenuates the depletion of ATP and NAD+ post-treatment with Cas9^{D10A}. Data are presented as mean ± s.d. normalized relative to ATP and NAD+ levels in cells expressing *AAVS1* targeting sgRNA treated with Cas9^{D10A}. Data were analyzed multiple unpaired t-tests; ns, $P > 0.05$; *, $P \leq 0.05$; ** $P \leq 0.01$; ***, $P \leq 0.001$; ****, $P \leq 0.0001$.

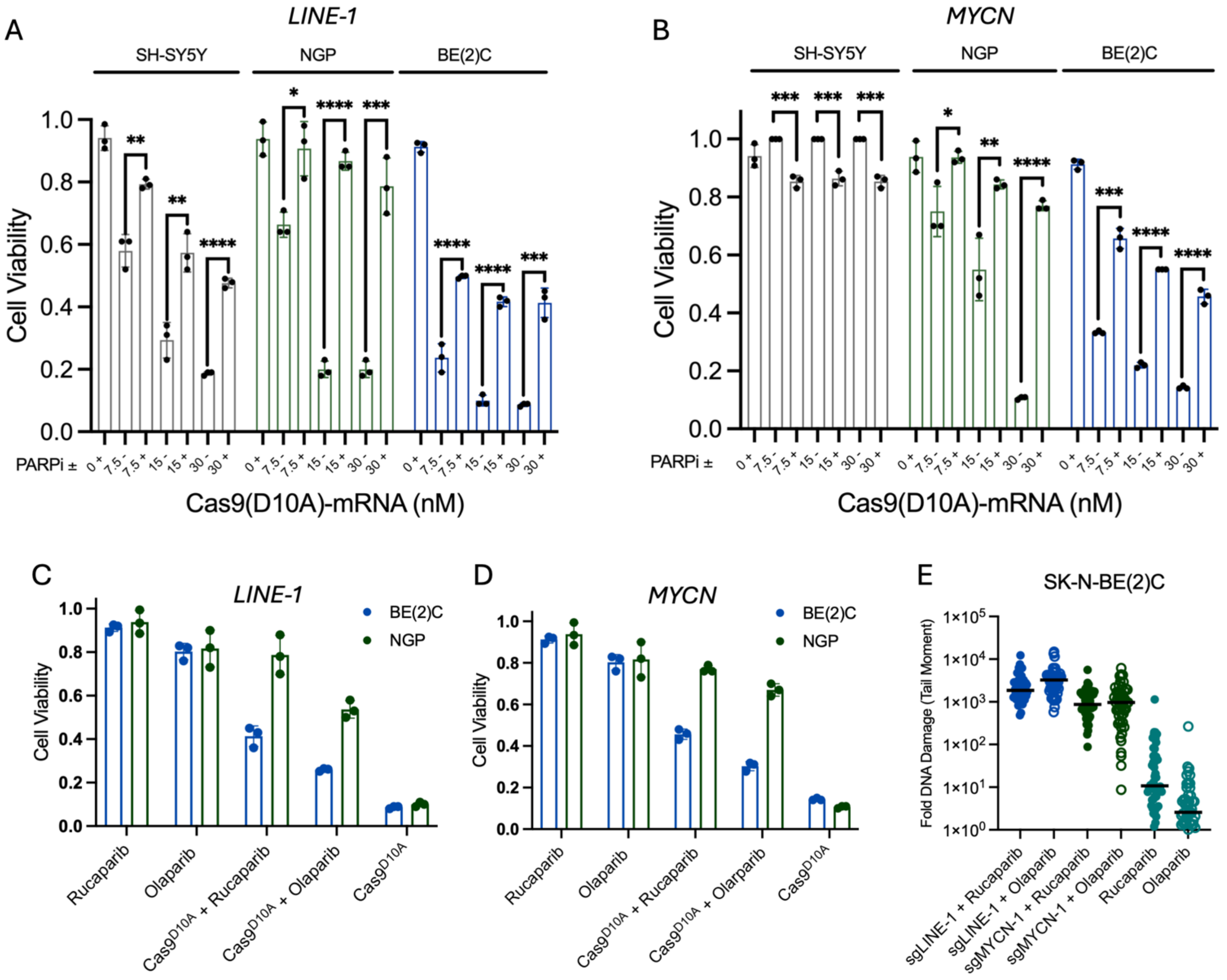

Supplementary Figure 17. Cas9^{D10A}-mediated neuroblastoma cell-killing is attenuated in combination with PARP inhibitors. A & B) Impact on cell viability of treatment of SK-N-BE(2)C, NGP, and SH-SY5Y cells expressing *LINE-1* or *MYCN* targeting sgRNA with increasing doses of Cas9^{D10A}-mRNA in the absence or presence of a PARP inhibitor, rucaparib (10 µM). Inhibition of PARP1 in the presence of Cas9^{D10A} is protective, as indicated by the reduced cell-killing efficacy of Cas9^{D10A} at 3-days post-treatment (n = 3). Data are presented as mean ± s.d. normalized relative to viability of cells expressing *AAVS1* targeting sgRNA treated with Cas9^{D10A}. Data were analyzed multiple unpaired t-tests; ns, P > 0.05; *, P ≤ 0.05; ** P ≤ 0.01; ***, P ≤ 0.001; ****, P ≤ 0.0001. **C & D)** Similar protective effects for PARP1 inhibitors rucaparib (10 µM) and olaparib (10 µM) were observed in *MYCN*-amplified SK-N-BE(2)C and NGP cells when targeting *LINE-1* or *MYCN* loci with Cas9^{D10A}-mRNA (30 µM; n = 3). **E)** SK-N-BE(2)C cells assessed for changes in Cas9^{D10A}-mediated DNA damage when applied in combination with different PARP inhibitors (10 µM). Individual cells were assessed for DNA damage at 3-days post-treatment by alkaline comet assay. Data are presented as individual data points around the median (black line; n = 50).

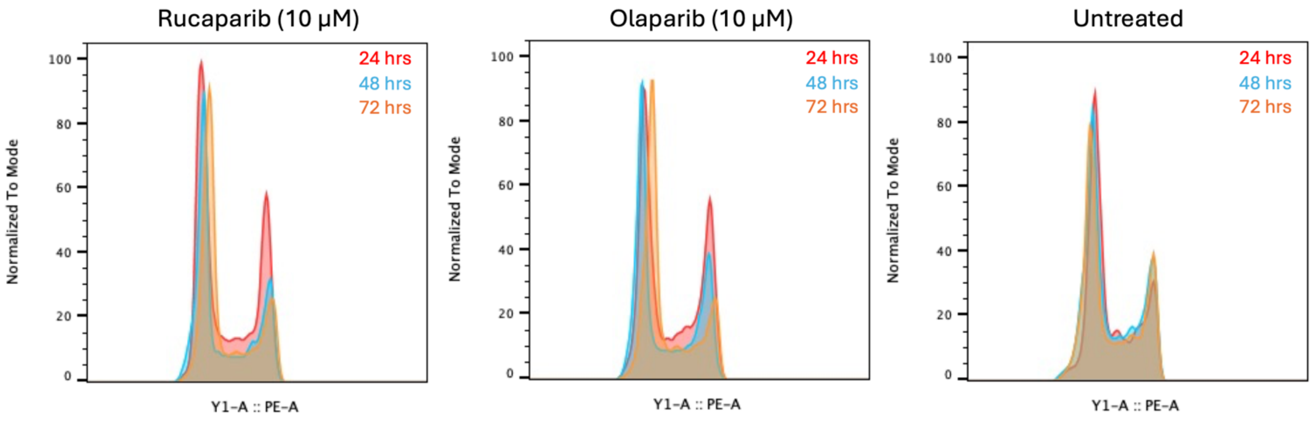

Supplementary Figure 18. Evaluating the effects of low-dose PARP inhibitors on cell cycle progression in *MYCN*-amplified SK-N-BE(2)C cells. Representative histograms of flow cytometric cell cycle analysis of SK-N-BE(2)C cells supplemented with PARP inhibitors at their respective IC_{50}. SK-N-BE(2)C cells were incubated with rucaparib (10 µM) or olaparib (10 µM) in the absence of $Cas9^{D10A}$ and monitored for abberations in cell cycle progression at 1-, 2-, and 3-days (n = 3).

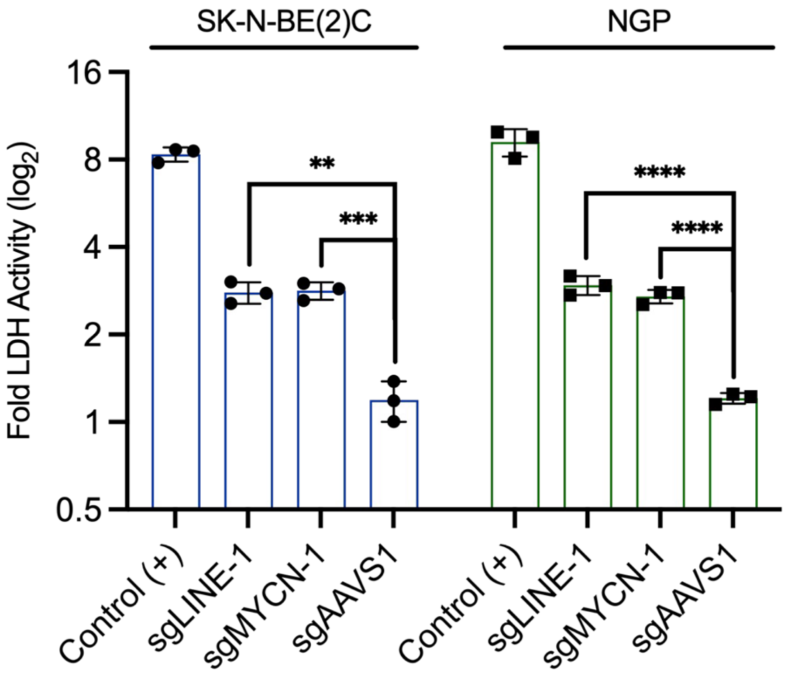

Supplementary Figure 19. *MYCN*-amplified neuroblastoma cells display an enrichment in extracellular LDH, a canonical marker of necrotric cell death, following treatment with Cas9^{D10A} targeting amplified loci. Lactate dehydrogenase (LDH) activity assessment in in *MYCN*-amplified SK-N-BE(2)C and NGP cells expressing *LINE-1*, *MYCN*, or *AAVS1* sgRNA at 3-days post-treatment with Cas9^{D10A}-mRNA (30 nM; n = 3). Data are presented as mean ± s.d. and were analyzed by multiple unpaired t-tests; ns, P > 0.05; *, P ≤ 0.05; ** P ≤ 0.01; ***, P ≤ 0.001; ****, P ≤ 0.0001 using untreated cells as a baseline control.

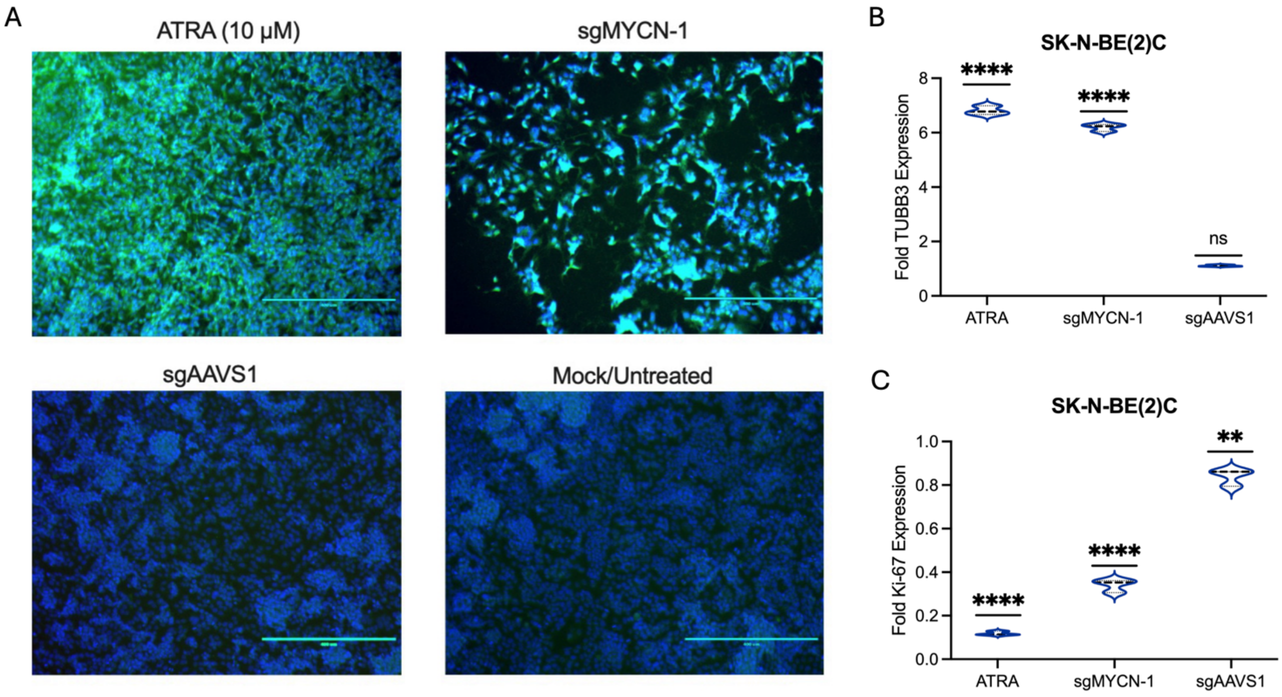

Supplementary Figure 20. Surviving *MYCN*-amplified neuroblastoma cells post Cas9^{D10A} nickase treatment demonstrate markers of neuronal differentiation. A) Representative images of Cas9^{D10A} or all-trans retinoic acid (ATRA) treated SK-N-BE(2)C cells displaying TUBB3 expression (AF488, green) and cell nuclei (Hoecsht 33342, blue). **B)** TUBB3 expression analysis of SK-N-BE(2)C cells treated with Cas9^{D10A}-mRNA (30 nM) targeting *MYCN* or *AAVS1*, and SK-N-BE(2)C cells treated with ATRA (10 μM) relative to an untreated control at 5-days post-treatment (n = 3). **C)** Ki-67 expression analysis of SK-N-BE(2)C cells treated with Cas9^{D10A}-mRNA (30 nM) targeting *MYCN* or *AAVS1*, and SK-N-BE(2)C cells treated with ATRA (10 μM) relative to an untreated control at 5-days post-treatment (n = 3). In each violin plot the single horizontal dashed line represents the median, the two horizontal dotted lines represent the upper and lower quartiles. Data were normalized to untreated cells and analyzed by multiple unpaired t-tests; ns, P > 0.05; *, P ≤ 0.05; ** P ≤ 0.01; ***, P ≤ 0.001; ****, P ≤ 0.0001 using untreated cells as a baseline control.

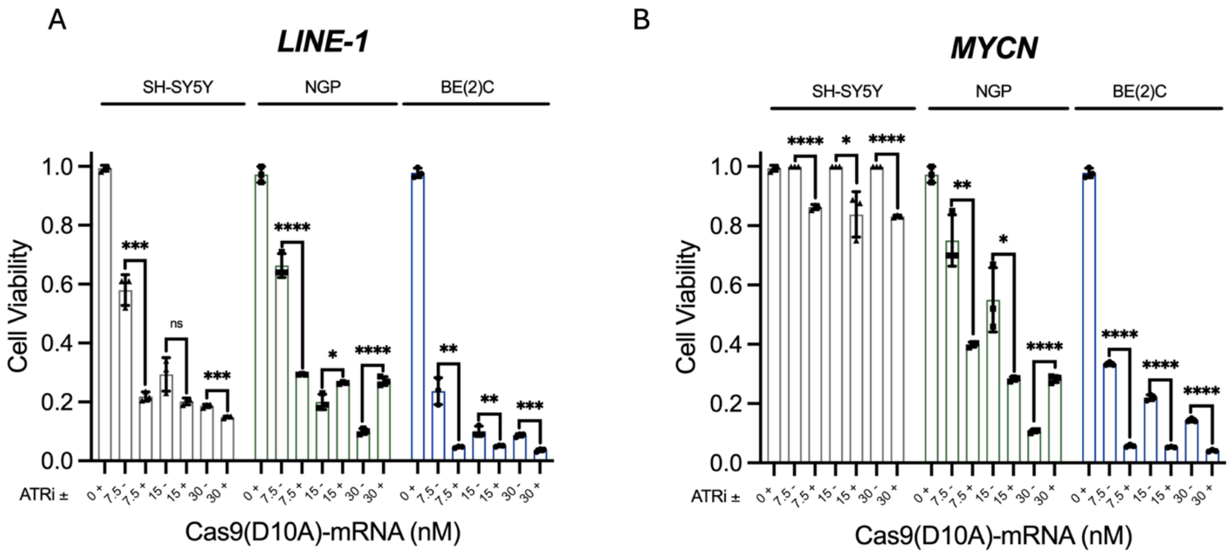

Supplementary Figure 21. ATR inhibitors enhance the efficacy of Cas9^{D10A}-mediated cell-killing at lower concentrations of Cas9^{D10A}-mRNA. A & B) SK-N-BE(2)C, NGP, and SH-SY5Y cells expressing A) *LINE-1* or B) *MYCN* targeting sgRNA were treated with Cas9^{D10A}–mRNA at increasing doses in the absence or presence of an ATR inhibitor, berzosertib (M6620, VX-970, VE-822; 20 nM). Co-treatment with an ATRi potentiated Cas9^{D10A}-mediated cell-killing across all cell lines at lower concentrations of Cas9^{D10A} at 3-days post-treatment (n = 3). Only a modest reduction in cell viability was observed in the *MYCN* non-amplified cell line (SH-SY5Y) for Cas9^{D10A} targeting the *MYCN* locus in the presence of the ATRi. Data are presented as mean ± s.d. normalized relative to viability of cells expressing *AAVS1* targeting sgRNA treated with Cas9^{D10A}. Data were analyzed using multiple unpaired t-tests; ns, $P > 0.05$; *, $P \leq 0.05$; ** $P \leq 0.01$; ***, $P \leq 0.001$; ****, $P \leq 0.0001$.

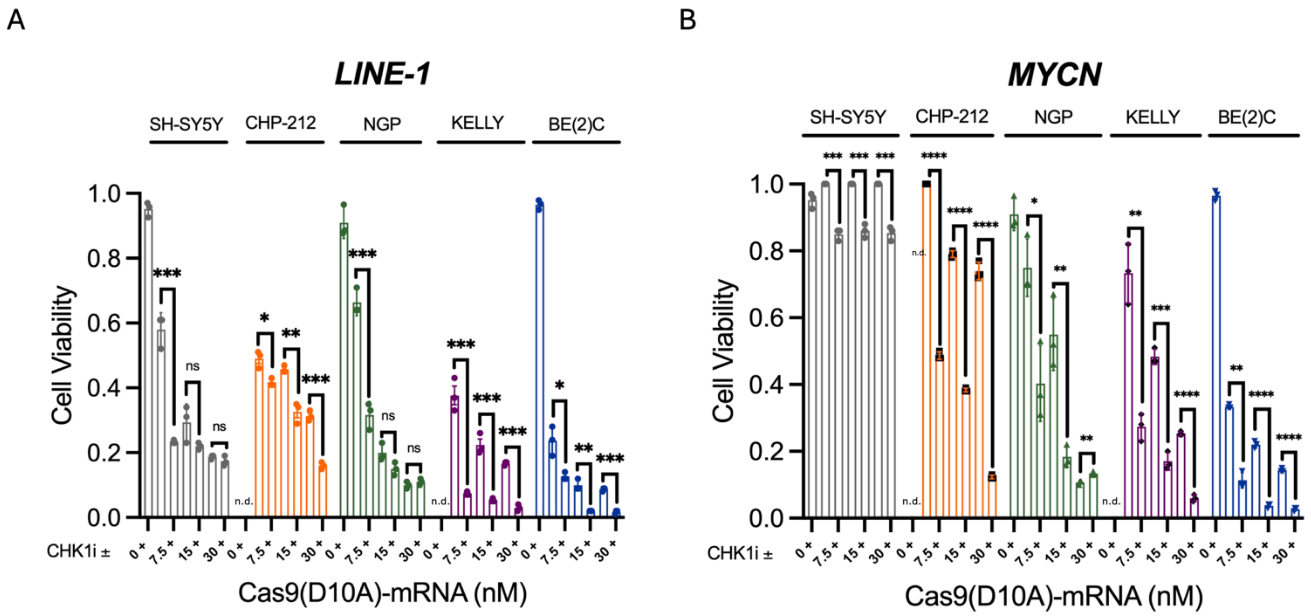

Supplementary Figure 22. Assessing Cas9^{D10A}-mediated cellular toxicity in combination with a CHK1 inhibitor in *MYCN*-amplified neuroblastoma cells. A & B) *MYCN*-amplified, SK-N-BE(2)C, KELLY, NGP, CHP-212, and non-*MYCN* amplified SH-SY5Y cells expressing A) *LINE-1* or B) *MYCN* targeting sgRNA were treated with Cas9^{D10A}-mRNA (7.5, 15, or 30 nM; increase in concentration marked by black triangle) without or with a CHK1 inhibitor, MK8776 (500 nM). Co-treatment with a CHK1i potentiated Cas9^{D10A}-mediated cell-killing substantially across all cell lines at 3-days post-treatment when targeting *LINE-1* or when targeting *MYCN* in *MYCN*-amplified cell lines (n = 3). Only a modest reduction in cell viability was observed in the *MYCN* non-amplified cell line (SH-SY5Y) for Cas9^{D10A} targeting the *MYCN* locus in the presence of the CHK1i. Data are presented as mean ± s.d. normalized relative to viability of cells expressing *AAVS1* targeting sgRNA treated with Cas9^{D10A}. Data were analyzed using multiple unpaired t-tests; ns, $P > 0.05$; *, $P \leq 0.05$; ** $P \leq 0.01$; ***, $P \leq 0.001$; ****, $P \leq 0.0001$ using *AAVS1* sgRNA expressing cells as a baseline control.

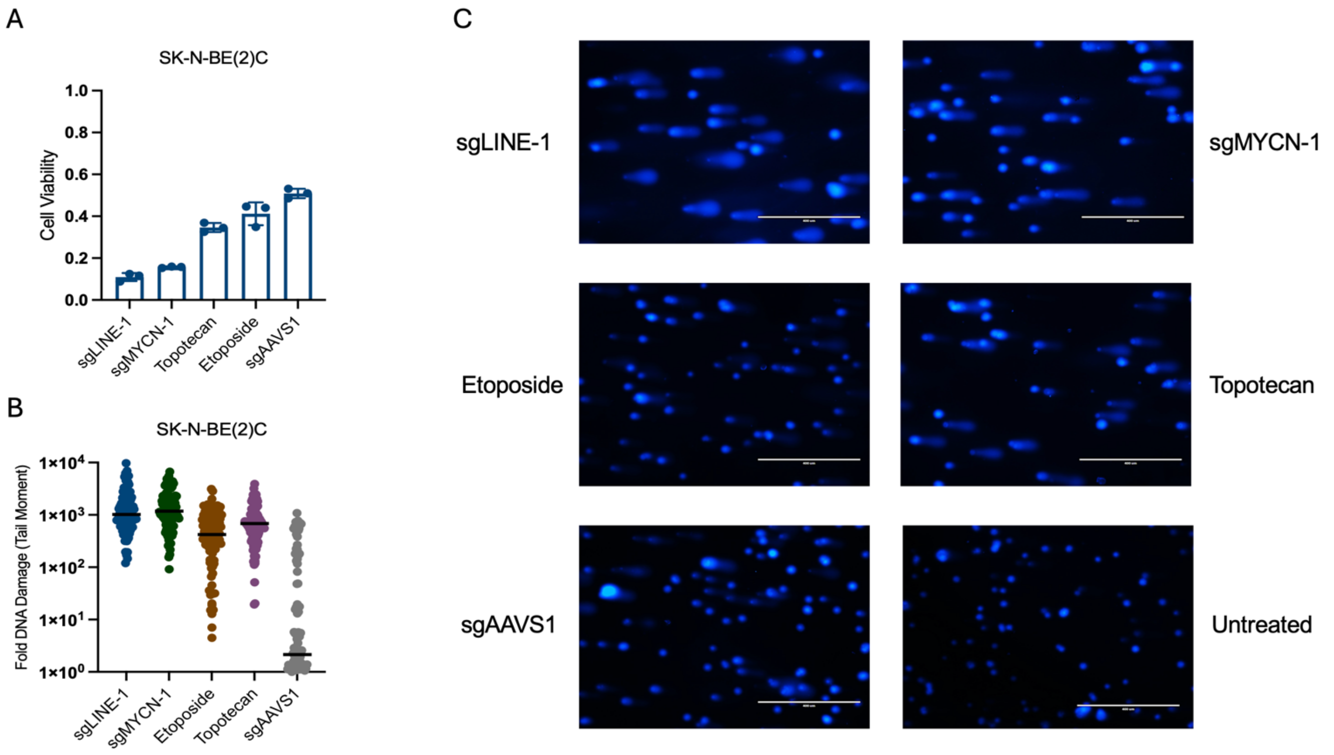

Supplementary Figure 23. Cas9^{WT} nuclease demonstrates effective neuroblastoma cell-killing, but with greater non-specific toxicity. A) Impact on cell viability of SK-N-BE(2)C post-treatment with Cas9^{WT}-mRNA (30 nM). Topoisomerase inhibitors topotecan (60 nM) and etoposide (1 µM) are included as positive controls. Changes in cell viability were assessed at 3-days post-treatment with Cas9^{WT} targeting *LINE-1*, *MYCN*, or *AAVS1*. Cas9^{WT} displayed effective cell-killing when targeting *LINE-1* or *MYCN*, but demonstrated an appreciable degree of non-specific toxicity when targeted to *AAVS1*, a non-amplified locus. Data are presented as mean ± s.d. normalized relative to the viability of cells expressing *AAVS1* targeting sgRNA treated with Cas9^{D10A}. **B)** SK-N-BE(2)C cells assessed by alkaline comet for DNA damage at 3-days post-treatment with Cas9^{WT}. Data are presented as individual data points around the median (black line; n = 100). **C)** Representative images of Cas9^{WT}-treated cell comets from panel B.

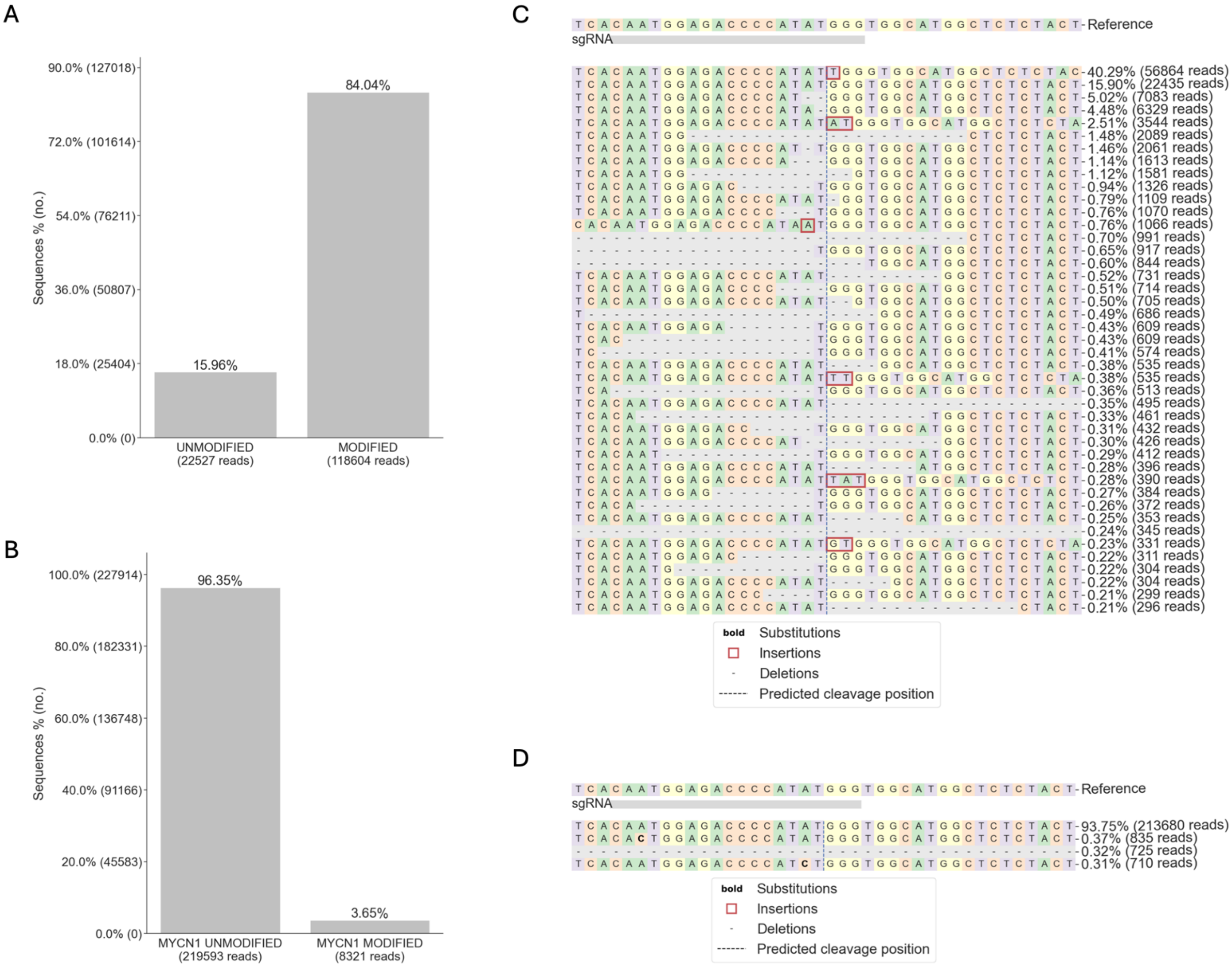

Supplementary Figure 24. Indel frequencies at *MYCN* target site of Cas9^{D10A}-treated neuroblastoma cells are low. A & B) Amplicon-sequencing of the sgMYCN-1 target site from genomic DNA isolated from surviving SK-N-BE(2)C cells expressing sgMYCN-1 at 3-days post-treatment with A) Cas9^{WT}-mRNA (30 nM) or B) Cas9^{D10A}-mRNA (30 nM). Outcomes demonstrate the local mutagenic burden of Cas9^{D10A} treatment is low when targeting amplified loci. Target site depletion post-treatment is negligible, likely permitting the continued use of an sgRNA in repeat-dosing to promote cell death. **C & D)** Allele frequency tables reveal an array of sgMYCN-1 target site sequence alterations post-treatment with C) Cas9^{WT}, whereas the mutagenic burden of D) Cas9^{D10A} is low with larger deletions as highest frequency sequence-alteration, consistent with resection at the site of a seDSB or deDSB.

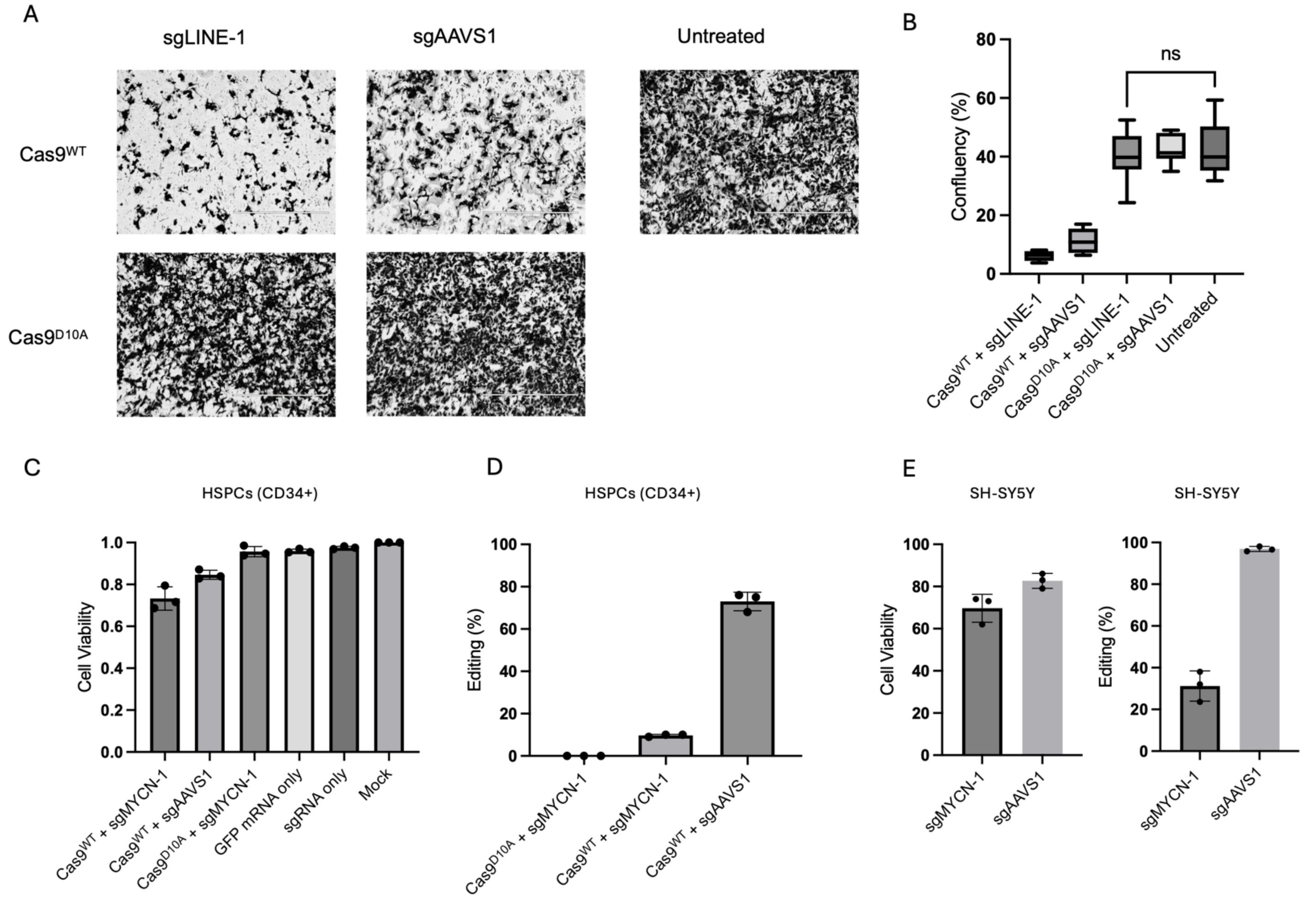

Supplementary Figure 25. Cas9^{D10A}-mediated cell-killing demonstrates negligible non-specific toxicity in post-mitotic cells and hematopoeitic stem and progenitor cells. A) Representative image of neuronally differentiated SK-N-BE(2)C cells stained with crystal violet (grayscale) at 3-days post-transfection with either Cas9^{WT} or Cas9^{D10A}-mRNA. **B)** Cell confluency was used as a metric to assess cellular toxicity in neuronally differentiated SK-N-BE(2)C cells when targeting *LINE-1* or *AAVS1* with Cas9^{WT} or Cas9^{D10A}. Neuronally differentiated SK-N-BE(2)C cells display high sensitivity to Cas9^{WT}-mediated DSBs at both target sites. No significant cellular toxicity was observed when targeting *LINE-1* ($P > 0.05$) or *AAVS1* with Cas9^{D10A}. In each box plot the horizontal line represents the median, the top and bottom of the box represent the upper and lower quartiles, respectively, and the top and bottom whiskers represent the maximum and minimum values, respectively. Data were analyzed by multiple unpaired t-tests; ns, $P > 0.05$; *, $P \leq 0.05$; ** $P \leq 0.01$; ***, $P \leq 0.001$; ****, $P \leq 0.0001$ using untreated cells as a baseline control. **C)** Impact on cell viability of hematopoietic stem and progenitor cells (HSPCs; CD34+) post-treatment with Cas9^{WT} or Cas9^{D10A}-mRNA (30 nM) and a synthetic sgRNA (30 µM) targeting *MYCN* or *AAVS1*. Changes in cell viability assessed at 3-days post-treatment with Cas9^{WT} or Cas9^{D10A} relative to mRNA only (GFP – mRNA), sgRNA only (1:1, sgMYCN-1 + sgAAVS1), or electroporation only (mock) controls (n = 3). **D)** Target site editing rates in surviving HSPCs at 3-days post-treatment with Cas9^{WT} or Cas9^{D10A} targeting *MYCN* or *AAVS1* determined by Sanger sequencing (n = 3). **E)** Cell viability and target site editing rate in *MYCN* non-amplified SH-SY5Y cells expressing *MYCN* or *AAVS1* targeting sgRNA at 3-days post-treatment with Cas9^{WT} – mRNA (30 nM) as a control (n = 3).

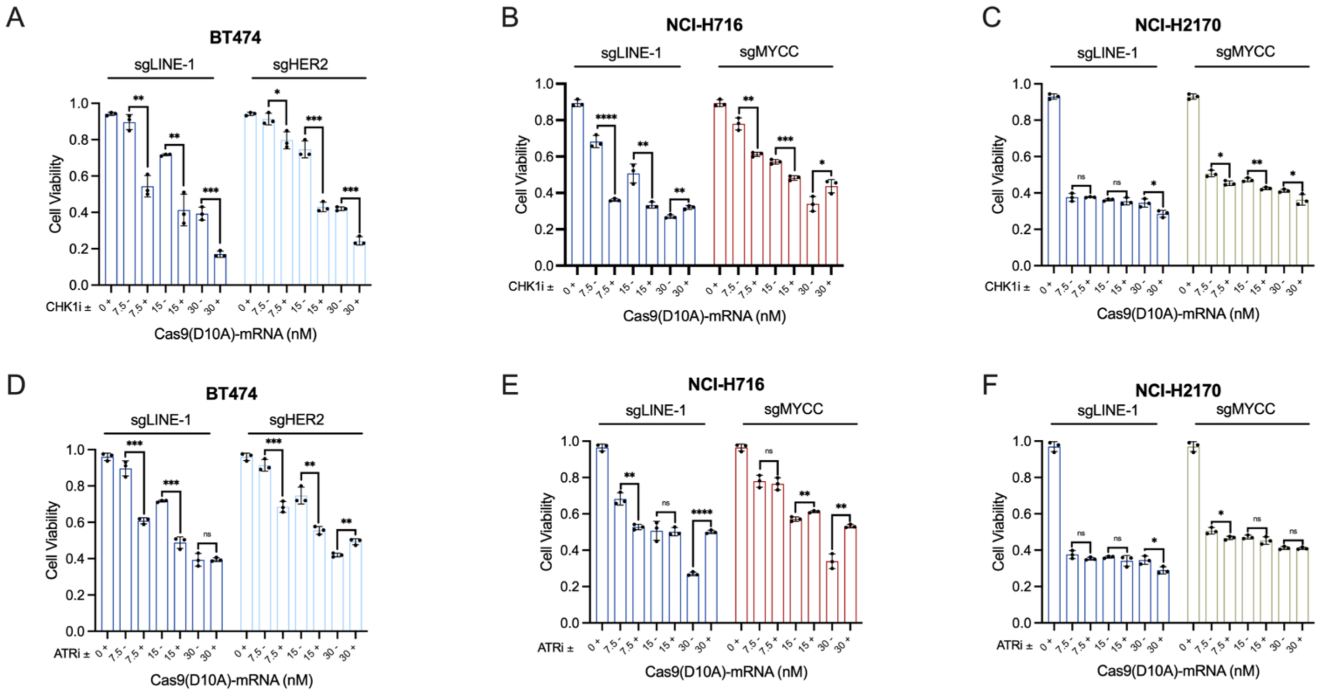

Supplementary Figure 26. Cas9^{D10A} demonstrates enhanced cell-killing activity in combination with CHK1 inhibitors in *ERBB2* (HER2)-amplified breast cancer, and *MYC*-amplified non-small cell lung cancer and colorectal cancer cells. A – C) BT-474, NCI-H716, or NCI-H2170 cells were treated with Cas9^{D10A}-mRNA at increasing doses in the absence or presence of the CHK1 inhibitor, MK8776 (500 nM) and assessed for changes in cell viability at 3-days post-treatment (n = 3). Co-treatment with a CHK1i potentiated Cas9^{D10A}-mediated cell-killing across most concentrations of Cas9^{D10A}. **D – F)** BT-474, NCI-H716, or NCI-H2170 cells were treated with Cas9^{D10A}-mRNA at increasing doses in the absence or presence of the ATR inhibitor berzosertib (M6620, VX-970, VE-822; 20 nM), respectively, and assessed for changes in cell viability at 3-days post-treatment (n = 3). Co-treatment of BT-474 cells with an ATRi potentiated Cas9^{D10A}-mediated cell-killing at lower concentrations of Cas9^{D10A}, whereas it dampened the effectiveness of Cas9^{D10A} at higher concentrations (n = 3). Data are presented as mean ± s.d. normalized relative to viability of cells expressing *AAVS1* targeting sgRNA treated with Cas9^{D10A}. Data were analyzed using multiple unpaired t-tests; ns, $P > 0.05$; *, $P \le 0.05$; ** $P \le 0.01$; ***, $P \le 0.001$; ****, $P \le 0.0001$ using *AAVS1* sgRNA expressing cells as a baseline control.

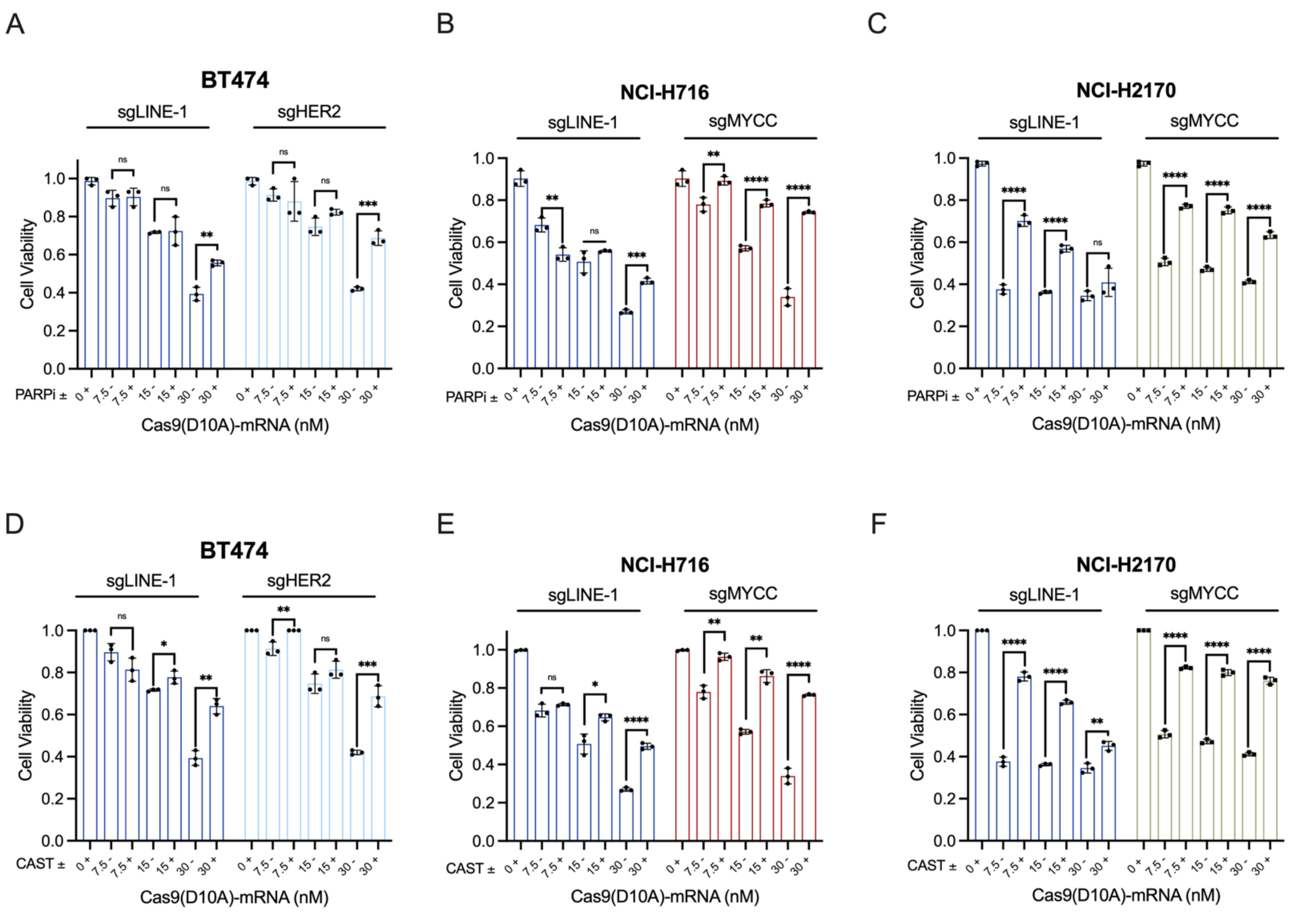

Supplementary Figure 27. Cas9^{D10A}-mediated toxicity attenuated by PARP or calpain inhibitors in *ERBB2* (HER2)-amplified breast cancer cells, *MYC*-amplified non-small cell lung cancer and colorectal cancer cells. A – C) Treatment of BT-474, NCI-H2170, or NCI-H716 cells expressing *LINE-1, ERBB2* (HER2), or *MYC* targeting sgRNA with Cas9^{D10A}-mRNA at increasing doses in the absence or presence of a PARP inhibitor, rucaparib (10 μM; n = 3). **D – F)** Treatment of BT-474, NCI-H2170, or NCI-H716 cells expressing *LINE-1, ERBB2* (HER2), or *MYC* targeting sgRNA with Cas9^{D10A}-mRNA at increasing doses in the absence or presence of a calpain inhibitor, calpastatin (CAST; 20 nM; n = 3). Inhibition of PARP1 or calpains in the presence of Cas9^{D10A} is protective, as indicated by the reduced cell-killing efficacy of Cas9^{D10A} in BT-474, NCI-H2170, and NCI-H716 cells at 3-days post-treatment. Data are presented as mean ± s.d. normalized relative to viability of cells expressing *AAVS1* targeting sgRNA treated with Cas9^{D10A}. Data were analyzed using multiple unpaired t-tests; ns, $P > 0.05$; *, $P \leq 0.05$; ** $P \leq 0.01$; ***, $P \leq 0.001$; ****, $P \leq 0.0001$ using *AAVS1* sgRNA expressing cells as a baseline control.
