# Supplementary material for: Selective targeting of genome amplifications and repeat elements by CRISPR-Cas9 nickases to promote cancer cell death
Source: Nat Commun. 2025 Jun 2;16:5126. doi: 10.1038/s41467-025-60160-2 (PMC12130199; doi:10.1038/s41467-025-60160-2)
Supplement: Supplementary file 2 — Reporting Summary [file 41467_2025_60160_MOESM2_ESM.pdf]

nature portfolio

Corresponding author(s): Scot A. Wolfe

Last updated by author(s): Apr 18, 2025

Reporting Summary

Nature Portfolio wishes to improve the reproducibility of the work that we publish. This form provides structure for consistency and transparency in reporting. For further information on Nature Portfolio policies, see our Editorial Policies and the Editorial Policy Checklist.

Statistics

For all statistical analyses, confirm that the following items are present in the figure legend, table legend, main text, or Methods section.

n/a	Confirmed	
☐	☒	The exact sample size (*n*) for each experimental group/condition, given as a discrete number and unit of measurement
☐	☒	A statement on whether measurements were taken from distinct samples or whether the same sample was measured repeatedly
☐	☒	The statistical test(s) used AND whether they are one- or two-sided<br>*Only common tests should be described solely by name; describe more complex techniques in the Methods section.*
☐	☒	A description of all covariates tested
☒	☐	A description of any assumptions or corrections, such as tests of normality and adjustment for multiple comparisons
☐	☒	A full description of the statistical parameters including central tendency (e.g. means) or other basic estimates (e.g. regression coefficient) AND variation (e.g. standard deviation) or associated estimates of uncertainty (e.g. confidence intervals)
☐	☒	For null hypothesis testing, the test statistic (e.g. *F*, *t*, *r*) with confidence intervals, effect sizes, degrees of freedom and *P* value noted<br>*Give P values as exact values whenever suitable.*
☒	☐	For Bayesian analysis, information on the choice of priors and Markov chain Monte Carlo settings
☒	☐	For hierarchical and complex designs, identification of the appropriate level for tests and full reporting of outcomes
☒	☐	Estimates of effect sizes (e.g. Cohen's *d*, Pearson's *r*), indicating how they were calculated

Our web collection on statistics for biologists contains articles on many of the points above.

Software and code

Policy information about availability of computer code

Data collection	No data collection software was used in this study
Data analysis	Data analysis was performed using GraphPad Prism(10.2.3), FlowJo (10.8.1), and/or ImageJ (NIH; ImageJ2 v.2.14.0/1.54f)

For manuscripts utilizing custom algorithms or software that are central to the research but not yet described in published literature, software must be made available to editors and reviewers. We strongly encourage code deposition in a community repository (e.g. GitHub). See the Nature Portfolio guidelines for submitting code & software for further information.

Data

Policy information about availability of data

All manuscripts must include a data availability statement. This statement should provide the following information, where applicable:

- Accession codes, unique identifiers, or web links for publicly available datasets
- A description of any restrictions on data availability
- For clinical datasets or third party data, please ensure that the statement adheres to our policy

The authors declare that supporting data for this study is available within the article, extended data, and/or supplementary materials. Illumina sequencing data has been submitted to the Sequence Read Archive (PRJNA1251614). Source data are provided as a Source Data file.

Research involving human participants, their data, or biological material

Policy information about studies with human participants or human data. See also policy information about sex, gender (identity/presentation), and sexual orientation and race, ethnicity and racism.

Reporting on sex and gender	Not Applicable
Reporting on race, ethnicity, or other socially relevant groupings	Not Applicable
Population characteristics	Not Applicable
Recruitment	Not Applicable
Ethics oversight	Not Applicable

Note that full information on the approval of the study protocol must also be provided in the manuscript.

Field-specific reporting

Please select the one below that is the best fit for your research. If you are not sure, read the appropriate sections before making your selection.

[x] Life sciences [ ] Behavioural & social sciences [ ] Ecological, evolutionary & environmental sciences

For a reference copy of the document with all sections, see nature.com/documents/nr-reporting-summary-flat.pdf

Life sciences study design

All studies must disclose on these points even when the disclosure is negative.

Sample size	Sample sizes were determined based on literature precedence for genome editing experiments. For our cellular experiments, ≥1x10^5 cells were used for Cas9 nickase treatment in culture cells with delivery by electroporation or transfection. For amplicon deep sequencing, we desired a target depth of ~10,000 reads/sample to determine genome editing rates, which is typical for studies reported within the field. All cell-based Cas9 nickase treatment experiments were evaluated in at least biological triplicates (n = 3) to ensure the reproducibility.
Data exclusions	No data was excluded from analyses
Replication	All experiments were prepared and conducted individually. Replicates, where applicable, were conducted a minimum of three times. All replications were successful.
Randomization	No randomization of samples or treatment groups was applicable (or used) in this study. In our studies, we utilized control groups for reference with regards to Cas9 nickase activity. When necessary we performed titration of reagents to define an optimal range for performing our experiments.
Blinding	No researchers were blinded as to groups during experiments, data collection, or data analyses.

Reporting for specific materials, systems and methods

We require information from authors about some types of materials, experimental systems and methods used in many studies. Here, indicate whether each material, system or method listed is relevant to your study. If you are not sure if a list item applies to your research, read the appropriate section before selecting a response.

Materials & experimental systems

n/a	Involved in the study	
[ ]	[x]	Antibodies
[ ]	[x]	Eukaryotic cell lines
[x]	[ ]	Palaeontology and archaeology
[x]	[ ]	Animals and other organisms
[x]	[ ]	Clinical data
[x]	[ ]	Dual use research of concern
[x]	[ ]	Plants

Methods

n/a	Involved in the study	
[x]	[ ]	ChIP-seq
[ ]	[x]	Flow cytometry
[x]	[ ]	MRI-based neuroimaging

Antibodies

Antibodies used	N-Myc monoclonal antibody (NCM-II 100; Invitrogen, CAT#MA1-170, LOT#YJ384835), PARP1 polyclonal antibody (Invitrogen,

	CAT#PAS-34803, LOT#ZA4162159A ;ZB4234889C); Poly ADP-ribose antibody (Millipore Sigma, CAT#MABC547, clone10H, LOT#4080161) ;Caspase3 polyclonal antibody (Invitrogen, CAT#PA5-77887, LOT#ZA4162117A); phospho-EXO1(Ser746) (MilliporeSigma, CAT# ABE1066, LOT#3502123); anti-gamma-H2AX polyclonal antibody (Bethyl Laboratories, CAT#A300-081A, LOT#23); phospho-CHK1(Ser345) polyclonal antibody (Invitrogen, CAT#PA5-34625, LOT#Y14045763E; Y14042088B); phospho-RPA32 (Ser33) polyclonal antibody(Invitrogen, CAT#PA5-39809, LOT#YJ4086171) RPA2 monoclonal antibody (Invitrogen, CAT#MA1-26418, clone 9H8, LOT#YJ4089392); Beta-actin mouse monoclonal antibody (CellSignaling Technology, CAT#3700, clone8H10D10, LOT#21); anti-rabbit IgG, HRP-conjugated antibody (Cell Signaling Technology, CAT#7074, LOT#33); anti-mouse IgG, HRP-conjugated antibody (Cell Signaling Technology, CAT#7076, LOT#38); Beta-3 tubulin polyclonal antibody (Invitrogen, CAT#PA5-25655, LOT#ZA4178911A); Ki-67 recombinant monoclonal antibody (Invitrogen, CAT#MA5-14520, clone SP6, LOT#YL4156571); goat anti-rabbit IgG (H+L) AF488 (Invitrogen, CAT#A32731, LOT#YB367680) goat anti-rabbit IgG (H+L) AF594 (Invitrogen, CAT#A11012, LOT#2616076); Mu-Calpain polyclonal antibody (Invitrogen, CAT#PA5-17547, LOT#YI4050847A); Calpain 2 polyclonal antibody (Invitrogen, CAT#PA517494, LOT#YL4149738).
Validation	Antibodies were validated by the manufacturer and supported by their supplied data sheets

Eukaryotic cell lines

Policy information about cell lines and Sex and Gender in Research

Cell line source(s)	SK-N-BE(2)C [CRL-2268], CHP-212 [CRL-2273], IMR-32 [CCL-127], SH-SY5Y [CRL-2266], BT-474 [HBT-20], NCI-H2170 [CRL-5928], NCI-H716 [CCL-251], and HEK293T [CRL-3216] cells were sourced through the American Type Culture Collection (ATCC). KELLY [ACC 355] and NGP [ACC 676] cells were sourced through the Leibniz Institute (DSMZ). Primary CD34+ hematopoietic stem and progenitor cells (HSPCs) isolated from human cord blood were sourced as a gift from Dr. Michael Brehm (University of Massachusetts Chan Medical School), where the cord blood was provided by the medical staff of the University of Massachusetts Memorial Umbilical Cord Blood Donation Program.
Authentication	Independent authentication of each cell line was not performed after receipt from the vendor(s). Focal amplification of the MYCN , ALK, ERBB2, or MYC gene was independently verified by qPCR and found to be in general agreement with other literature reported values for each corresponding cell line.
Mycoplasma contamination	All cell lines were tested routinely for mycoplasma contamination using the e-Myco VALiD mycoplasma PCR detection kit (LiliF Diagnostics). All cells used in experiments were negative for mycoplasma contamination.
Commonly misidentified lines (See ICLAC register)	No commonly misidentified cell lines were used in this study.

Plants

Seed stocks	Not Applicable
Novel plant genotypes	Not Applicable
Authentication	Not Applicable

Flow Cytometry

Plots

Confirm that:

- [x] The axis labels state the marker and fluorochrome used (e.g. CD4-FITC).
- [x] The axis scales are clearly visible. Include numbers along axes only for bottom left plot of group (a 'group' is an analysis of identical markers).
- [ ] All plots are contour plots with outliers or pseudocolor plots.
- [x] A numerical value for number of cells or percentage (with statistics) is provided.

Methodology

Sample preparation	Human neuroblastoma cells (SK-N-BE(2)C) were used for flow cytometry experiments. Cell cycle analyses were conducted at 1-, 2-, and 3-days post-treatment. SK-N-BE(2)C cells were harvested and fixed in 95% ethanol overnight at 4 deg C, washed twice with PBS (1X), and stained with a propidium iodide and RNaseA solution for 30 minutes at 37 deg C. For micronuclei detection at 3-days post-treatment, cells were stained with Hoecsht 33342 for 30 minutes at room temperature before being harvested. Cells were pelleted and resuspended in a partial lysis buffer to isolate nuclei. All samples were filtered to remove aggregates using cell strainers (40 um) prior to analysis.

Instrument	MACSQuant Analyzer10 Flow Cytometer (Mitenyi Biotech)
Software	FlowJo (10.8.1) software
Cell population abundance	All post-flow cytometry analyses were conducted on samples that recorded 10,000 or more events.
Gating strategy	For cell cycle analysis, a homogeneous single-cell population was acquired by SSC-A/FSC-A gating. Additional FSC-A/dye-A was used to acquire distinct 2n/4n cell populations. Presentation of data converted to a histogram prior to analysis. For micronuclei detection, a homogeneous single-cell population was acquired by FSC-A/SSC-A gating. Further FSC-A/dye-A was used to identify intact nuclei. The population of intact nuclei was verified by the visualization of distinct 2n/4n nuclei. The presence of micronuclei was further validated by fluorescent microscopy.

☐ Tick this box to confirm that a figure exemplifying the gating strategy is provided in the Supplementary Information.
